# Effect of Mexican Propolis on Wound Healing in a Murine Model of Diabetes Mellitus

**DOI:** 10.3390/ijms25042201

**Published:** 2024-02-12

**Authors:** Octavio Canales-Alvarez, Maria Margarita Canales-Martinez, Pilar Dominguez-Verano, Daniela Balderas-Cordero, Eduardo Madrigal-Bujaidar, Isela Álvarez-González, Marco Aurelio Rodriguez-Monroy

**Affiliations:** 1 Laboratorio de Genética, Escuela Nacional de Ciencias Biológicas, Instituto Politécnico Nacional, Av. Wilfrido Massieu s/n, Zacatenco, Ciudad de México 07738, Mexico; octaviocanalesa@gmail.com (O.C.-A.); edumadrigal.bujaidar@gmail.com (E.M.-B.); isela.alvarez@gmail.com (I.Á.-G.); 2 Laboratorio de Investigación Biomédica en Productos Naturales, Carrera de Medicina, UNAM, FES Iztacala, Avenida de los Barrios Número 1, Tlalnepantla 54090, Estado de México, Mexico; pilardomver@hotmail.com (P.D.-V.); daniebalcord@gmail.com (D.B.-C.); 3 Laboratorio de Farmacognosia, UBIPRO, UNAM, FES Iztacala, Avenida de los Barrios Número 1, Tlalnepantla 54090, Estado de México, Mexico; dra.margaritacanales@gmail.com

**Keywords:** diabetes mellitus, wound healing, propolis, inflammation, antioxidant

## Abstract

Diabetes mellitus (DM) affects the wound healing process, resulting in impaired healing or aberrant scarring. DM increases reactive oxygen species (ROS) production, fibroblast senescence and angiogenesis abnormalities, causing exacerbated inflammation accompanied by low levels of TGF—β and an increase in Matrix metalloproteinases (MMPs). Propolis has been proposed as a healing alternative for diabetic patients because it has antimicrobial, anti-inflammatory, antioxidant and proliferative effects and important properties in the healing process. An ethanolic extract of Chihuahua propolis (ChEEP) was obtained and fractionated, and the fractions were subjected to High–Performance Liquid Chromatography with diode–array (HPLC–DAD), High–Performance Liquid Chromatography–Mass Spectrometry (HPLC–MS) and Gas Chromatography–Mass Spectrometry (GC–MS) analyses and 46 compounds were detected. Deep wounds were made in a murine DM model induced by streptozotocin, and the speed of closure and the wound tensile strength were evaluated by the tensiometric method, which showed that ChEEP had similar activity to Recoveron, improving the speed of healing and increasing the wound tensile strength needed to open the wound again. A histological analysis of the wounds was performed using H&E staining, and when Matrix metalloproteinase 9 (MMP9) and α—actin were quantified by immunohistochemistry, ChEEP was shown to be associated with improved histological healing, as indicated by the reduced MMP9 and α—actin expression. In conclusion, topical ChEEP application enhances wound healing in diabetic mice.

## 1. Introduction

In the human body, the skin acts as a chemical, physical and bacterial barrier. A wound is defined as the intentional or accidental rupture of a tissue [1], which could lead to a risk of exposure to external factors. When a wound occurs, a series of biochemical events begin and they overlap in time to repair the damaged tissue; together, these processes constitute wound healing. Wound healing comprises three phases of skin repair: inflammation, proliferation and remodelling [2,3].

The duration of the healing period varies between individuals; however, there are factors that affect this process, such as *diabetes mellitus* (DM) [4]. DM is considered a global public health problem, and this metabolic disease is characterized by the presence of hyperglycaemia as a consequence of defects in insulin secretion [5].

In healing, DM generates a high number of mast cells, proinflammatory macrophages and neutrophils [6]; specifically, neutrophils produce a greater amount of neutrophil extracellular traps (NETs) and macrophages show less effectiveness in the phagocytosis of apoptotic cells and bacteria [7,8,9]. Additionally, the skin microbiome of diabetic patients is altered with a major proportion consisting of pathogens, such as *S. aureus* and *P. aeruginosa* [10]. Another alteration reported in healing caused by DM is an increase in the levels of proteases that continuously destroy the components of the extracellular matrix, growth factors and cytokines, preventing the correct functioning of fibroblasts and causing their late differentiation into myofibroblasts and senescence, which reduces the contraction of the wound [11]. However, an increase in MMP9 levels is necessary during the first days of healing since it stimulates the mobility of keratinocytes and is responsible for the activation of inflammatory mediators and some growth factors [12]. One of these factors is TGF—β, which is a regulator of healing; for example, TGF—β is responsible for the chemotaxis of inflammatory cells and promotes the deposition of the extracellular matrix and the formation of granulation tissue [13]. TGF—β acts as a mediator in the replacement of type III collagen with type I collagen and stimulates the proliferation of keratinocytes. Therefore, if there are defects in TGF—β signalling in diabetic individuals, aberrant healing will occur [14].

Drugs for wound healing have the main effect of stimulating the regeneration of the affected area or promoting an antiseptic environment, which are necessary characteristics that promote proper healing [15,16]. However, a notable challenge in this health sector is that healing treatments are not suitable for all patients due to their high costs. Natural products have proven to be good alternatives to other healing agents.

Propolis is a very complex and varied natural product due to its chemical composition. Its variety depends on the geographical area of origin and the season in which it is collected. The surrounding vegetation also influences it, because bees produce propolis through from the collection of fruits, floral buds, trees and other resinous exudates [15]. Propolis obtained from hives, also known as raw propolis (150 to 300 g per hive), is generally composed of flavonoids (mostly), resins and balms (50%), waxes (30%), essential oils (10%), essences (5%), pollen and mineral salts (5%). Up to 300 compounds have been detected in propolis samples, mainly phenolic compounds, aliphatic acids, esters, aromatic acids, fatty acids, carbohydrates, aldehydes, amino acids, ketones, chalcones, dihydrochalcones, terpenoids, vitamins and inorganic substances [17]. Several biomedical characteristics have been observed in propolis, such as antimicrobial, antioxidant, antiviral, antibiotic, healing, antiparasitic, anaesthetic, antitumour, hepatoprotective, and anti—inflammatory effects, among others [18,19,20,21].

Propolis has been shown to be an alternative treatment for healing in healthy organisms [18]; however, the objective of the present work was to evaluate the healing potential of ChEEP in diabetic mice.

## 2. Results

### 2.1. Chemical Characterization

The organoleptic properties of ChEEP were evaluated according to NOM—003—SAG/GAN—2017 [22] (Table 1).

Additionally, the yield, average antioxidant capacity and total phenol and flavonoid content were evaluated (Table 2); these results were endorsed by NOM—003-SAG/GAN—2017 [22].

To characterize the specific chemicals, the ChEEP was fractionated into a hexanic fraction and an ethyl acetate (EA) fraction, which were subsequently derivatized via the silation method. The EA fraction had a yield of 39.23% (0.3923 g). Preparative chromatography was performed with 100 mg of the EA fraction, which was observed at different wavelengths, resulting in 23 fractions, which were separated from the silica through filtration and evaporation. The antioxidant capacity of the 23 fractions was evaluated by thin-plate bioautography, after which, the substances were subjected to GC–MS, HPLC–DAD and HPLC–MS analyses.

**Figure 1:**
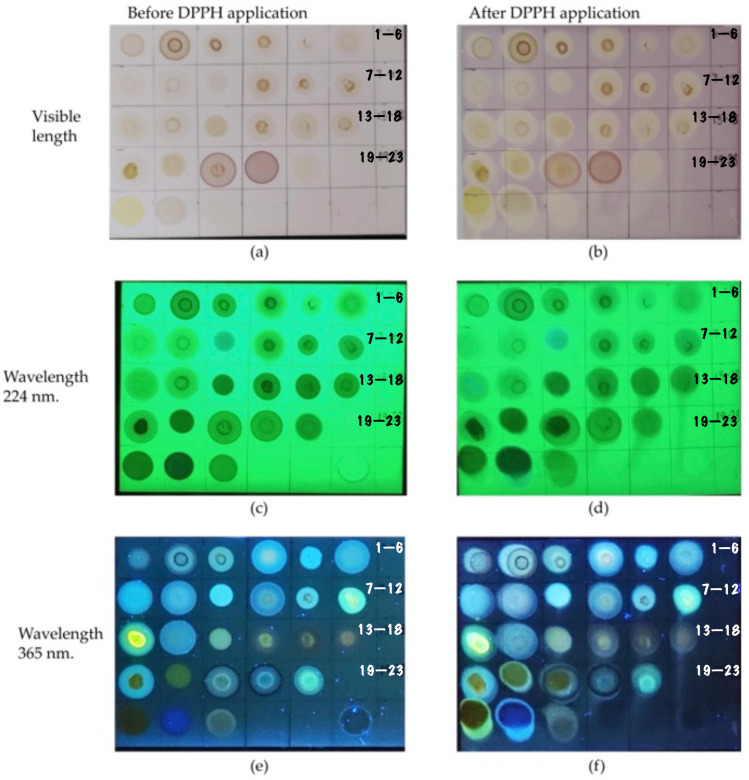
Thin-layer silica chromatographic plate with the seeding points of the 23 fractions compared to the seeding points with DPPH radicals at different wavelengths. (**a**) Application of the fractions in the visible light spectrum, (**b**) fractions with DPPH in the visible light spectrum, (**c**) fractions at 224 nm, (**d**) fractions with DPPH at 224 nm, (**e**) fractions at 365 nm and (**f**) fractions with DPPH at 365 nm.

For the evaluation of antioxidant capacity, the ChEEP fractions showed positive results because all of them presented a pale yellow colour when they came into contact with 2,2–diphenyl–1–picrylhydrazyl (DPPH) (Figure 1).

For the GC–MS analysis, the hexane fraction, EA fraction and derivatized ChEEP were injected, and 27 compounds were identified and are listed in Table 3. Spectra and chromatograms for chemical identification of compounds are available in Figures S1–S3 of the supplementary material.

**Figure 2:**
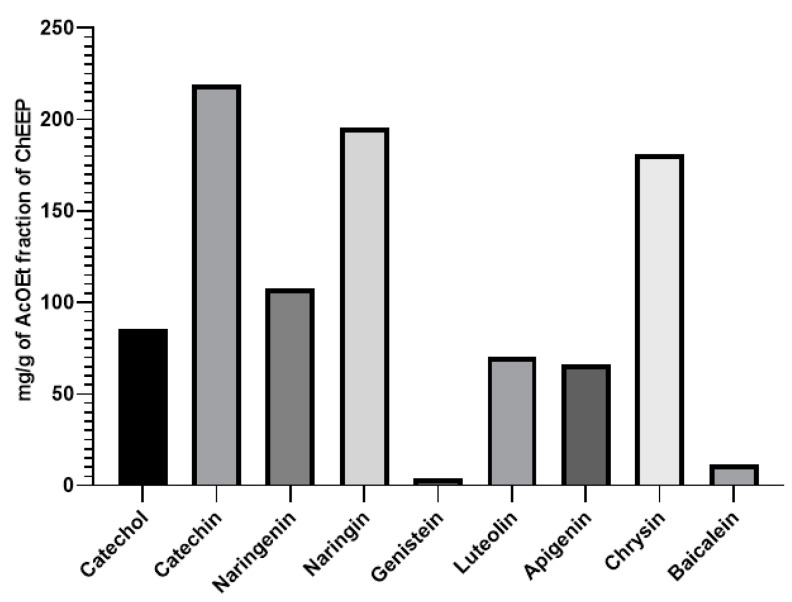
Total abundance of fractionated ChEEP from preparative plate.

For the HPLC—DAD analysis, 23 fractions obtained from the preparative plate were injected, and nine compounds were identified; these are shown in Table 4. Figure 2 shows a graphic representation of the abundance of each compound. Spectra and chromatograms for chemical identification of compounds are available in Figures S1–S3 of the supplementary material.

For the HPLC–MS analysis, the 23 fractions obtained from the preparative plate were analysed, and seven compounds were identified; the results are shown in Table 5. Spectra and chromatograms for chemical identification of compounds are available in Figures S1–S3 of the Supplementary Material.

### 2.2. Toxicity

In a previous study [19], the acute topical toxicity of ChEEP was evaluated at two different concentrations. At 50%, ChEEP presented signs of topical toxicity, such as erythema, oedema and peeling starting on Day 14; however, at 10%, ChEEP did not cause signs of toxicity throughout the experiment. In addition, according to the histological analysis, the histology of the skin treated with ChEEP was similar to that of the control group, suggesting that ChEEP was not toxic to the skin.

### 2.3. Closing Speed

**Figure 3:**
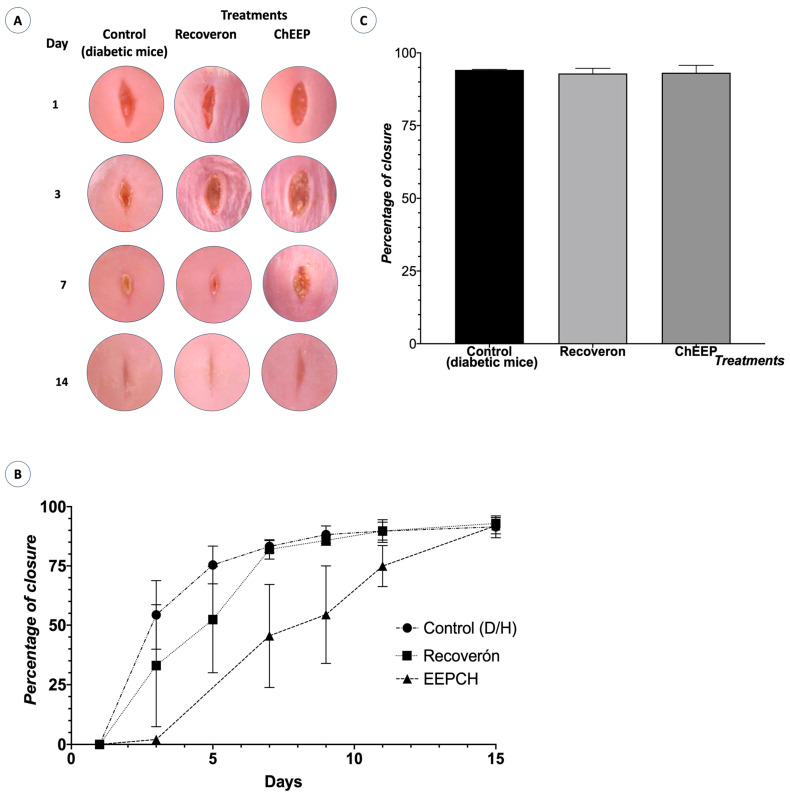
(**A**) Photographic kinetics of the wound healing (Days 1, 3, 7 and 14) (**B**) the closure speed in the experimental groups and (**C**) the closure speed in the experimental groups on Day 14, where no significant differences (*p* > 0.05) were observed (determined by one-way ANOVA).

To evaluate the effect of ChEEP on the healing process, macroscopic and histological analyses were carried out; diabetic mice were used, and a 1 cm longitudinal wound was made in the dorsal area of each mouse. The control mice (negative and positive) had faster closure during the first 7 days than the mice treated with ChEEP; however, on Day 14, the wounds in all three groups had closed completely, and the statistical analysis showed no significant differences between the groups (Figure 3).

**Figure 4:**
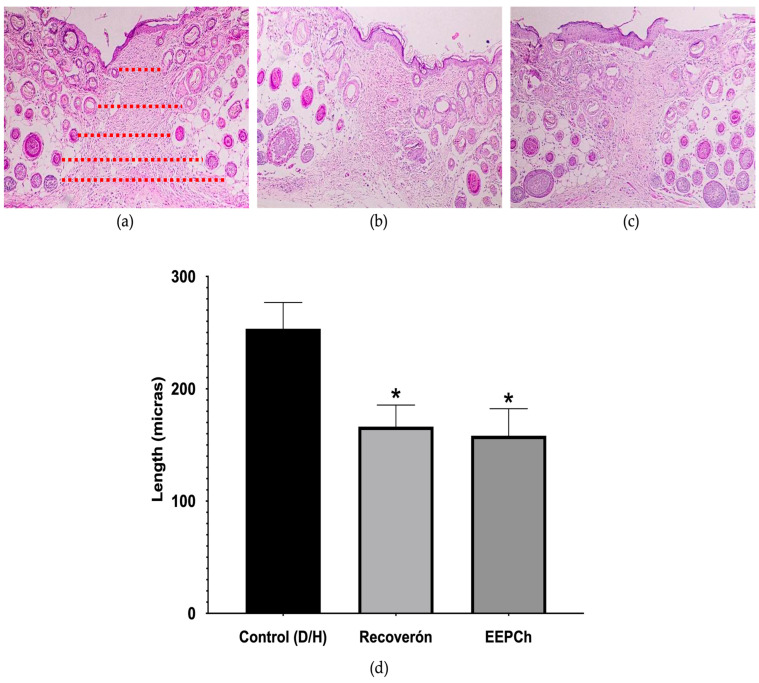
Wound skin histology and H&E (100X) staining. Representative photomicrographs of wound skin 14 days after treatment in the three experimental groups: untreated (healthy skin) (**a**), Recoveron NC (red dotted lines mark the edges of the wound. (**b**) and ChEEP (**c**); final length of wound. (**d**). * shows statistically significant differences (*p* < 0.05) between Recoveron NC and ChEEP with respect to the control group (determined by one—way ANOVA).

For the histological analysis, the width between structural cells of the skin separated by the scar area was measured. The ChEEP group and the positive control group (Recoveron NC) showed a reduction in the wound area, and a decrease in the inflammatory infiltrate was also observed. The statistical analysis revealed significant differences between the ChEEP and Recoveron NC groups and the control group (Figure 4).

### 2.4. Tensile Strength

**Figure 5:**
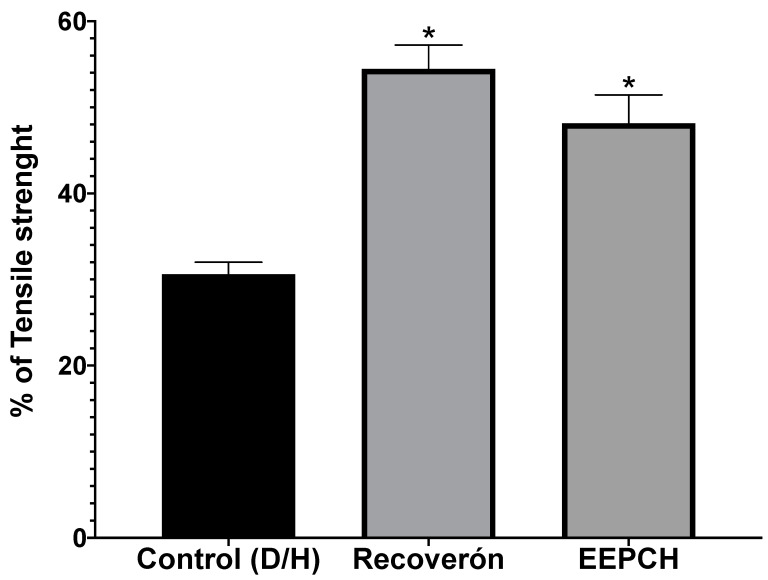
Percentage of wound tensile strength. * shows statistically significant differences (*p* < 0.05) between Recoveron NC and ChEEP with respect to the control group (determined by one—way ANOVA).

To evaluate the tensile strength of the wounds, the tensiometric method was used. This method compared the resistance of the healed tissues, which were evaluated 14 days after the wounds were made, with respect to the resistance force of healthy skin. The results showed that the use of 10% ChEEP had a healing effect of 48.15% on average, without a statistically significant difference from the use of Recoveron NC, which had a 54.45% healing force; however, with respect to the control group, both groups presented significant differences, resulting in a 30.63% increased healing effect (Figure 5).

### 2.5. Quantification of α—Actin and MMP9 by Immunohistochemical Analysis

**Figure 6:**
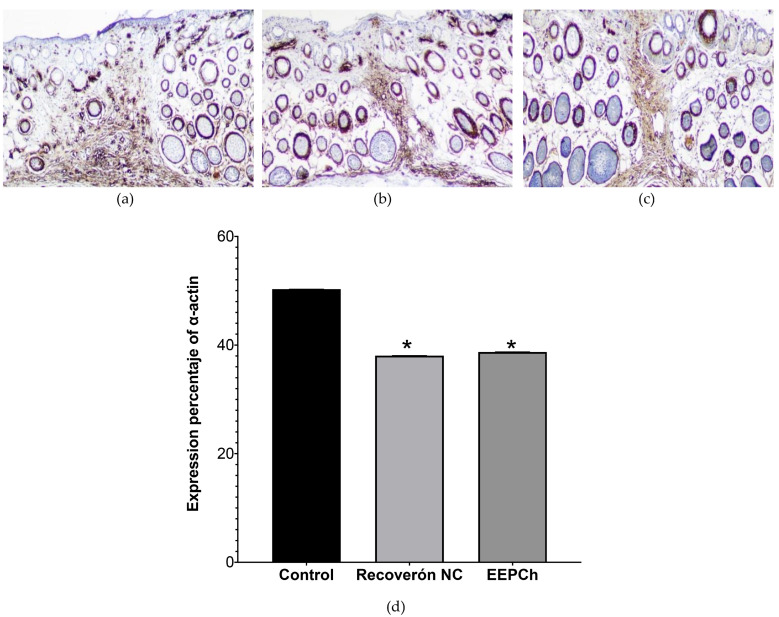
Immunohistochemical identification of smooth muscle α—actin expression (brown). Representative histological sections of the wound area on Day 14 at 100× magnification: (**a**) healthy skin, (**b**) Recoveron NC, (**c**) ChEEP and (**d**) percentage of α—smooth muscle actin expression with respect to the wound area. * shows statistically significant differences (*p* < 0.05) between Recoveron NC and propolis with respect to the control group (determined by one—way ANOVA).

Fibroblasts were identified by immunohistochemical analysis and the expression of the muscle α—actin protein. Figure 6a shows the obtained results. The brown areas are where α—actin was found at the highest concentrations (the healing tissue area). The expression of α—actin was quantified using the ImageJ program, with the healing tissue given a value of 100%; based on this, it was observed that the expression of this protein in the tissues treated with 10% ChEEP did not significantly differ from that in the group treated with Recoveron NC but did significantly differ from that in the control group (Figure 6b). This was interpreted as a delay in the healing process of the control group tissues; although complete restructuring of the epidermis was observed in the tissue, the population of fibroblasts in the area was considered indicative of immature healing tissue.

**Figure 7:**
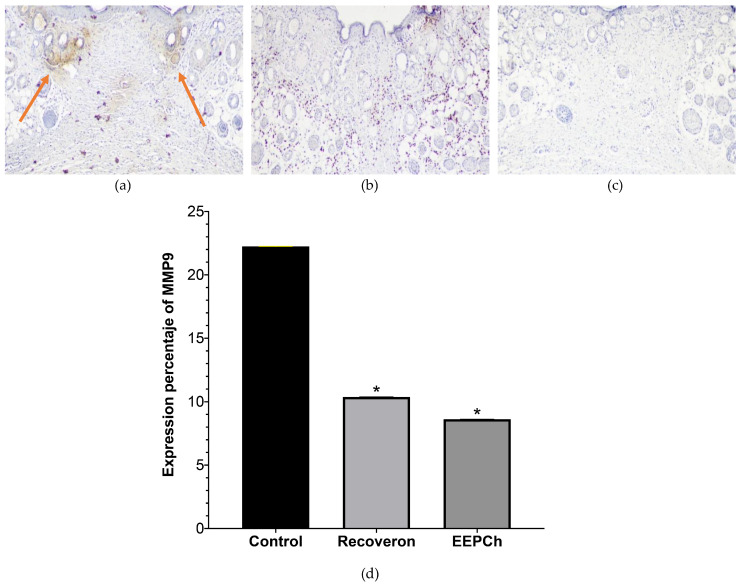
Immunohistochemical identification of MMP9 (brown). Representative histological sections of the wound area on Day 14 at 100X magnification: (**a**) healthy skin, The arrows indicate the MMP9 expression site. (**b**) Recoveron NC, (**c**) ChEEP and (**d**) percentage of MMP9 expression with respect to the wound area. * shows statistically significant differences (*p* < 0.05) between Recoveron NC and propolis with respect to the control group (determined by one—way ANOVA).

Additionally, quantification of the expression of the MMP9 enzyme was performed by immunohistochemical analysis, which revealed the areas with the highest expression (brown) (Figure 7a). Similar to the previous results, there were significant differences between the control group and the 10% ChEEP and Recoveron NC groups but not between the last two groups. A higher expression of this enzyme is an indication that the extracellular matrix is constantly being degraded because the type I collagen that usually occurs in mature healing tissue is not being produced.

## 3. Discussion

Healing is the natural process that occurs in humans after they are wounded. This process is composed of various phases, from inflammation to remodelling [19], and normally results in an effective and visible scar; however, there are different factors that can interfere with this process, such as age, genetics, immunodeficiencies, poor nutrition, smoking and chronic degenerative diseases, such as DM [4,6].

During the first phases of healing, it is normal to find ROS, which act as a defensive barrier against microbial infections and are an important part of cell recruitment, mainly that of macrophages and neutrophils, which in turn maintain the oxidative effects of these molecules, preventing them from damaging other cells. However, one of the main causes of DM related deterioration in the healing process is oxidative stress. This process occurs when oxidizing agents exceed the capacity of the endogenous antioxidant activity. In addition, oxidative stress is considered a precursor to senescence and cell death, prolonging inflammation and cell migration. Nonetheless, the presence of exogenous antioxidant agents, such as some secondary metabolites of plants, contributes to modulating the production and destruction of ROS [19,23,24].

To determine the antioxidant properties of ChEEP, its antioxidant capacity was evaluated by the DPPH radical reduction method, which resulted in a medium antioxidant capacity (AC_50_) of 29.46 µg/mL; although this value was not comparable to that of quercetin (1.89 μg/mL), quercetin is considered a pure compound. However, Rodríguez and collaborators [25] evaluated the CA_50_ of eight propolis strains from different states of the Mexican Republic, including Cuautitlan, Edo. Mexico (86.6 µg/mL); Villa del Carbón, Edo. Mexico (52.6 µg/mL); El Oro, Edo. México (48 µg/mL); Tlapujaua, Michoacán (26 µg/mL); Tianguismanalco, Puebla (189.3 and 277.99 µg/mL from 2010 and 2012, respectively); San José Iturbide, Guanajuato (83.5 µg/mL); and Tlacotalpan, Veracruz (950.4 µg/mL). These findings indicate that the majority of these propolis samples fit within the NOM—003 SAG/GAN—2017 [22] range by having a CA_50_ less than 100 µg/mL in agreement with Al-Fatimi [26], where he mentions that if the extracts are present at concentrations below 96.6 μg/mL, they are considered to have an adequate antioxidant capacity. The antioxidant capacity of an extract is due to the total content of phenols and flavonoids because they are ideal electron donors because they stabilize and quench free radicals. Phenols and flavonoids are characteristic bioactive compounds in propolis, and their main uses and biological applications are attributed to these compounds; however, the concentrations and types of these compounds can vary substantially according to the origin of the samples. According to NOM—003—SAG/GAN—2017 [22], propolis samples must meet certain parameters. Among these parameters, propolis must have a minimum percentage of 5% total phenols. The samples used in our study exceeded this requirement, with 32.53% total phenols; however, for flavonoids, the minimum required concentration is 0.5%, and our ChEEP exhibited a concentration of 5.68% total flavonoids.

Fractionation of ChEEP was performed to detect compounds based on their polarity. Twenty-three fractions were obtained and were subjected to HPLC—DAD and HPLC–MS. These 23 fractions included compounds such as catechol, catechin, naringin, naringenin, genistein, luteolin, apigenin pinocembrin, chrysin, kaempferol, acacetin and baicalein, which are consistent with the phenolic compounds reported in Mexican propolis from Chihuahua and Sonora by Bankova, Yañez and collaborators and Provencio and collaborators [27,28,29]. Phenols are the secondary metabolites most commonly used to treat skin wounds [30] due to the presence of hydroxyl groups (OH) groups within their chemical structure, especially at positions 5, 7, 3 and 4. These characteristics confer biomedical activities such as antimicrobial, antioxidant and anti-inflammatory effects [31]. During the healing process, flavonoids stimulate an increase in epithelialization rates; modulate inflammatory cytokines; accelerate wound contraction; and promote angiogenesis [32]. For example, naringenin has been shown to have anti-inflammatory and antioxidant effects since it reduces the levels of TNF—α, IL—6, NO, IL—1β, PGE2, caspase—3, LTB4 and NF—κB; restores the levels of TBARS and GSH; increases the levels of GST, GPx, SOD and CAT; and leads to more effective wound closure in a murine healing model [33,34]. Naringin promotes wound contraction in rats since it accelerates collagen synthesis and is also an anti—inflammatory agent that regulates proinflammatory cytokines and growth factors [35,36]. Luteolin accelerates wound closure in diabetic patients and decreases the inflammation induced by NF—Kβ and the oxidative response of Nrf2 [37]. Chrysin regulates the expression of MMP, tissue inhibitors of metalloproteinases (TIMPs) and IL—6 and is therefore considered an anti-inflammatory agent [38]. Kaemferol accelerates the speed of closure of incision and excision wounds and has anti—inflammatory and antioxidant properties [39,40]. Genistein and catechin reduce oxidative stress and modulate the expression of proinflammatory cytokines in the early phases of healing [41]. Pinocembrin, which has anti—inflammatory activity, is also effective and has even been reported to be proliferative in human keratinocytes [42].

For the second part of the chemical characterization, the hexanic and EA fractions and the derivatization product were subjected to a GC–MS analysis, resulting in the detection of 24 compounds classified into the following chemical groups: alcohols (1), sesquiterpenes (1), saturated fatty acids (7), hydrocarbons (8), ketones (1), chalcones (1), monosaccharides (3) and unclassified compounds (2) [43,44]. In propolis samples from different countries and regions of the world, the presence of benzoic acid, pinostrobin chalcone, mannose, palmitic acid, stearic acid, technocrisin, behenic acid and cerotic acid was confirmed by derivatization and GC–MS analysis, which is consistent with the results of this research [44,45,46].

The use of unsaturated fatty acids has been reported in the treatment of skin wounds, mainly in the inflammatory phase [47]. Seven fatty acids were detected in ChEEP, and stearic acid was highlighted. Mixing stearic acid with menthol at a proportion of 8:1 at 75 mM did not cause cytotoxicity or favour the healing process, as evaluated by the migration of HaCaT cells [48]. Additionally, palmitic acid and eicosane (hydrocarbons present in ChEEP) have been shown to regulate the expression of proinflammatory cytokines, such as TNF—α and IL—12; nonetheless, these substances have been reported to not improve fibroblast proliferation and migration and therefore the synthesis of collagen [49]. Additionally, monosaccharides such as fructose, glucose and mannose are common compounds in propolis samples [50].

One of the main parameters for using a natural product as a healing agent is a low topical toxicity; however, the toxicity of propolis has already been evaluated, and the medium lethal dose LD_50_ has been reported to range from 2600 mg/kg to 7000 mg/kg of weight [51], which agrees with the findings of Balderas et al. who reported the safety of 10% ChEEP since it did not present signs of toxicity on the 14th day [19].

In previous studies, the healing activity of Chihuahua propolis has been evaluated in healthy mice, and favourable results have been obtained [19]; however, diabetic individuals that suffer a wound can be affected by a series of complicated processes, which can delay proper healing, mainly through an increase in MMP9 levels, which leads to exacerbated inflammation and poor repositioning of α—actin (the main component of myofibroblasts). Furthermore, wound closure is generated by re—epithelialization and not by contraction in diabetic individuals, which can become a problem since closure is generated from the epidermis to the hypodermis and although it results in an aesthetic scar, it is not considered very functional; it has a very high probability of reopening with little tensile strength and becoming a difficult closure wound [19,52].

The evaluation of closure speed showed that ChEEP has similar activity to that of Recoveron NC (93.17% and 92.92%, respectively); these data agree with those of Balderas [19], who mentioned that ChEEP promotes faster wound healing in healthy mice. Voss [53] evaluated the closing speed of diabetic mice treated with propolis from Saudi Arabia, where the groups treated with propolis showed significant differences from the diabetic mice without treatment and showed that propolis decreased IL—1B, IL—6 and MMP9 levels almost to basal levels. On the other hand, Khadabakhshi resorted to dressings coated with propolis and showed that the combination of polyurethane with 30% propolis promoted a closure of almost 90%; thus, these propolis samples showed a similar behaviour to the ChEEP studied here [54].

The tensile strength was measured using a modified Vaisberg tensiometric technique [55], resulting in values of 48.15% (ChEEP), 54.45% (Recoveron) and 30.53% (control); these values agree with those of Kapare, who developed and evaluated a hydrogel formulation combined with the ethanolic extract of a propolis from India, and they evaluated the necessary strength required to reopen the wound; they showed that the hydrogel showed significant differences compared to all the experimental groups (198 g), the control group (171 g), vehicle group (176 g) and Cipladin group (210 g). In terms of closing speed, the behaviour of propolis was observed to be similar to that of other drugs [56]. Although there were no significant differences between ChEEP and Recoveron in terms of closing speed, significant differences in tensile strength were observed. Few studies have evaluated the tensile strength of a wound in diabetic patients; therefore, we can infer that tensile strength is linked to the deposition of collagen in the scar area and therefore to the closure of the wound because wound healing in diabetic patients involves re—epithelialization and not contraction [19,52]. This explains why, although completely closed wounds were observed in the epidermis in all groups, in the control group, there were differences in the restructuring of the dermis and hypodermis compared to the ChEEP and the Recoveron NC groups, where there was a greater presence and better distribution of cells throughout the layers of the skin. The measurements of the healing area showed that the control group had the greatest average length (253.34 microns), while the ChEEP and Recoveron groups had average lengths of 158.26 and 166.34 microns, respectively. These tensile strength findings support that a lower force was needed to reopen the skin of the control mice than was needed for that of the ChEEP and Recoveron NC groups [19,57]. Different authors mention that these characteristics may be attributed to the effects of diabetes on healing [4,58,59].

In cases of hyperglycaemia, the healing process results in abnormalities such as hypoxia; in addition, angiogenesis is insufficient, proteolytic activity increases and there is interference in cytokine signalling pathways. This deregulation in the environment alters the physiology of fibroblasts and, therefore, limits their differentiation to myofibroblasts, which become senescent and tend to undergo apoptosis. In a nonhyperglycaemic environment, myofibroblasts begin to differentiate starting in the inflammatory phase and are eliminated during the transition to the remodelling phase; however, in diabetes, alterations in the differentiation of myofibroblasts can lead to a lack of cell proliferation, insufficient deposition of the extracellular matrix and an increase in proteolytic activity, mainly due to hyperactivity of metalloproteinases and a decrease in wound contraction. It has been shown that by reducing contraction in a wound in patients with diabetes, the healing process depends on granulation and re—epithelialization; therefore, the extracellular matrix in the scar develops poor tolerance to traction [52,58,60].

It has been reported that one of the MMPs with the greatest activity in the healing process in diabetic patients is MMP9 since it is present in the last phases of healing, increases the decomposition of the extracellular matrix and leads to a decrease in the tensile strength of the scar area, preventing the deposition of collagen [14], Thus, tensile strength becomes an important point for the evaluation of wound healing treatments in diabetic patients.

The MMP9 expression was evaluated by immunohistochemistry on Day 14. The control group (22.23%), Recoveron NC group (10.38%) and the ChEEP group (8.6%) were evaluated, and there were significant differences between the Recoveron and ChEEP groups and the control group. These findings are consistent with those of Hozzein and collaborators [14]. These authors evaluated the presence of MMP9 for 15 days and reported an increase in MMP9 levels in the group of diabetic animals with values above 50 pg/mg total protein compared to that in the group treated with propolis from Saudi Arabia, which had values less than 20 pg/mg, almost reaching the levels of the group of healthy animals. Ernawati and collaborators [61] evaluated the presence of MMP9 in diabetic rats for nine days. On Day 3, all groups presented basal MMP9 values ranging from 7 to 8. On Day 9, the control group the level doubled to a value of 15.7, while the expression of MMP9 in the group treated with propolis from Indonesia decreased to 4 on Day 9, attributing these results to the presence of phenolic acids that inhibit the expression of proinflammatory cytokines.

The delayed differentiation of myofibroblasts in the inflammation phase causes these cells to remain abundant until Day 15 postinjury in diabetic rats induced by streptozotocin, which, as Retamal concludes, is a characteristic behaviour of diabetic wounds [60,62]. Based on this evidence and with respect to the results obtained for the identification of myofibroblasts in this work, we can observe that the application of ChEEP or Recoveron NC significantly reduced the persistence of myofibroblasts in the wound area compared with that in the control group, which presented a decreased expression of α—actin at 14 days postinjury. These results agree with those of Amrita and collaborators, who evaluated Jamun honey in a healing model in diabetic animals. The honey group showed significant differences from the diabetic group, with decreased expression of α—actin. Moreover, the number of myofibroblasts must decrease after the wound is re-epithelialized, which is why the controlled formation, existence and apoptotic reduction of additional myofibroblasts are essential for the normal wound healing process [63].

ChEEP has a varied chemical composition, with 37 identified compounds that are known to have biomedical activities and are useful for the healing process, such as antimicrobial, antioxidant, and anti-inflammatory effects, as well as some proliferative effects.

**Figure 8:**
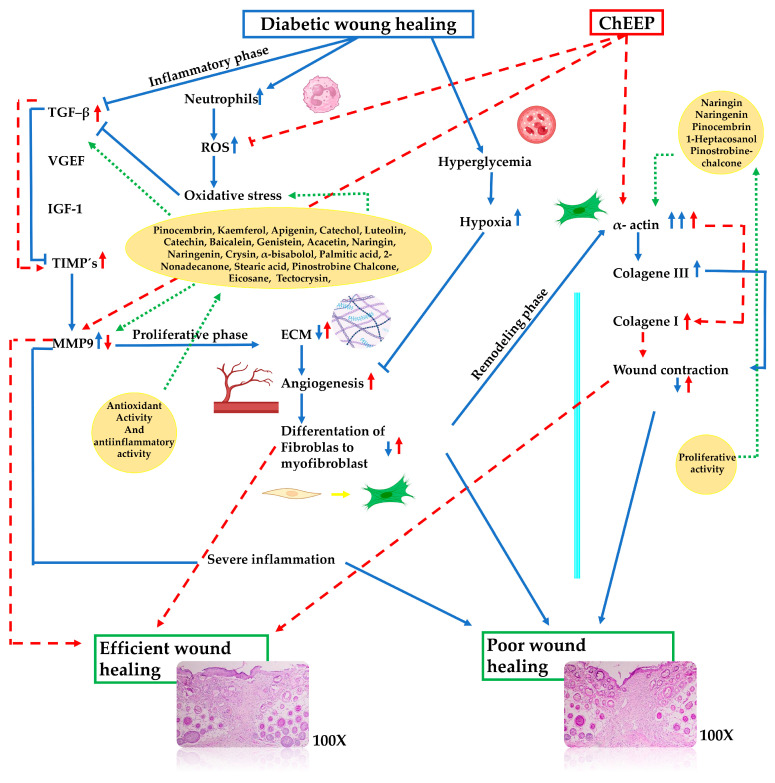
Possible mechanism of action of ChEEP in the healing process affected by DM.

As shown in Figure 8, ChEEP could affect TGF—β signalling by acting as an anti-inflammatory agent through the regulation of inflammation, preventing exacerbated inflammation [13,14]. TGF—β is a growth factor that intervenes in the healing stage, so the use of ChEEP results in a cascade of benefits beginning in the first phase of healing by stimulating the production of TGF—β, increasing the anti-inflammatory and regulatory effects of this cytokine. The production of TIMPs and, therefore, the expression of MMP9 remain regulated; in turn, excessive degradation of the ECM is avoided, and collagen deposition is promoted by TGF—β. On the other hand, the regulation of inflammation provides the necessary environment for the differentiation of fibroblasts to myofibroblasts to be accelerated, thus leading to greater contraction in the wound.

Every ChEEP fraction demonstrated antioxidant activity due to phenolic compounds [64,65,66,67,68,69,70,71,72,73,74,75,76], which can reduce ROS levels and therefore prevent oxidative stress in the scar area, generating a microenvironment favourable for healing.

The antimicrobial activity attributed to the compounds present in ChEEP [77,78,79] has an important role in healing, preventing wound infection, and promoting more effective and complication-free closure.

ChEEP could be a great candidate wound healing agent because it has antimicrobial, antioxidant and anti—inflammatory properties and promotes a controlled environment in each stage of the healing process even if this process is affected by DM.

## 4. Materials and Methods

### 4.1. Chemicals and Solvents

Hexane, ethyl acetate, methanol (HPLC grade), gallic acid, DPPH (2,2-diphenyl-1-picrylhydrazyl), benzene, acetone, dichloromethane were obtained from Sigma-Aldrich (St. Louis, MO, USA). Folin–Ciocalteu reagent was obtained from HYCEL (Zapopan, Mexico). The HPLC database standards were kaempferol, catechin, pinocembrin, baicalein, naringenine, naringin, catechol, quercetin, luteolin, genistein, caffein, apigenin, myricetin, chrysin, acacetin and were purchased from Sigma-Aldrich; the hydrocarbon standards 1-octene and octadecane for GS-MS and streptozotocin were obtained from Sigma-Aldrich.

### 4.2. Propolis

The propolis sample was collected by the engineer Martín Balcorta Baeza in 2013 from the apiary located in the Ejido Concordia (28°41′1″ N and 106°0′8″ W) in the municipality of Aquiles Serdán (Chihuahua, Mexico). ChEEP was obtained by the maceration method.

### 4.3. Organoleptic Properties

The organoleptic properties of ChEEP were evaluated according to NOM-003-SAG/GAN; the propolis sample was tested, analysed and described in a general manner according to [22].

### 4.4. Antioxidant Capacity

The extracts and quercetin at concentrations ranging from 1 to 1000 ppm were placed in test tubes; subsequently, 50 μL of the different concentrations were placed in a 96-well plate, 150 μL of DPPH was added, and the mixture was incubated for 30 min at 37 °C in the dark for determination via an ELISA reader at 540 nm [80].

### 4.5. Total Phenols

A standard curve was prepared with gallic acid (0.2 mg/mL), from which, serial aliquots were taken and brought to a total volume of 1 mL with distilled water. One millilitre of solution of each of the various concentrations (gallic acid and ChEEP) was taken and placed in a test tube with 7 mL of distilled water; subsequently, 500 μL of Folin–Ciocalteu reagent was added, and after five minutes, 1.5 mL of a solution of Na_2_CO_3_ (200 g/L) was added. After two hours of reaction at room temperature, the absorbance at 760 nm was determined and the results are expressed in equivalents of gallic acid/g propolis [81].

### 4.6. Total Flavonoids

A quercetin standard curve (0–100 mg/L) was prepared, and 1 mL of 2% aluminium chloride (AlCl_3_) in MeOH was prepared and mixed with 1 mL of the ChEEP stock solution. The test was performed in 96-well ELISA plates. After reacting for 10 min at room temperature, the absorbance at 415 nm was determined. The results are expressed as equivalents of quercetin/g ChEEP [82].

### 4.7. Fractionation of ChEEP

Fractionation of the ChEEP solution was carried out using a polarity gradient; 1 g of the ChEEP was weighed, and hexane was added (yield 23.1 mg, 2.31%). Then, EA was added to the residue (yield 392.3 mg, 39.23%), after which, methanol was added to the residue (584.6 mg, 58.46%).

### 4.8. Preparatory Plate

A mobile phase of benzene–acetone–dichloromethane (3:1.5:0.5) was used. Subsequently, 100 mg of the EA fraction was seeded in a 20 × 20 cm and 2 mm thick silica gel Prep-TLC plate on a support of glass with fluorescence indicator F254 from the Radocomercial Company. Chromatographic separation was conducted three times until differentiated bands were obtained, and each band was scraped off the silica plate and labelled before processing by filtration to obtain each of the fractions [83].

### 4.9. Antioxidant Capacity Determination by Thin Layer Chromatography (TLC)

For the 23 fractions obtained through preparative plate chromatography, the measurement of their antioxidant capacity was carried out using the TLC bioautography method, which consisted of placing 10 µL of each fraction and the standards (caffeic acid, quercetin, naringenin and methanol; Sigma-Aldrich, St. Louis, MO, USA) on the previously segmented plate to be sprayed with DPPH, and the in situ colourimetric reaction of the fractions with DPPH was evaluated. This technique results in a pale yellow colour in stains that contain compounds with antioxidant activity [84,85].

### 4.10. GC–MS

For the silylation derivatization method, 5 mg of extract was weighed into a glass tube and mixed with 50 µL of pyridine plus 75 µL of bis—(trimethylsilyl) trifluoracetamide (BSTFA). The mixture was sealed and heated at 100 °C for 60 min. Subsequently, the residue of the solvent was evaporated and dissolved in 500 µL of hexane. One microlitre of the final solution was injected into the GC–MS instrument. For the analysis, a gas chromatograph 6850 network GC system (Agilent Technologies, Santa Clara, CA, USA) was coupled to a Model 5975 C mass spectrometer equipped (Agilent Technologies) with an HP-5MS column that was 30 m in length, 0.25 mm in internal diameter and 0.25 µm in thickness (Agilent Technologies). The programmed temperature was varied from 100 to 300 °C with an increase rate of 5 °C/min, and helium was used as the carrier gas with a run flow rate of 0.7 mL/min. Injection was conducted in split mode with a split ratio of 1:20 and an injector temperature of 280 °C. The range of detected masses was *m*/*z* 35–600 with an ionization voltage of 70 eV and an interface temperature of 300 °C. The total running time was 40 min. 1—Octene and octadecane (Sigma—Aldrich; St. Louis, MO, USA) were injected as hydrocarbon standards [86].

### 4.11. HPLC—DAD

For the HPLC–DAD analysis, a Hewlett—Packard HP series 1100 (Hewlett—Packard, Wilmington, DE, USA) instrument operated with ChemStation software v. A.09.03 and a Discovery C18 column (250 × 4.6 mm, particle size of 5 µm) was used. The sample was eluted with an isocratic mixture of methanol/acetonitrile/water (25:25:50) with a flow rate of 1 mL/min and measured at a wavelength of 260 nm with a complete screen from 200 to 400 nm. Thirty microlitres of a stock solution of 3 mg/mL of extract in HPLC—grade MeOH was injected into the instrument. Additionally, 30 µL of 15 flavonoid standards (vanillin, quercetin, catechin, luteolin, pinocembrin, chrysin, baicalein, myricetin, naringenin, naringin, gallic acid, catechol, apigenin, acacetin and kaempferol; Sigma—Aldrich, St. Louis, MO, USA) were injected at a concentration of 1 mg/mL.

### 4.12. HPLC–MS

An Agilent 1200 infinity LC liquid chromatograph coupled to an Agilent 6230 TOF time—of—flight mass spectrometer was used with a dual electrospray ionization (ESI) source (ESI 5614289023) and MassHunter Workstation software, v. B.05.01, build 5.01.5125.3, and data were obtained in negative ionization mode. The capillary voltage was 3500 V, the dry gas temperature was 250–300 °C, and nitrogen was used as the dry gas at a flow rate of 6 L/min. The nebulizer pressure was 60 psi, the fragmentation voltage was 200 V, and the mass range was *m/z* 50–1000. The mass acquisition rate was 1 spectrum/s. Chromatographic separation was performed using an HPLC (Infinity Series 1200, Agilent Technologist, Waldbronn, Germany) instrument equipped with a Kinetex C—18 column (150 m × 2.1 mm, 2.6 µ particle size, 100 A pore size; Phenomenex, Torrance, CA, USA). A gradient elution programme was applied with mobile phase A (water with 1% formic acid) and mobile phase B (pure acetonitrile). The flow rate was 2 mL/min at a constant pressure of 600 bar. The elution gradient programme started with 80% A and 20% B, and after 10 min, it was changed to 70% A and 30% B. At 30 min, it was modified to 60% A and 40% B, which was held until 50 min. Subsequently, at 65 min, the proportions were modified to 30% A and 70% B; finally, from 70 min to 80 min, proportions of 0% A and 100% B were maintained. The total running time was 80 min.

### 4.13. Laboratory Animals

Thirty male CD—1 mice (*Mus musculus*, 4 to 6 weeks old) were used. The animals were housed in a ventilated room at 23 °C under a light–dark cycle in polycarbonate cages with food and water available ad libitum. The present study protocols were approved by the Comité Institucional de Ética from Facultad de Estudios Superiores Iztacala (FESI), Universidad Nacional Autónoma de México (UNAM), according to the guidelines of the Reglamento Federal para la Experimentación y Cuidado Animal (NOM—062—ZOO—1999), Secretaría de Agricultura, Ciudad de México. For the closure speed assessment, fifteen mice were randomly divided into three groups, each consisting of 5 mice. Similarly, for the tensiometric method, 15 mice were randomly divided into three groups, each comprising 5 mice.

### 4.14. DM Induction

Thirty male CD—1 mice aged 4 to 6 weeks were used. Experimental diabetes was induced by administering a dose of 130 mg/kg streptozotocin (STZ, dissolved in citrate buffer (0.05 M) at a pH of 4.5) intraperitoneally. Subsequently, a puncture was made in the tail vein to measure the glucose concentration via an AC-CU-CHEK Instant Roche glucometer (Basel, Switzerland) for 7 days after the administration of STZ. The animals that presented blood glucose concentrations greater than 250 mg/dL in the fasting state were considered diabetic and were included in subsequent experiments [87].

### 4.15. Healing Efficiency

#### 4.15.1. Closing Speed

Twenty—four hours before the test, the dorsal trunk area of the diabetic mice was shaved and depilated. The mice were anaesthetized with 250 µL of isoflurane, and 1 cm long incisions were made through the skin and subcutaneous tissue. The animals were randomly distributed, and three experimental groups were established: a negative control group of diabetic animals with wounds but with no treatment; a positive control group of diabetic animals treated with Recoveron NC^®^ (sodium acexamate 5 g, neomycin sulphate equivalent to a base of 0.4 g of neomycin; excipient cbp 100 g) (Armstrong Laboratories, Mexico City, Mexico, a commercial drug for wound healing; and an experimental group treated with a 10% ChEEP formulation. For each treatment, 50 mg of each compound was applied topically to the lesion area twice daily. The incision area was photographed daily with a Celestron digital handheld microscope (Torrance, CA, USA) to determine the area of wound contraction with the ImageJ program v. 2.14.0/1.54f. The shrinkage data are expressed as a percentage of the initial lesion area. Mice were sacrificed in a CO_2_ chamber at 14 days after treatment, and skin samples, which were used for H&E staining and immunohistochemical analysis, were obtained and processed from the injury site for histological evaluation. Photomicrographs were taken, and the histological architecture of the experimental groups was compared. The results are expressed as the percentage of wound closure based on the difference in size between lesions in the different groups [19].

#### 4.15.2. Tensile Strength

The tensiometric method was used to measure the tensile strength of the wound. Diabetic mice were shaved, anaesthetized, and incised as described for the wound contraction assay. The same three experimental groups were established for the wound incision model: a negative control (diabetic animals with wounds but with no treatment), positive control group (Recoveron NC) and a 10% ChEEP treatment group. After 14 days of treatment, the mice were sacrificed, and the tensile strength was taken as the load in grams necessary to reopen the wound. The results are expressed as a percentage of the tensile strength [19,55].

### 4.16. Immunohistochemical Analysis

For immunohistochemical analysis, the rabbit-specific HRP/DAB (ABC) Detection IHC Kit ab64261 Abcam (Cambridge, UK) protocol was followed, using the primary antibodies ab124964 Abcam (Cambridge, UK) kit for α-actin determination and ab228402 Abcam (Cambridge, UK) for MMP9 determination.

### 4.17. Statistical Analysis

All healing efficiency, speed, force, H&E and immunohistochemical data are expressed as the means ± SDs. Statistical differences between the treatment and control groups were tested by one-way analysis of variance (ANOVA) using GraphPad Prism 9 software v. 9.0.0, and a significant difference was considered to be represented by a value of *p* < 0.05.

## 5. Conclusions

The present study’s findings underscore the potential of Chihuahuan propolis (ChEEP) as a promising agent to promote adequate wound healing, particularly in diabetic conditions. The comprehensive analysis of ChEEP’s chemical composition and antioxidant, anti—inflammatory, and antimicrobial properties provides valuable insights into its therapeutic efficacy.

This study’s exploration of the impact of ChEEP on wound closure, tensile strength and expression of different molecules such as α—actin and MMP9 in diabetic mice reveals promising results, suggesting its potential as an effective treatment modality for wound healing by mitigating the detrimental effects of diabetes on the wound-healing process.

Looking ahead, further research is warranted to elucidate the mechanisms by which ChEEP exerts its curative effects and to optimize its formulation for clinical applications. Future studies should also explore the long-term efficacy and safety profile of ChEEP in diverse patient populations, including those with chronic wounds and other comorbidities.

## Figures and Tables

**Table 1:** Organoleptic properties of ChEEP.

Organoleptic Properties				
	Colour	Odour	Flavour	Consistency
ChEEP	Dark yellow	Waxy balsamic	Slightly spicy	Rigid

**Table 2:** General chemical characterization of ChEEP.

General Chemical Characterization					
	Propolis	Yield	Medium Antioxidant Capacity	Total Phenol Content	Total Flavonoid Content
ChEEP	300 g	67%	29.46 µg/mL	32.53%	5.68%

**Table 3:** List of compounds identified via GC–MS.

Compound^Type of Compound/Fraction^	Retention Time	Percentage Area (%)	Similarity Percentage
α—Bisabolol^sesquiterpene/a^	15.8178	0.647	94
Palmitic acid^fatty acid/a^	20.6293	1.97	99
Hexadecanal^aldehyde/a^	22.4513	0.315	99
Heneicosane^alcane/a^	23.8755	0.072	99
cis—13—Octadecenoic acid, methyl ester^fatty acid/a^	23.946	3.098	99
2—nonadecanone^ceton/a^	24.0102	1.497	99
Stearic acid^fatty acid/a^	24.4015	0.689	99
Bicyclo[10.8.0]eicosane, cis—^hydrocarbon/a^	26.1336	1.015	97
Methyl 18—methylnonadecanoate^/a^	27.8786	1.883	99
Pinostrobine chalcone^chalcone/a^	29.2194	2.701	98
13—Docosen—1—ol, (Z)^/a^	29.5203	1.146	98
9—Nonadecene^hydrocarbon/a^	30.2138	0.572	95
Eicosane^alcane/a^	30.6051	1.132	97
Behenic acid^fatty acid/a^	31.0991	1.905	99
Tectocrysine^flavone/a^	32.2731	0.412	95
Lignoceric acid^fatty acid/a^	34.095	3.337	98
Cerotic acid^fatty acid/a^	36.8793	0.808	91
1—Nonadecene^hydrocarbon/a^	38.7397	1.836	95
1—Docosene^hydrocarbon/a^	38.8295	1.39	97
Isohexane^hydrocarbon/b^	1.5246	0.16	91
1—Heptacosanol^alcohol/b^	32.1962	7.798	95
β—d—glucose^monosacarid/c^	16.5878	0.245	91
α—D—Glucopiranose^monosacaride/c^	20.6295	3.406	94
D—manose^monosacaride/c^	21.1491	2.393	91
Oxirane, hexadecyl^hydrocarbon/c^	26.1274	0.388	95
Pinostrobine chalcone^chalcone/c, a^	29.2324	0.537	97
Lignoceric acid^fatty acid/c, a^	34.0824	0.354	90

Note: Compounds identified in GC–MS with their retention time (RT) and percentage of similarity, where “^a^” is the hexane fraction, “^b^” is the ethyl acetate fraction and “^c^” is the derivatized propolis.

**Table 4:** List of compounds identified via HPLC-DAD.

Compound^Type of Compound/Fraction^	Retention Time	Total Abundance	Match
Catechol^Phenol/1–4, 6–12, 14–16, 22–23^	2.362–2.940	85.2983	802–894
Catechin^Flavonol/1–23^	2.169–3.434	219.024	904–982
Naringin^Flavanone/2–4, 12–18, 20–23^	3.968–5.703	195.693	805–941
Naringenin^Flavanone/2–4, 11–23^	9.968–8.98	107.7774	823–979
Genistein^Isoflavone/13^	3.231	3.94	823
Luteolin^Flavone/13, 16–18^	7.749–9.889	70.53	829–923
Apigenin^Flavone/16–18^	6.921–7.789	66.2	843–889
Pinocembrin^Flavanone/18, 21^	8.529, 11.873	180.97	981, 999
Chrysin^Flavone/18, 20–22^	12.165–12.991	11.5	801–961

**Table 5:** List of compounds identified via HPLC–MS.

Compound^Type of Compound/Fraction^	Retention Time	Error (ppm)
Kaemferol^Flavonol/13, 14, 15, 17, 18 and 19^	285.0545–285.0671	−2.94, 3.68, −9.81, −8.16, −6.41 and −8.04
Pinocembrin^Flavanone/13, 14, 17 and 18^	255.0461–255.0540	−11.39, −2.96, 7.68 and 9.37
Acacetin^Flavone/13 and 14^	283.0469–283.0519	−1.79 and −3.26
Genistein^Isoflavone/15, 16 and 17^	269.0273–269.0402	4.98, −6 and 11.39
Apigenin^Flavone/15, 16 and 17^	269.0646–269.0729	−11.81, −3.05 and 12.67
Baicalein^Flavone/15, 16 and 17^	269.0595–269.0731	2.94, −1.57 and −9.98
Naringenin^Flavanon/18, 19, 20, 21, 22 and 23^	271.0385–271.0437	6.34, 8.55, 3.38, 2.59, 2.92 and −1.81

## Data Availability

The data used to support the findings of this study are available from the corresponding author upon request.

## Supplementary Materials

The following supporting information can be downloaded at: https://www.mdpi.com/article/10.3390/ijms25042201/s1.
