# Supplementary material for: Effect of Mexican Propolis on Wound Healing in a Murine Model of Diabetes Mellitus
Source: Int J Mol Sci. 2024 Feb 12;25(4):2201. doi: 10.3390/ijms25042201 (PMC10889666; doi:10.3390/ijms25042201)
Supplement: Supplementary file 1 [file ijms-25-02201-s001.zip › ijms-2855919-supplementary.pdf]

Figure S1. GC–MS chromatograms of the hexanic and EA fractions and derivatization of ChEEP.

Hexanic fraction of ChEEP.

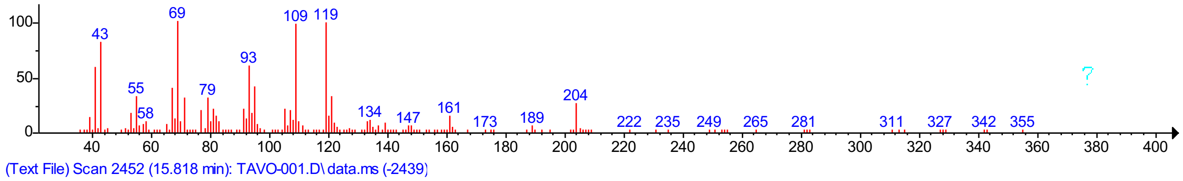

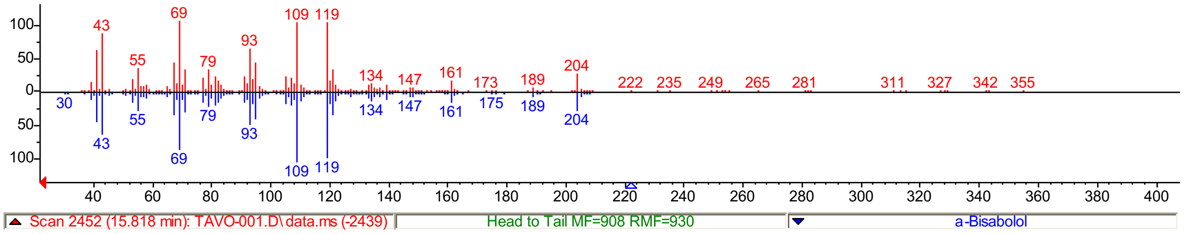

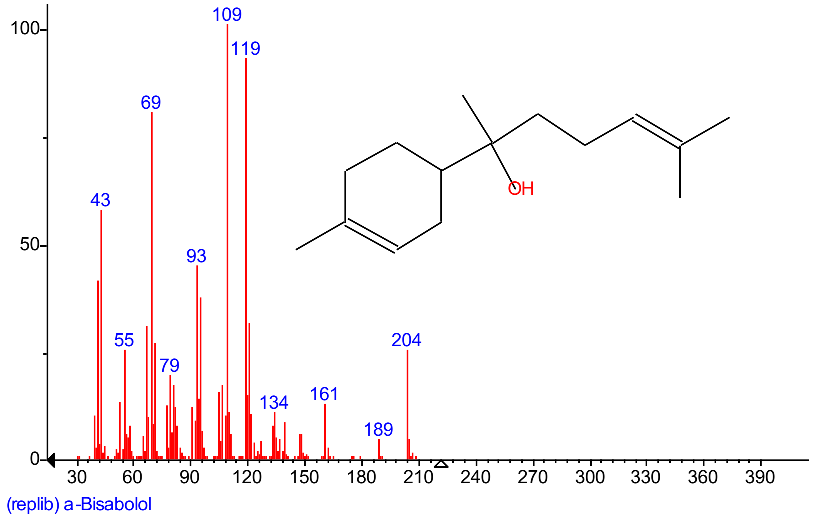

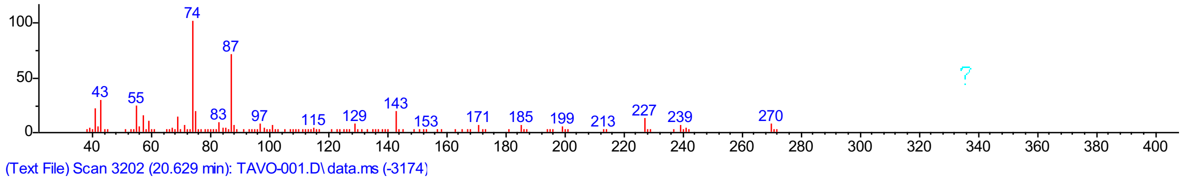

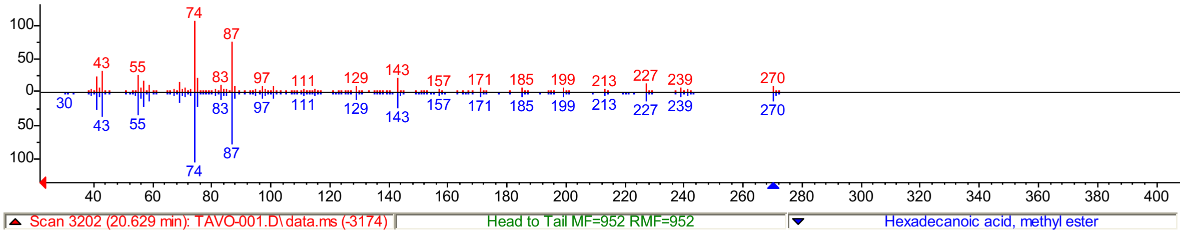

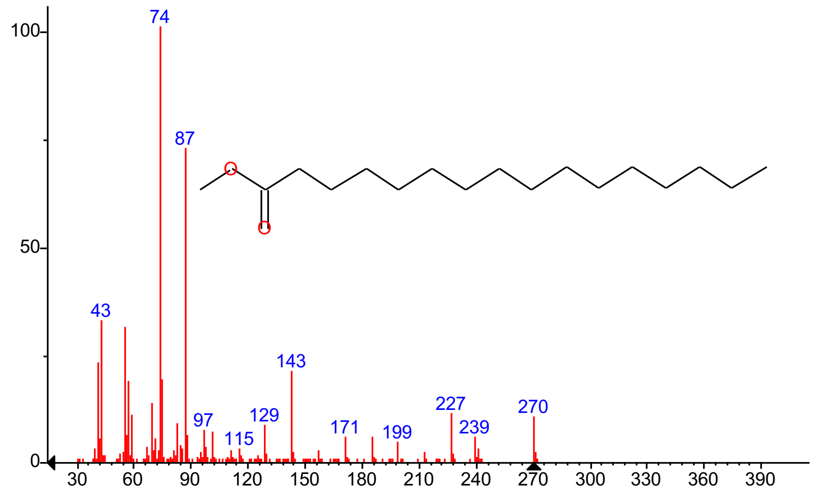

(mainlib) Hexadecanoic acid, methyl ester

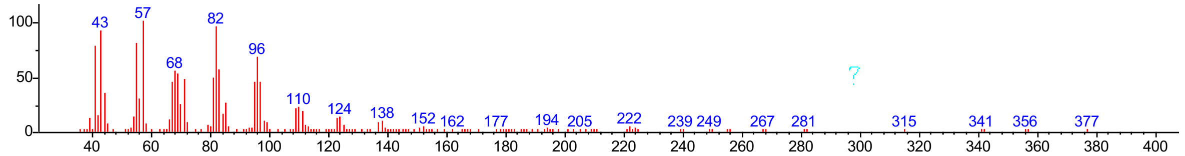

(Text File) Scan 3486 (22.451 min): TAVO-001.D\ data.ms (-3461)

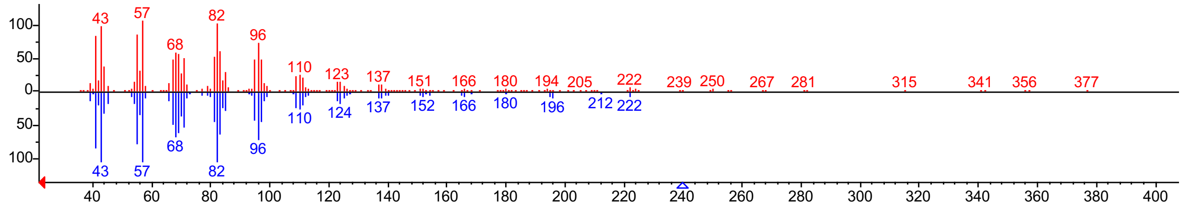

Scan 3486 (22.451 min): TAVO-001.D\ data.ms (-3461) | Head to Tail MF=888 RMF=947 | Hexadecanal

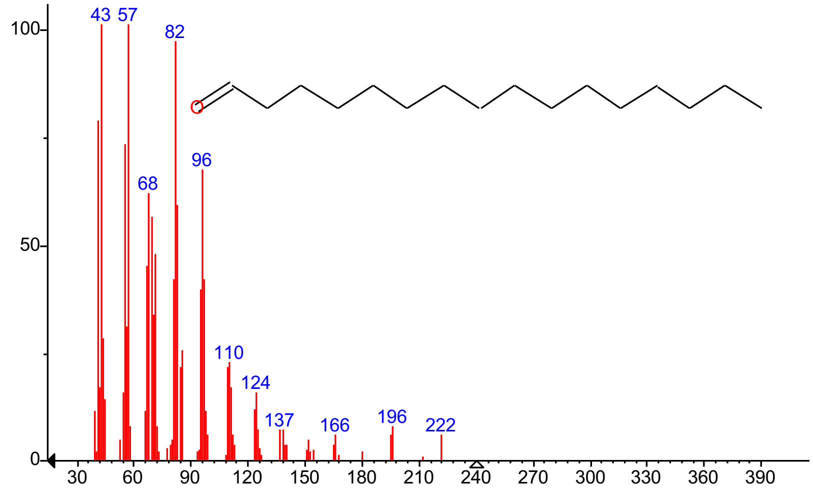

(replib) Hexadecanal

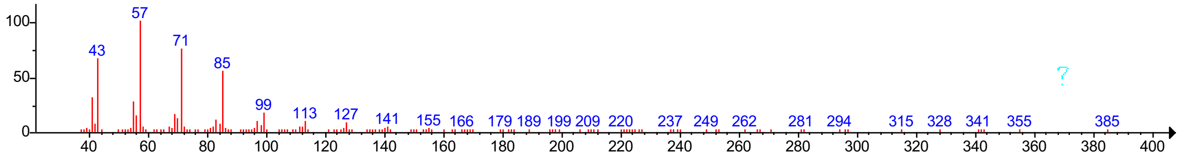

(Text File) Scan 3708 (23.875 min): TAVO-001.D\ data.ms (-3661)

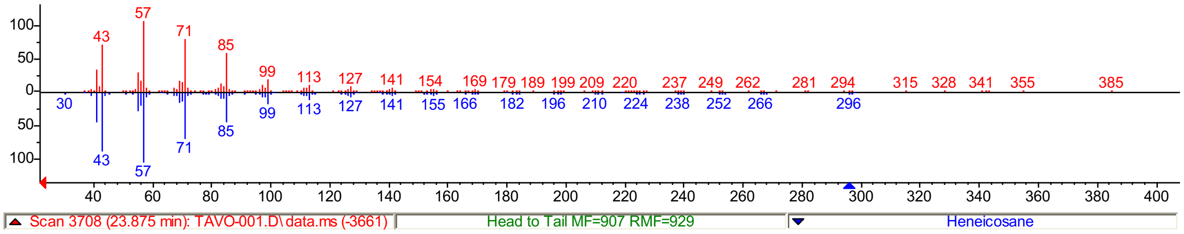

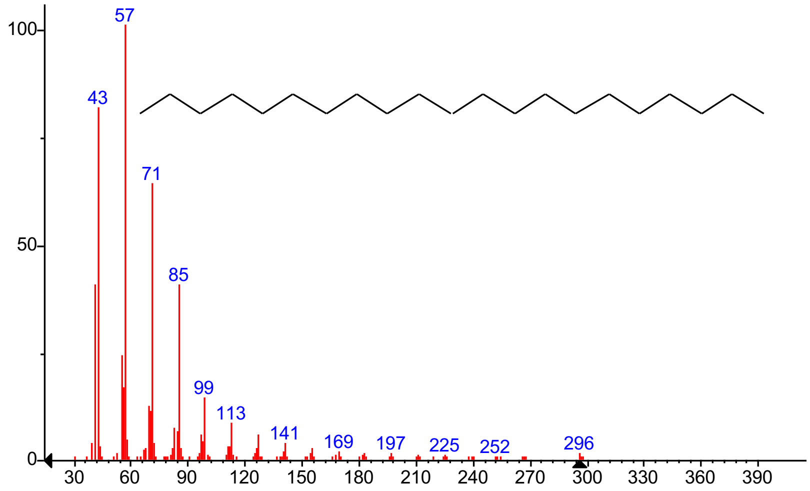

(replib) Heneicosane

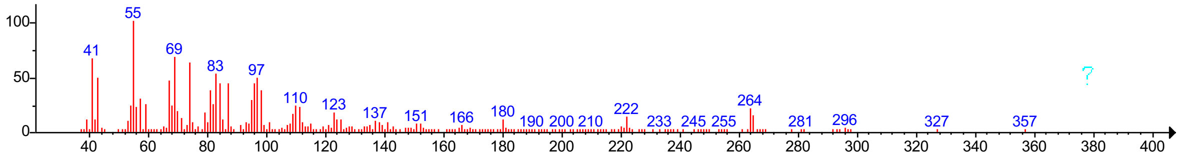

(Text File) Scan 3718 (23.940 min): TAVO-001.D\ data.ms (-3669)

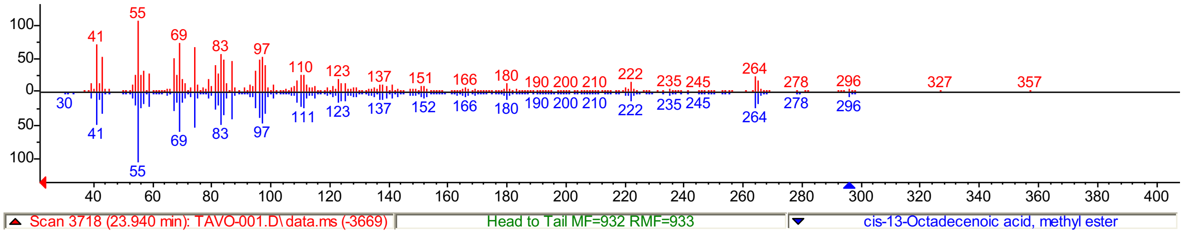

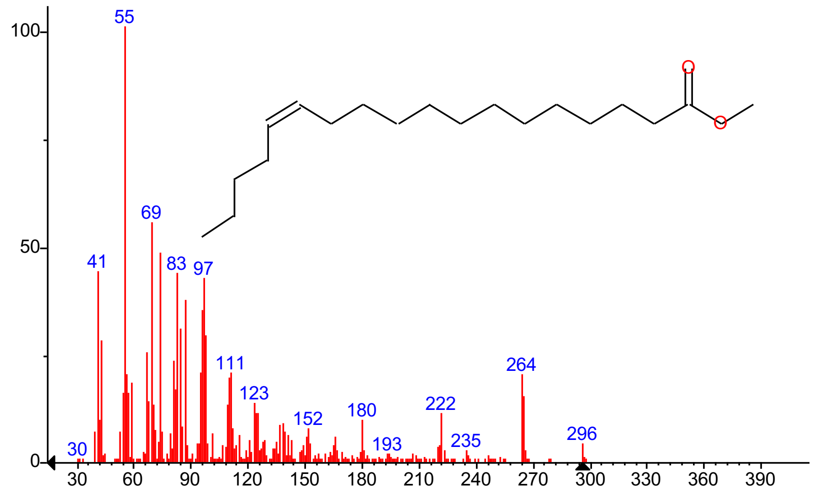

(mainlib) cis-13-Octadecenoic acid, methyl ester

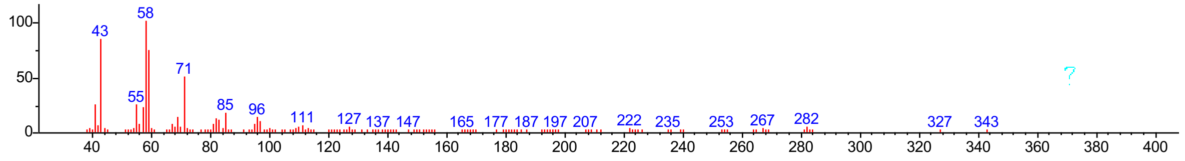

(Text File) Scan 3729 (24.010 min): TAVO-001.D\ data.ms (-3669)

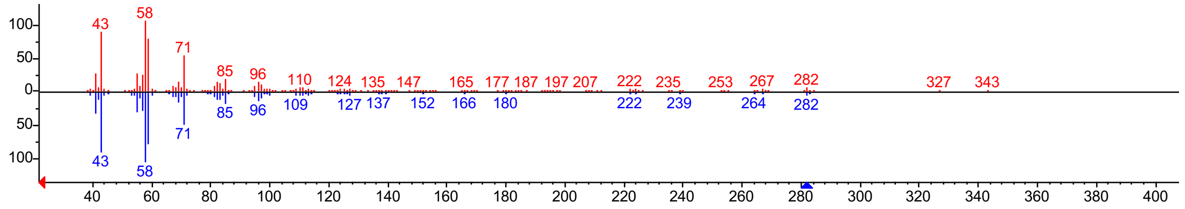

Scan 3729 (24.010 min): TAVO-001.D\ data.ms (-3669) | Head to Tail MF=924 RMF=966 | 2-Nonadecanone

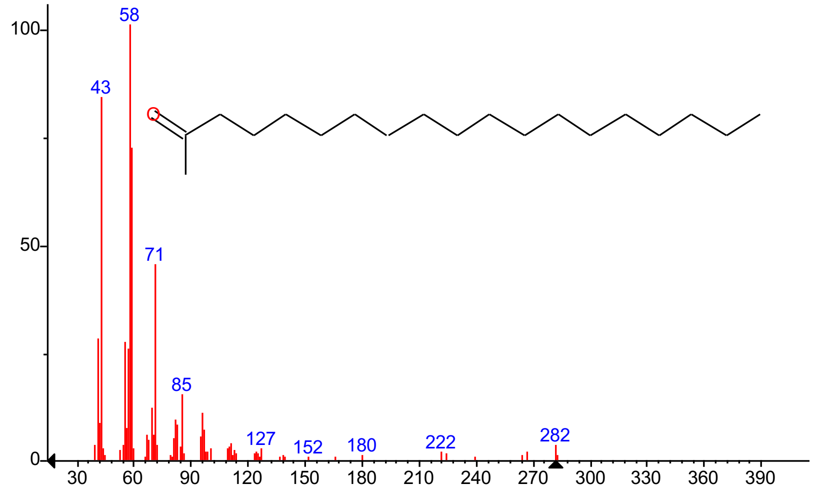

(mainlib) 2-Nonadecanone

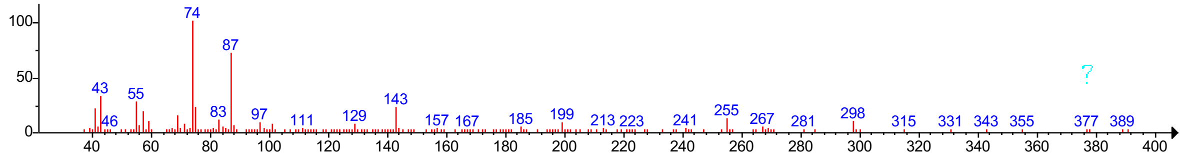

(Text File) Scan 3790 (24.402 min): TAVO-001.D\ data.ms (-3655)

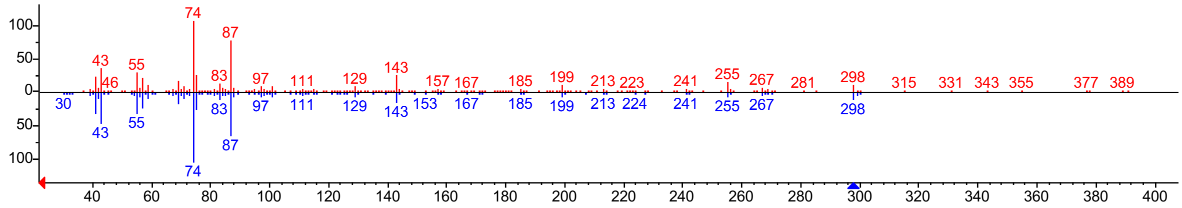

Scan 3790 (24.402 min): TAVO-001.D\ data.ms (-3655) | Head to Tail MF=917 RMF=940 | Octadecanoic acid, methyl ester

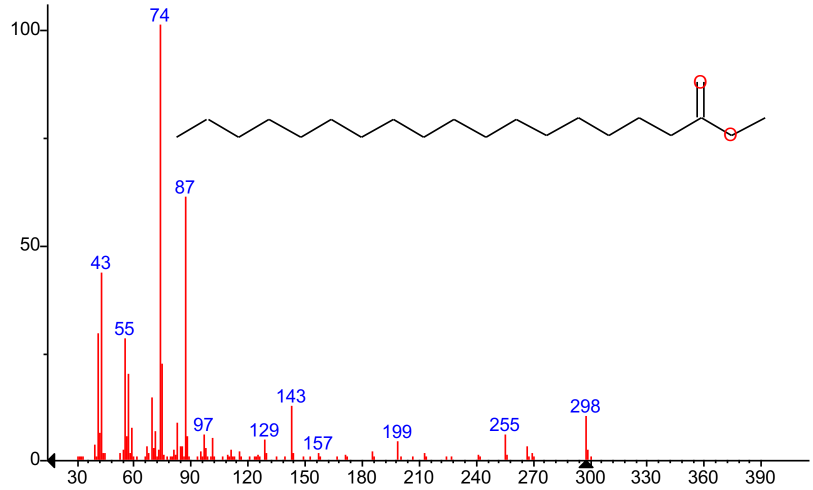

(replib) Octadecanoic acid, methyl ester

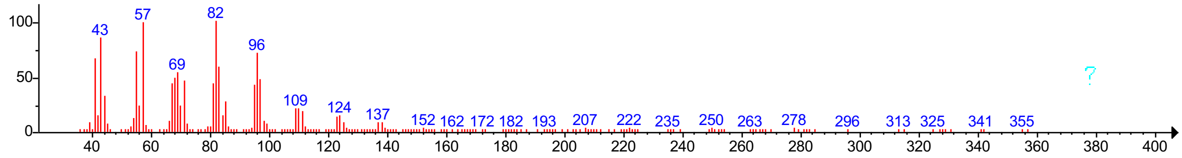

(Text File) Scan 4060 (26.134 min): TAVO-001.D\ data.ms (-3920)

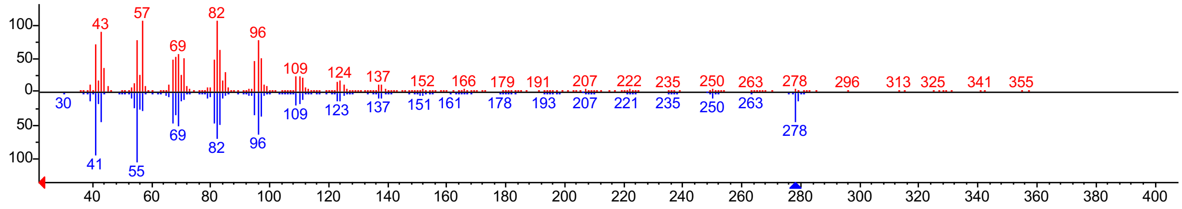

Scan 4060 (26.134 min): TAVO-001.D\ data.ms (-3920) | Head to Tail MF=794 RMF=810 | Bicyclo[10.8.0]eicosane, cis-

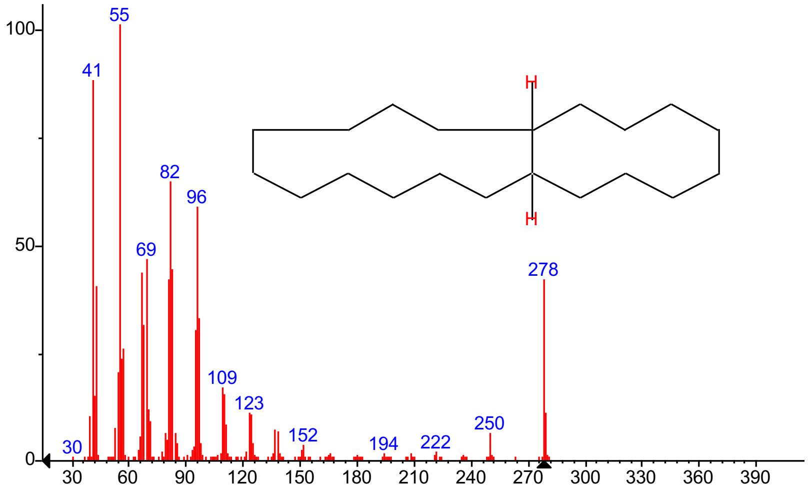

(mainlib) Bicyclo[10.8.0]eicosane, cis-

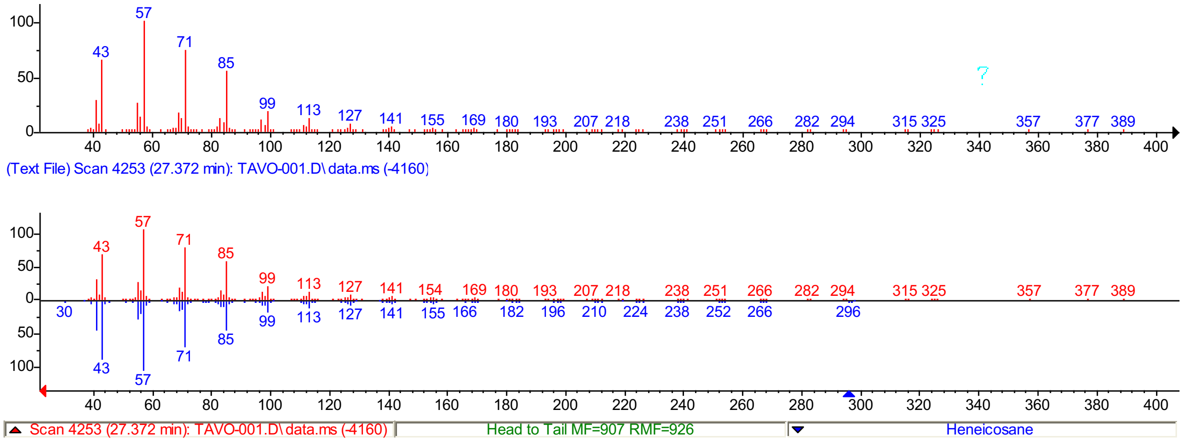

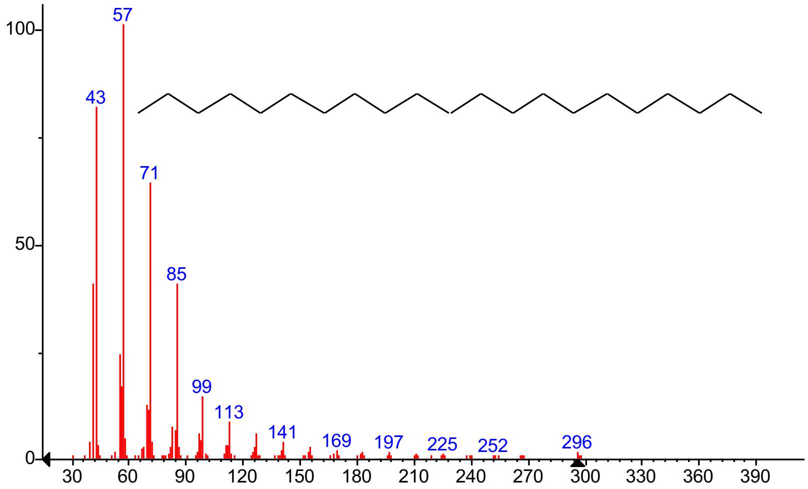

(replib) Heneicosane

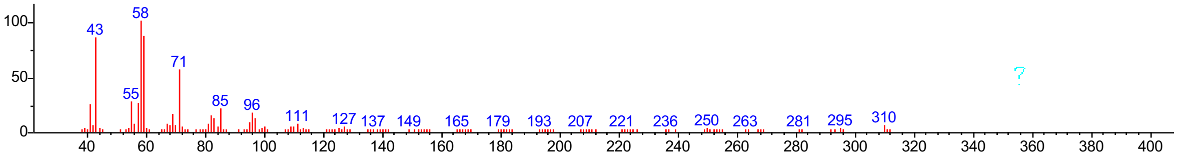

(Text File) Scan 4283 (27.564 min): TAVO-001.D\ data.ms (-4162)

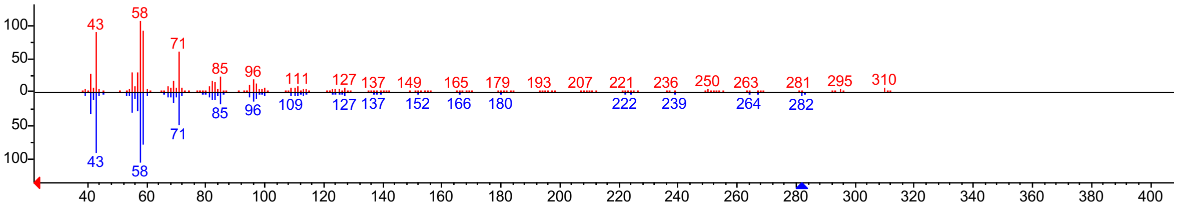

Scan 4283 (27.564 min): TAVO-001.D\ data.ms (-4162) | Head to Tail MF=837 RMF=924 | 2-Nonadecanone

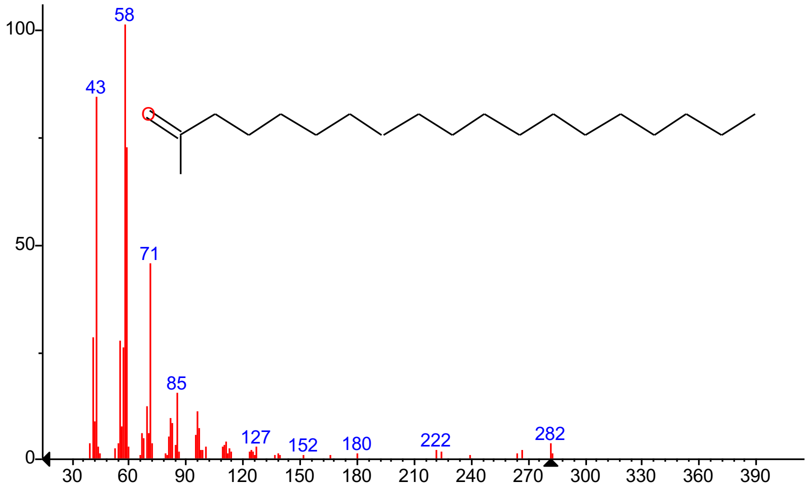

(mainlib) 2-Nonadecanone

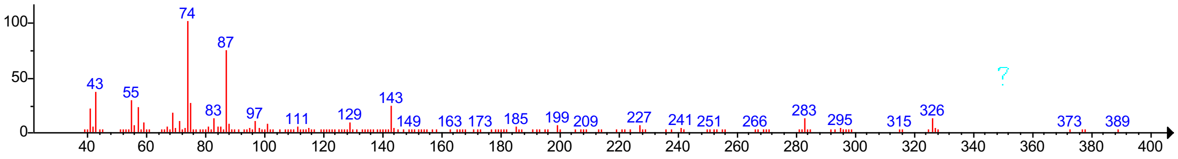

(Text File) Scan 4332 (27.879 min): TAVO-001.D\ data.ms (-4160)

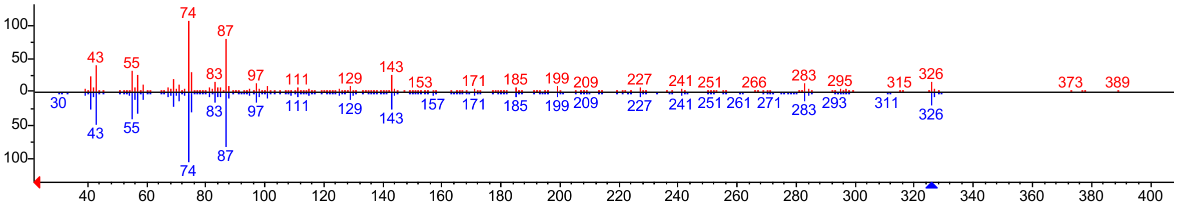

Scan 4332 (27.879 min): TAVO-001.D\ data.ms (-4160) | Head to Tail MF=897 RMF=901 | Methyl 18-methylnonadecanoate

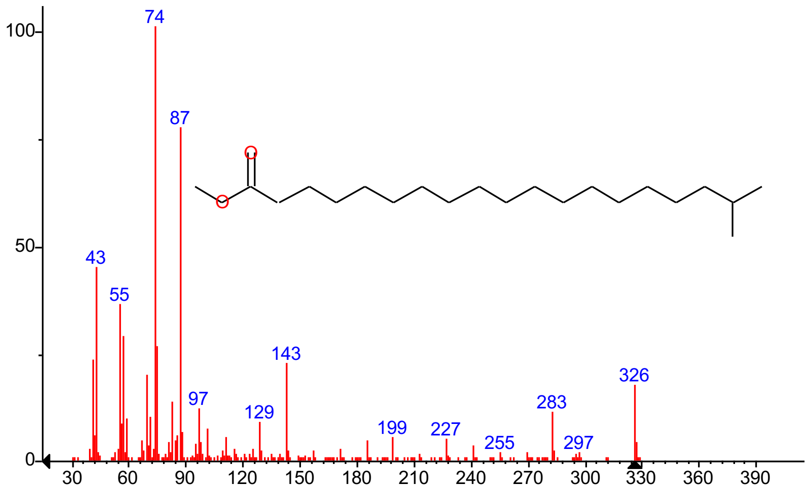

(mainlib) Methyl 18-methylnonadecanoate

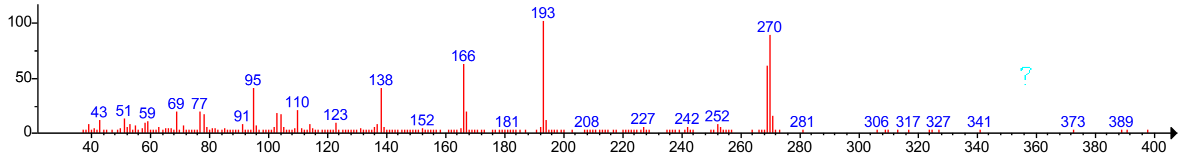

(Text File) Scan 4541 (29.219 min): TAVO-001.D\ data.ms (-4488)

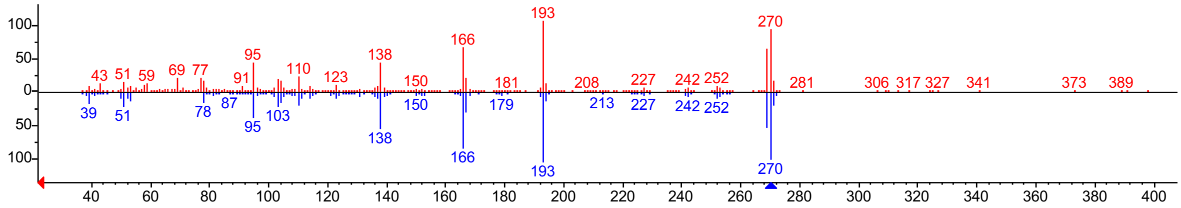

Scan 4541 (29.219 min): TAVO-001.D\ data.ms (-4488) | Head to Tail MF=876 RMF=920 | 2-Propen-1-one, 1-(2,6-dihydroxy-4-methoxyphenyl)-3-phe

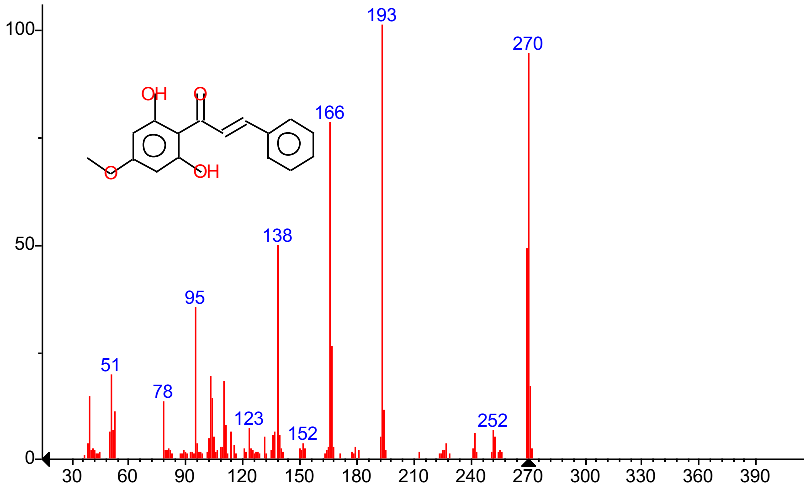

(mainlib) 2-Propen-1-one, 1-(2,6-dihydroxy-4-methoxyphenyl)-3-phenyl-, (E)-

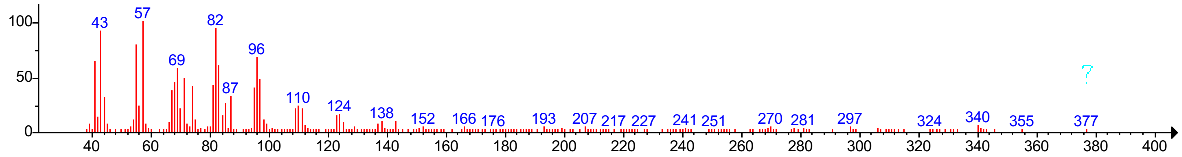

(Text File) Scan 4588 (29.521 min): TAVO-001.D\ data.ms (-4487)

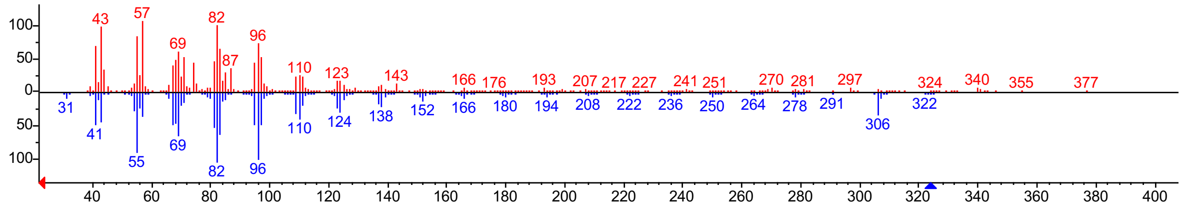

Scan 4588 (29.521 min): TAVO-001.D\ data.ms (-4487) | Head to Tail MF=723 RMF=801 | 13-Docosen-1-ol, (Z)-

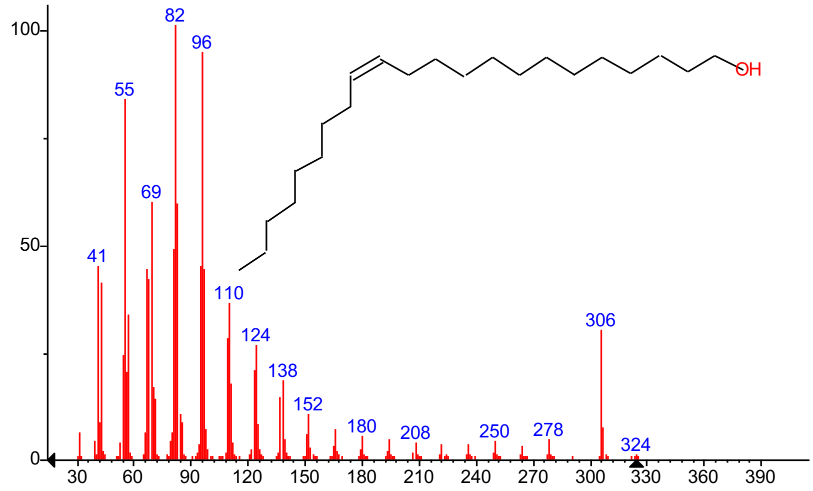

(mainlib) 13-Docosen-1-ol, (Z)-

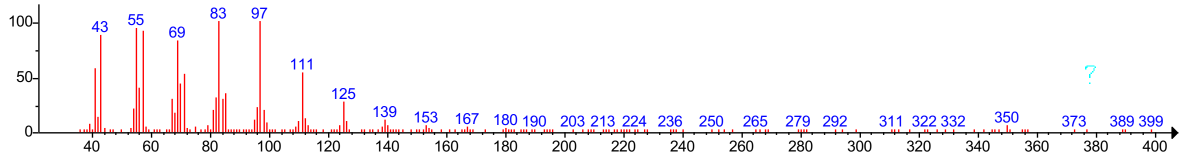

(Text File) Scan 4696 (30.214 min): TAVO-001.D\ data.ms (-4676)

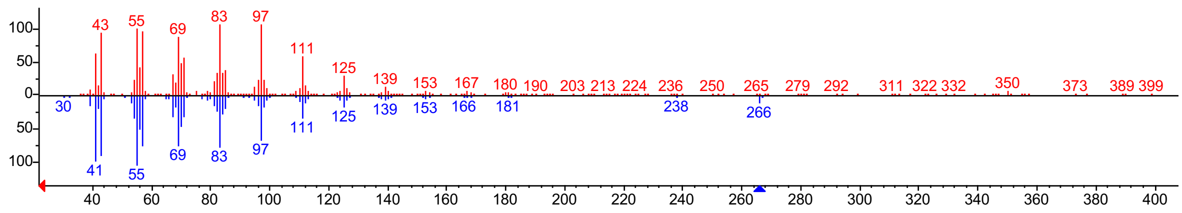

Scan 4696 (30.214 min): TAVO-001.D\ data.ms (-4676) | Head to Tail MF=836 RMF=913 | 9-Nonadecene

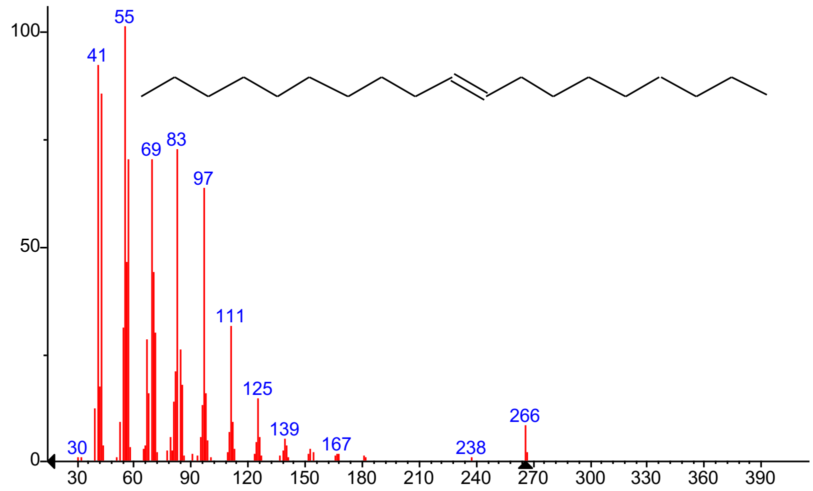

(mainlib) 9-Nonadecene

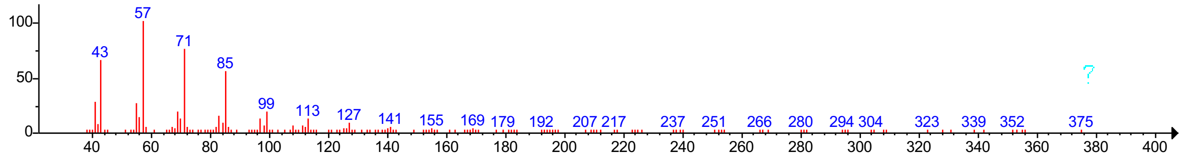

(Text File) Scan 4757 (30.605 min): TAVO-001.D\ data.ms (-4677)

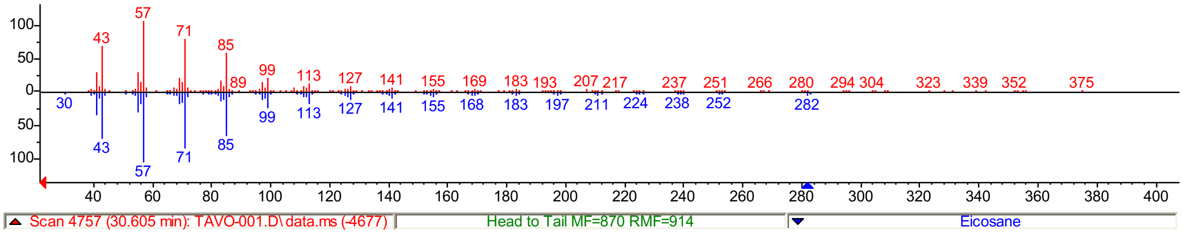

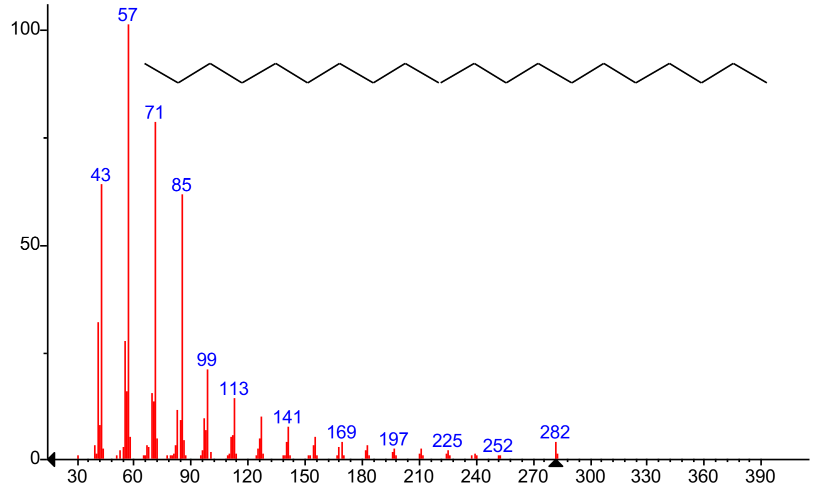

(mainlib) Eicosane

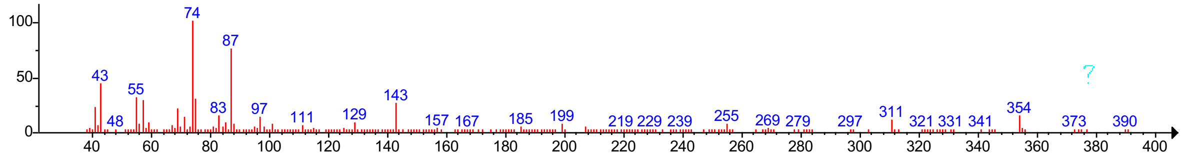

(Text File) Scan 4834 (31.099 min): TAVO-001.D\ data.ms (-4484)

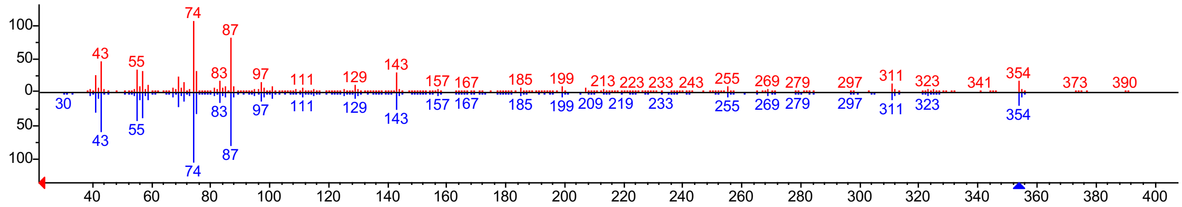

Scan 4834 (31.099 min): TAVO-001.D\ data.ms (-4484) | Head to Tail MF=890 RMF=914 | Docosanoic acid, methyl ester

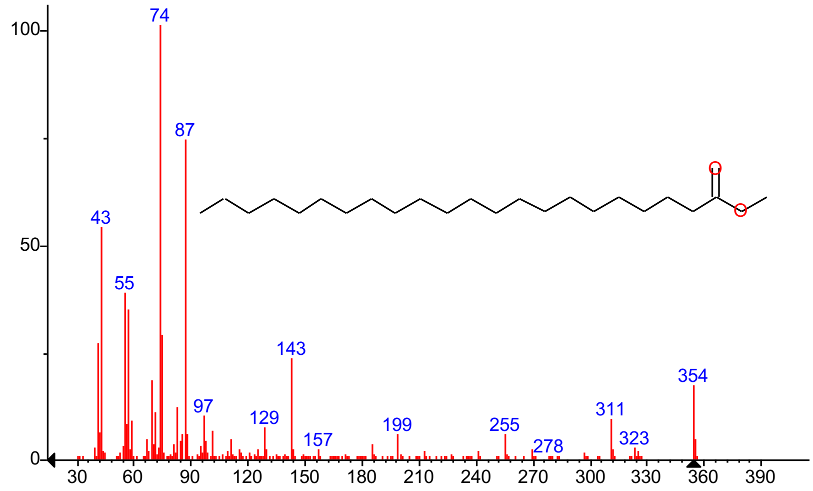

(mainlib) Docosanoic acid, methyl ester

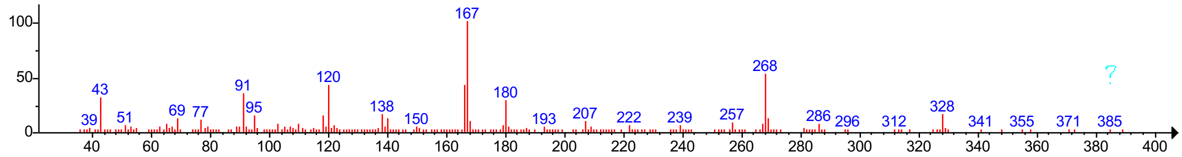

(Text File) Scan 4981 (32.042 min): TAVO-001.D\ data.ms (-4905)

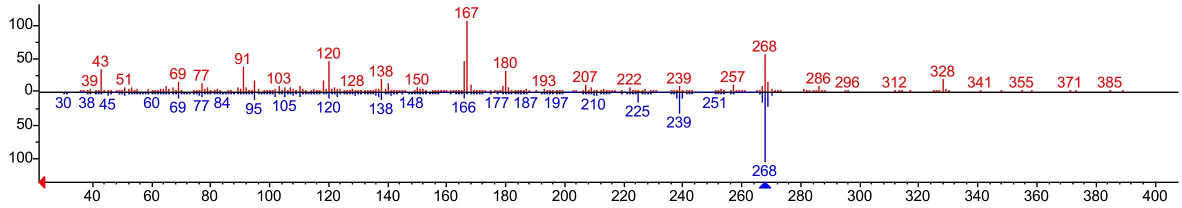

Scan 4981 (32.042 min): TAVO-001.D\ data.ms (-4905) | Head to Tail MF=519 RMF=557 | 4H-1-Benzopyran-4-one, 5-hydroxy-7-methoxy-2-phenyl-

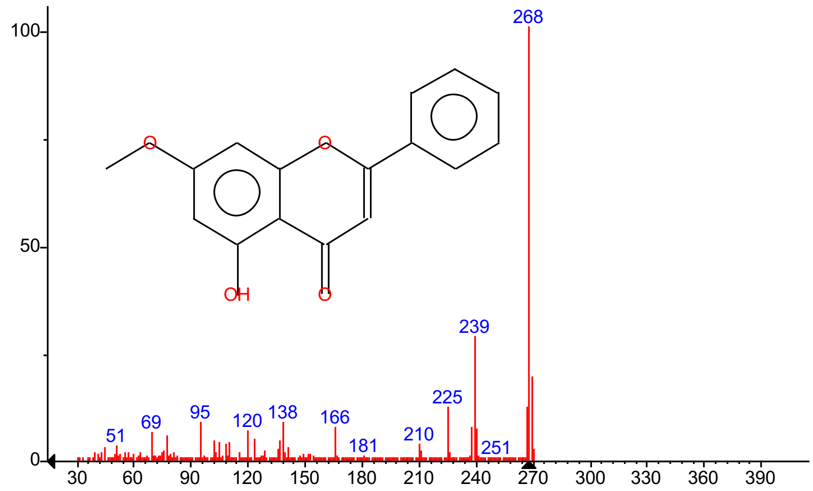

(mainlib) 4H-1-Benzopyran-4-one, 5-hydroxy-7-methoxy-2-phenyl-

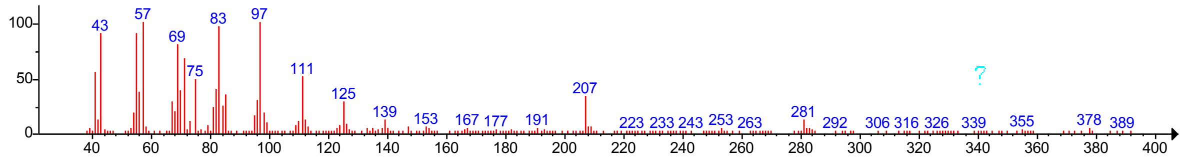

(Text File) Scan 5172 (33.267 min): TAVO-001.D\ data.ms (-4914)

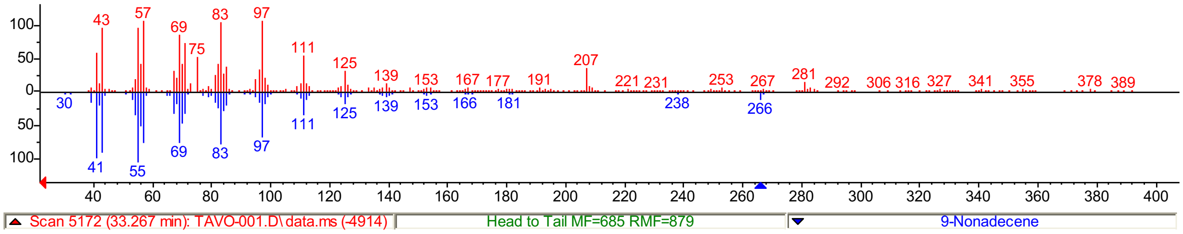

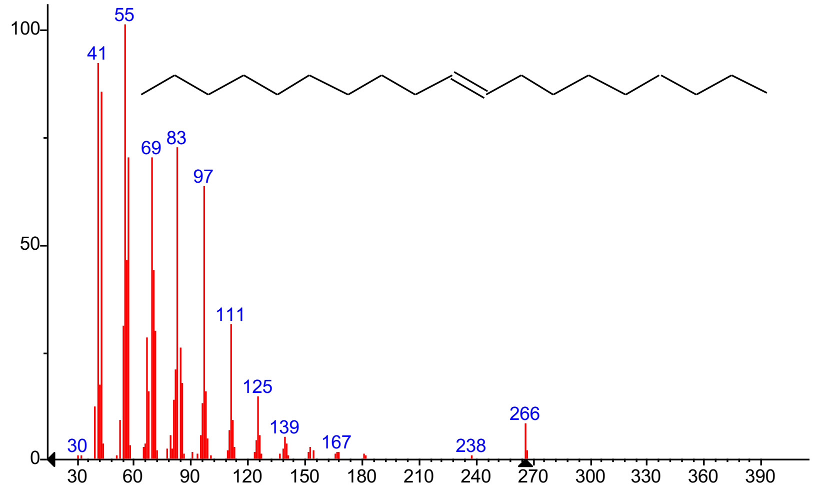

(mainlib) 9-Nonadecene

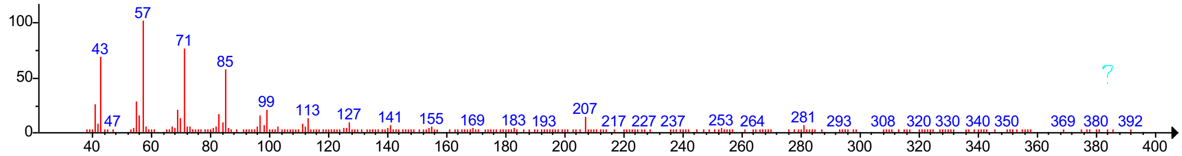

(Text File) Scan 5225 (33.607 min): TAVO-001.D\ data.ms (-4914)

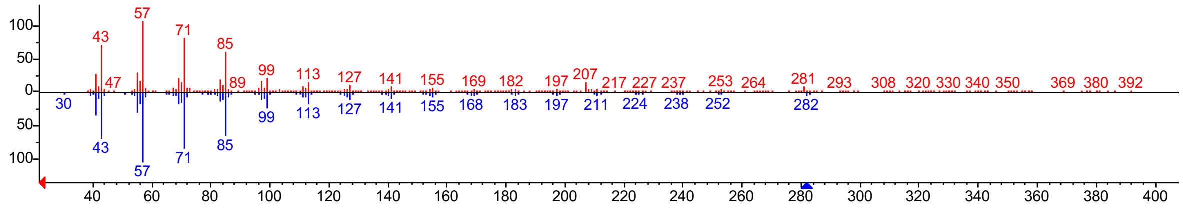

Scan 5225 (33.607 min): TAVO-001.D\ data.ms (-4914) | Head to Tail MF=762 RMF=902 | Eicosane

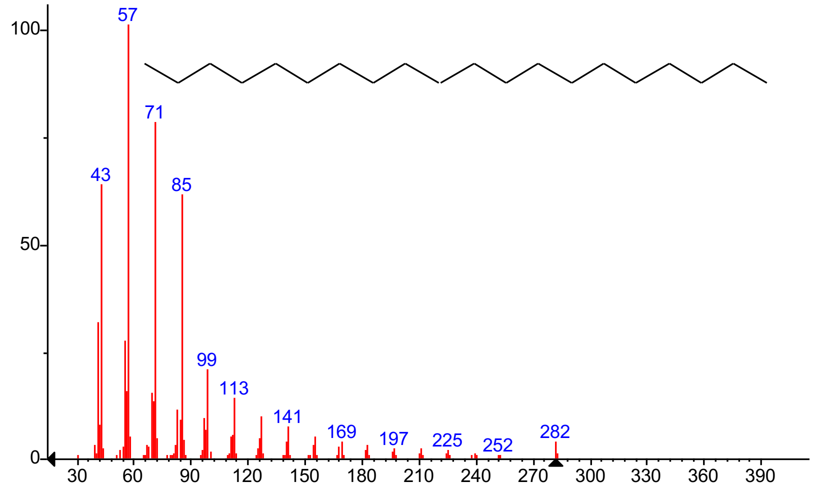

(mainlib) Eicosane

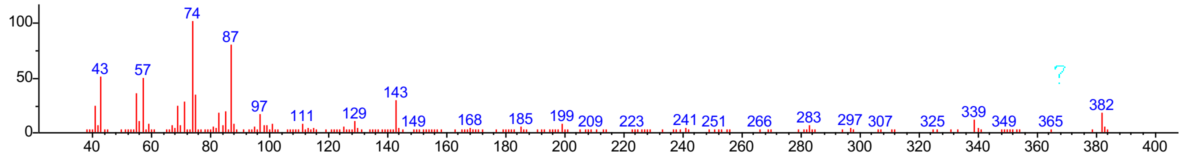

(Text File) Scan 5301 (34.095 min): TAVO-001.D\ data.ms (-5256)

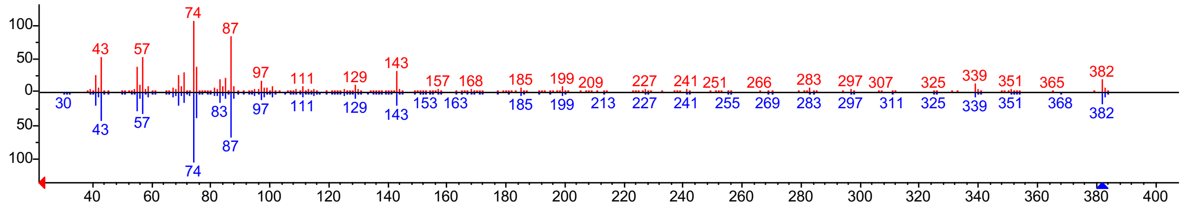

Scan 5301 (34.095 min): TAVO-001.D\ data.ms (-5256) | Head to Tail MF=876 RMF=891 | Tetracosanoic acid, methyl ester

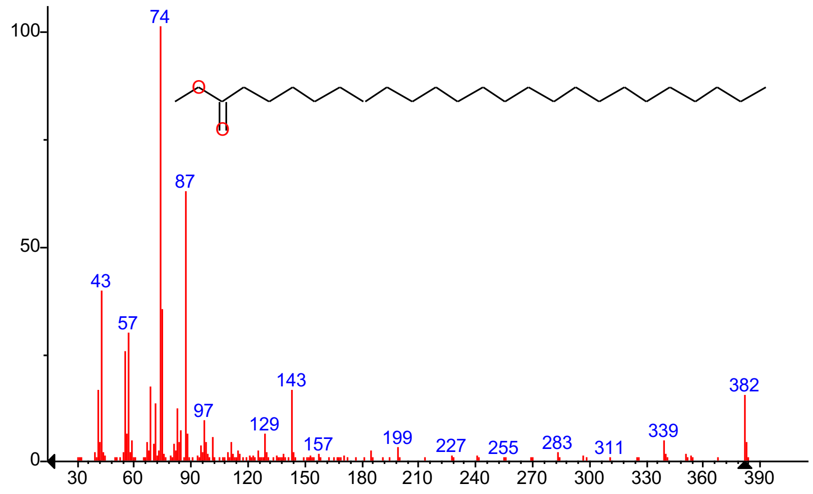

(mainlib) Tetracosanoic acid, methyl ester

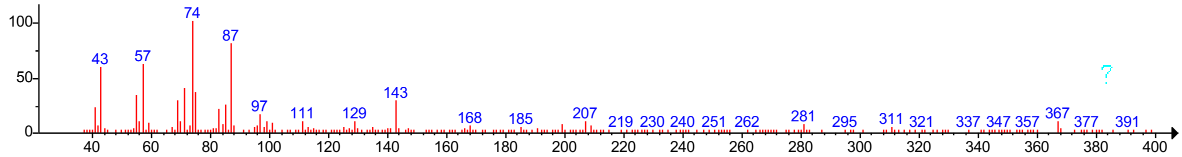

(Text File) Scan 5735 (36.879 min): TAVO-001.D\ data.ms (-5700)

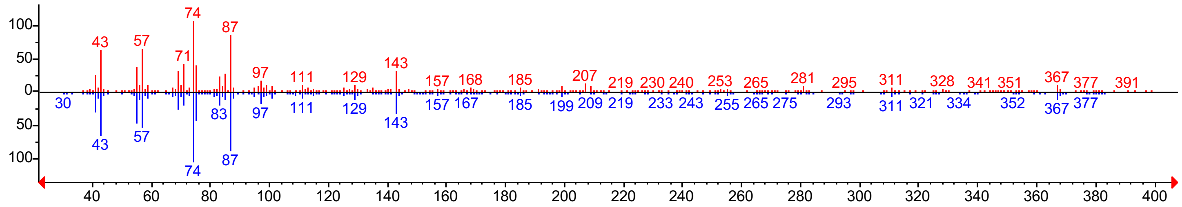

Scan 5735 (36.879 min): TAVO-001.D\ data.ms (-5700) | Head to Tail MF=744 RMF=825 | Hexacosanoic acid, methyl ester

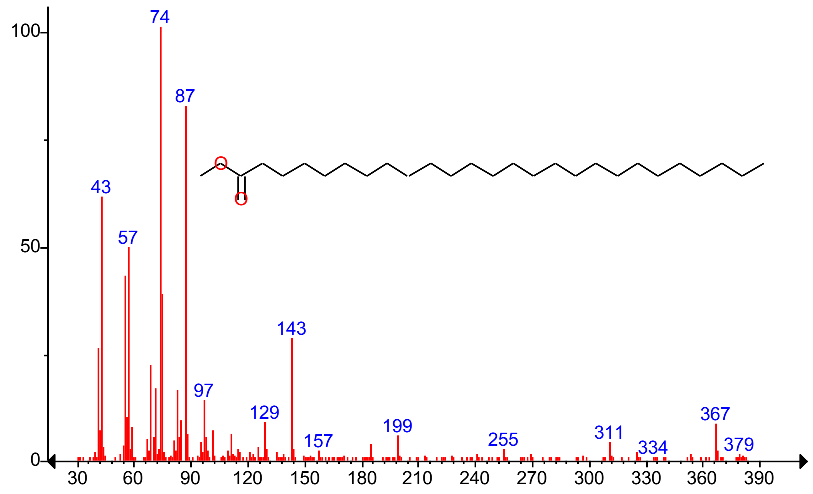

(mainlib) Hexacosanoic acid, methyl ester

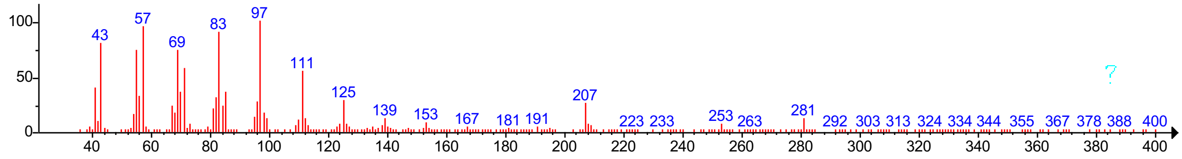

(Text File) Scan 6025 (38.740 min): TAVO-001.D\ data.ms (-5872)

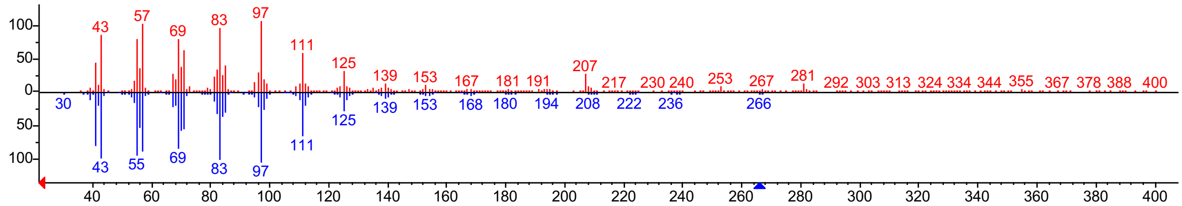

Scan 6025 (38.740 min): TAVO-001.D\ data.ms (-5872) | Head to Tail MF=711 RMF=836 | 1-Nonadecene

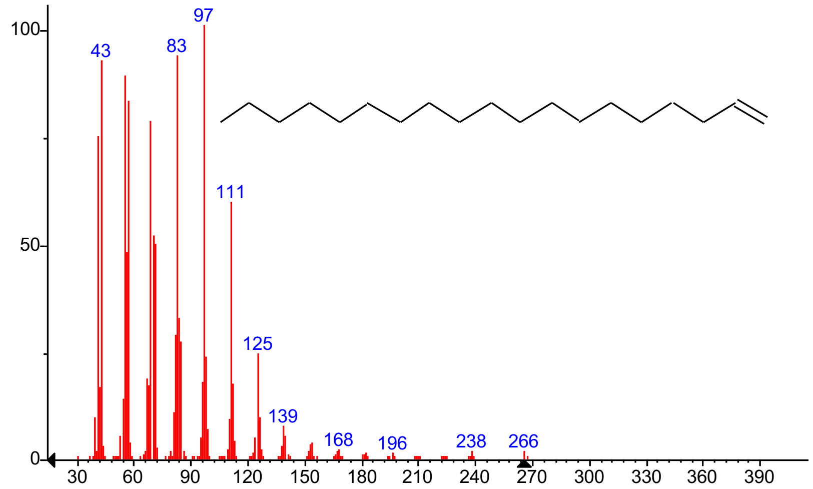

(replib) 1-Nonadecene

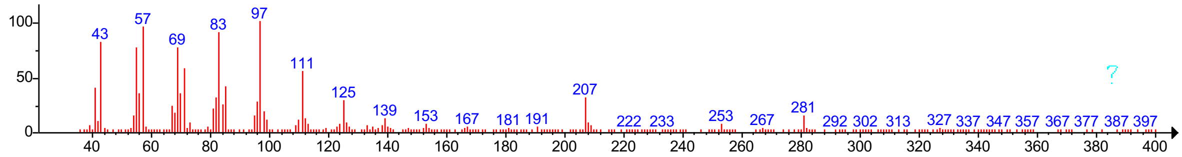

(Text File) Scan 6039 (38.830 min): TAVO-001.D\ data.ms (-5876)

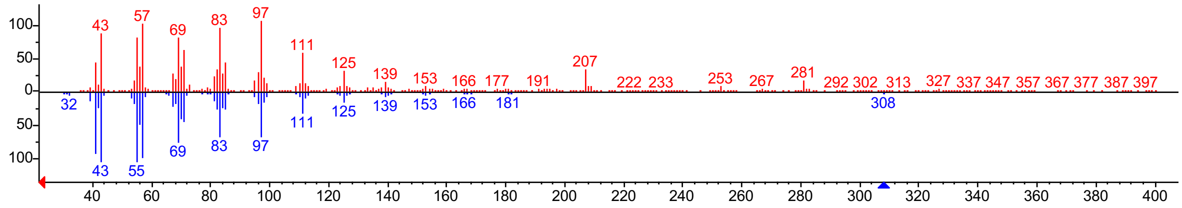

Scan 6039 (38.830 min): TAVO-001.D\ data.ms (-5876) | Head to Tail MF=732 RMF=918 | 1-Docosene

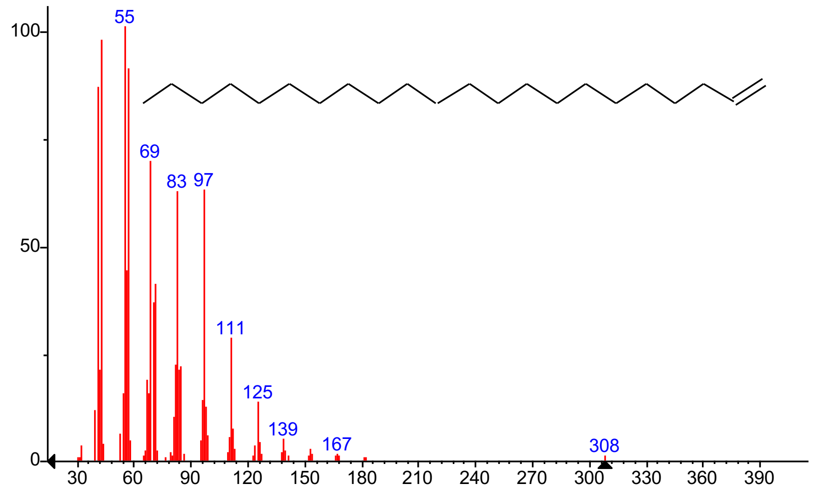

(replib) 1-Docosene

Ethyl Acetate fraction of ChEEP.

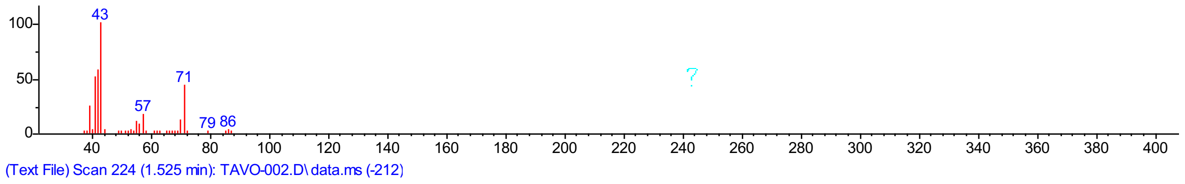

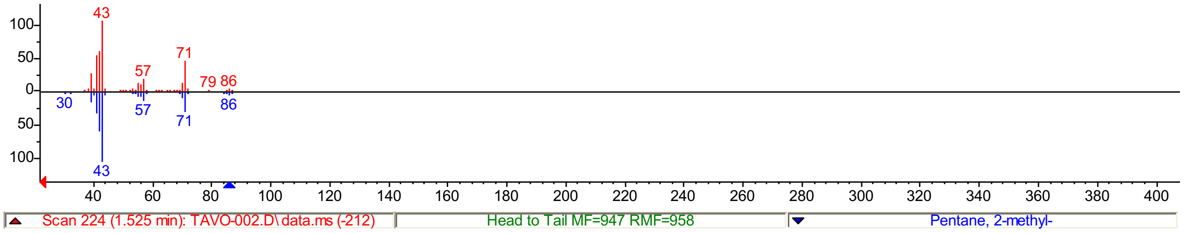

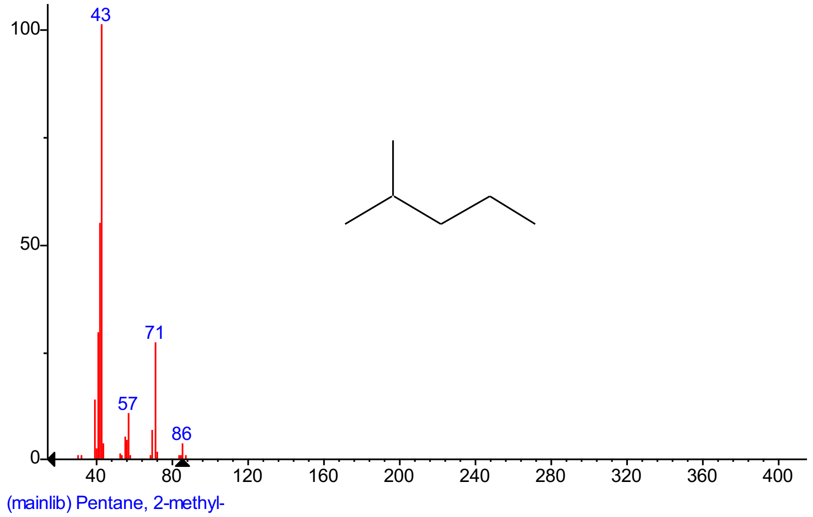

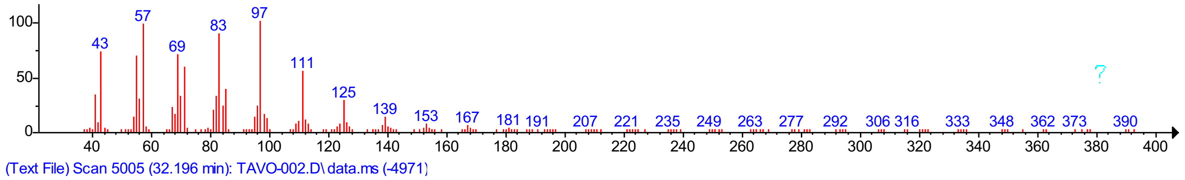

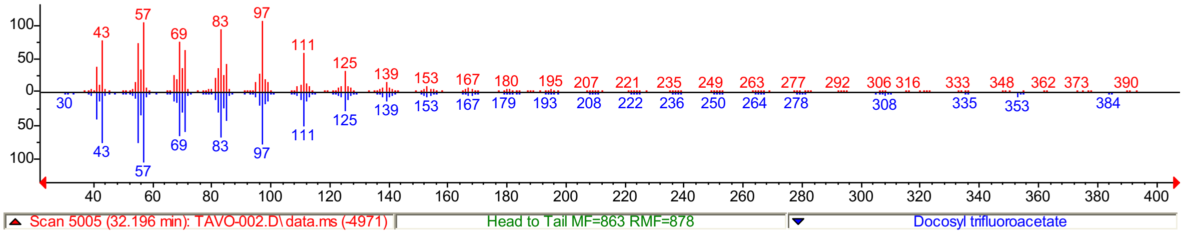

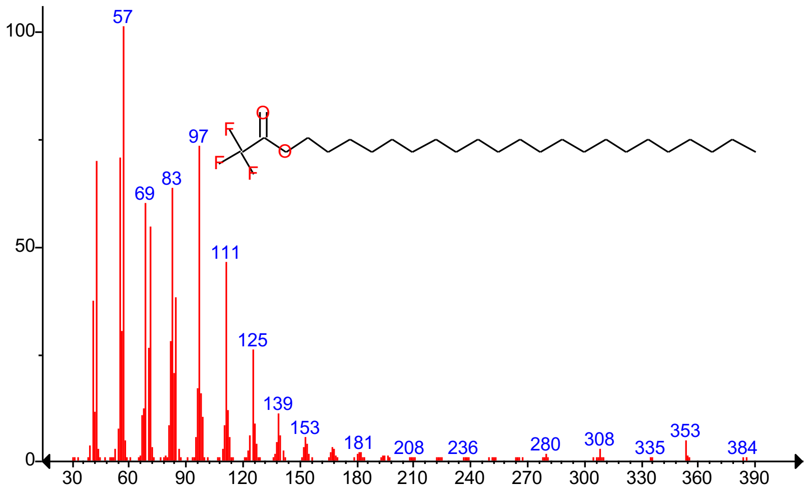

(mainlib) Docosyl trifluoroacetate

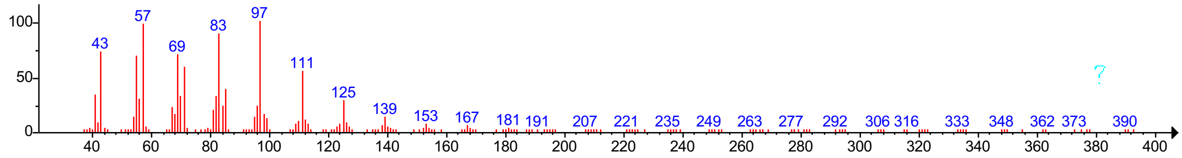

(Text File) Scan 5005 (32.196 min): TAVO-002.D\ data.ms (-4971)

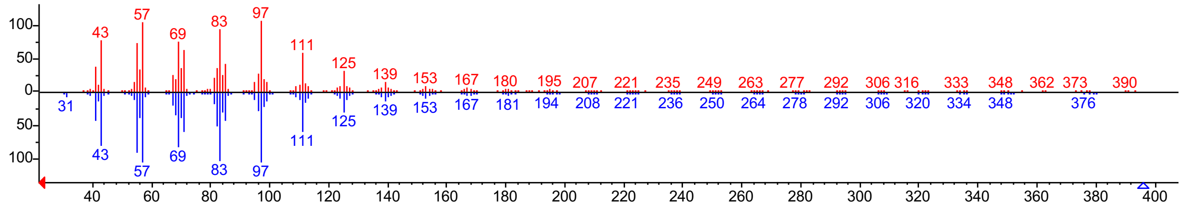

Scan 5005 (32.196 min): TAVO-002.D\ data.ms (-4971) | Head to Tail MF=895 RMF=907 | 1-Heptacosanol

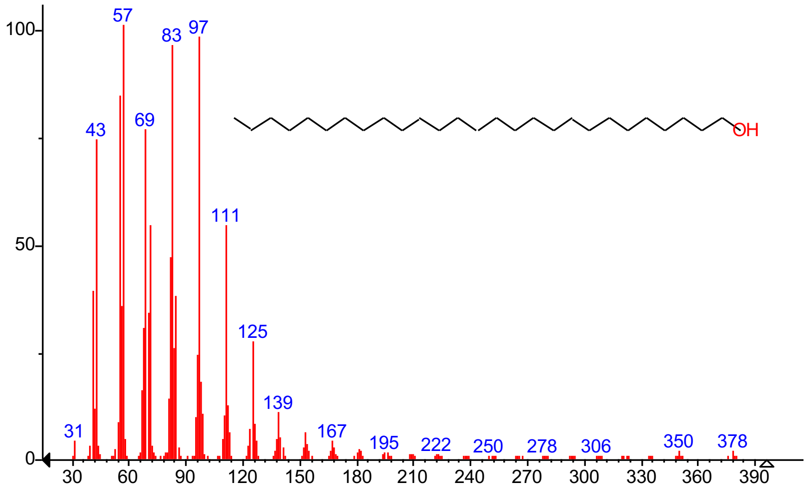

(mainlib) 1-Heptacosanol

Derivatization of ChEEP.

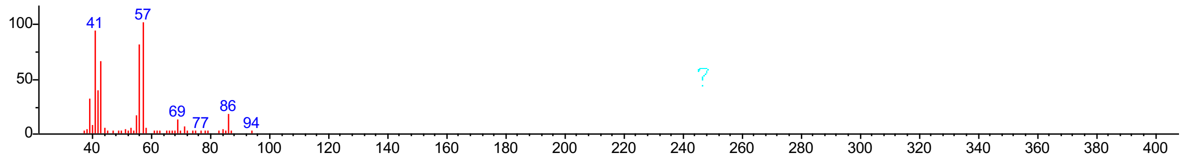

(Text File) Scan 248 (1.679 min): TAVO-003.D\ data.ms (-190)

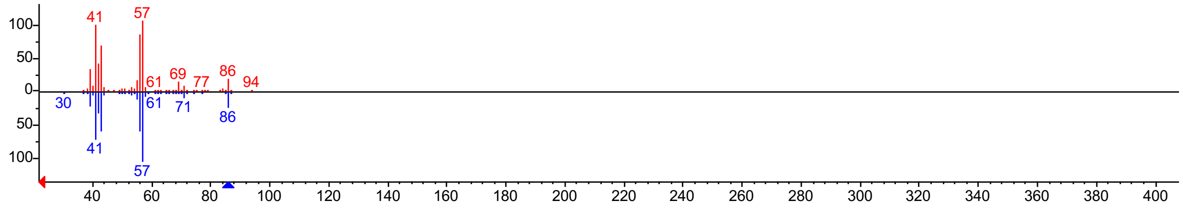

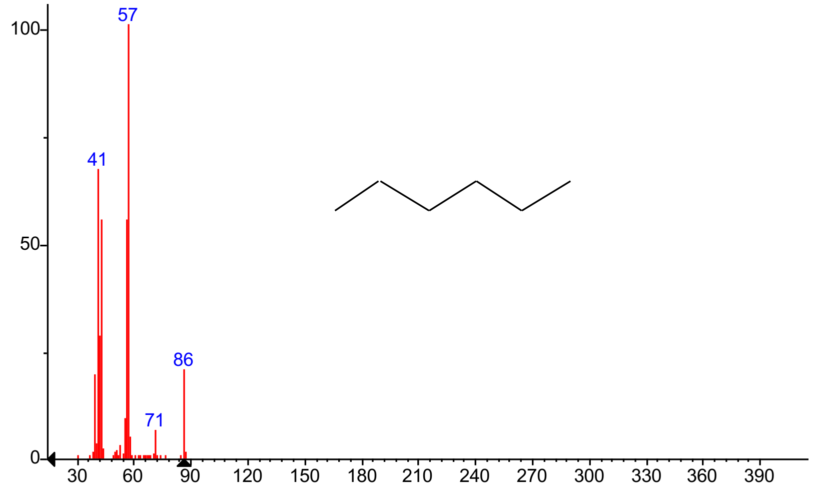

(replib) Hexane

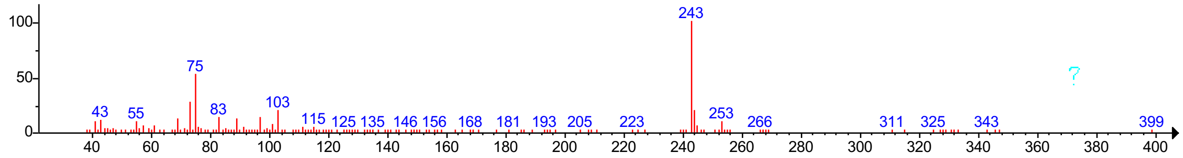

(Text File) Scan 2046 (13.213 min): TAVO-003.D\ data.ms (-1832)

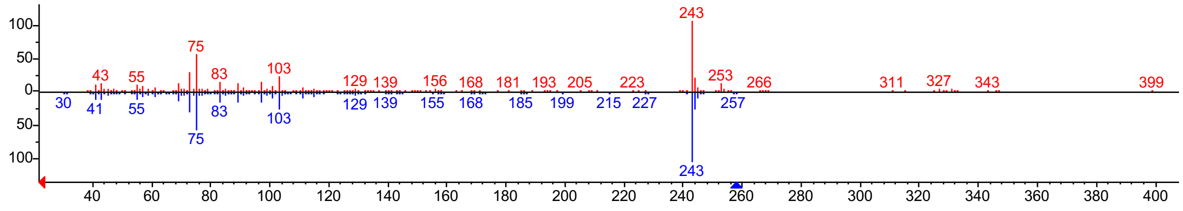

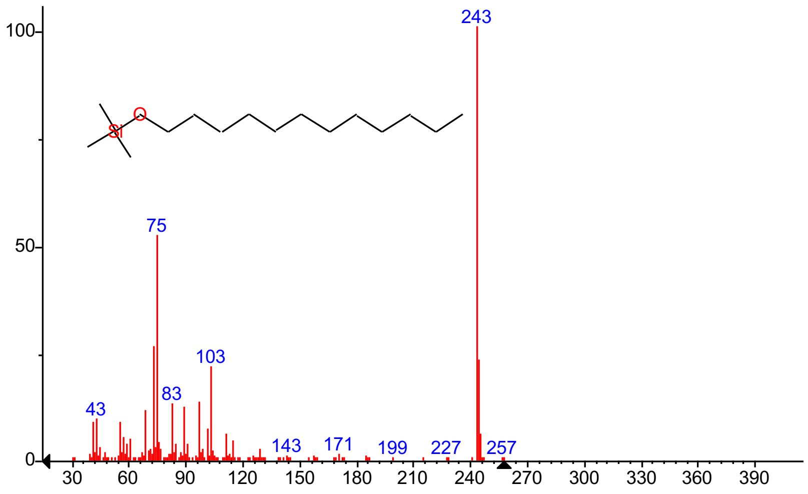

(mainlib) Silane, (dodecyloxy)trimethyl-

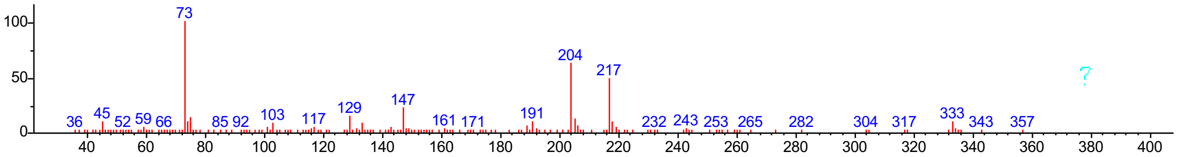

(Text File) Scan 2573 (16.594 min): TAVO-003.D\ data.ms (-2547)

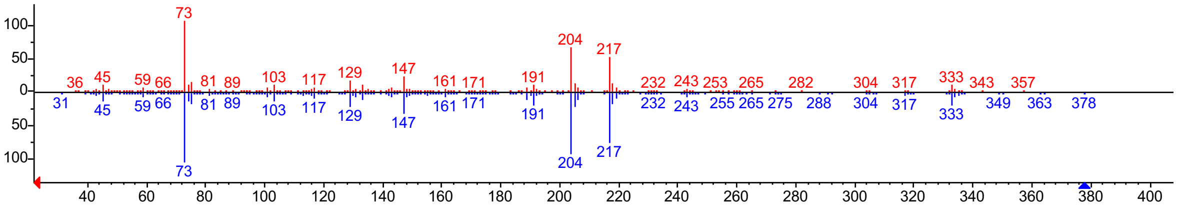

Scan 2573 (16.594 min): TAVO-003.D\ data.ms (-2547) | Head to Tail MF=887 RMF=899 | 1,6-Anhydro-ß-d-glucose, tris(trimethylsilyl) ether

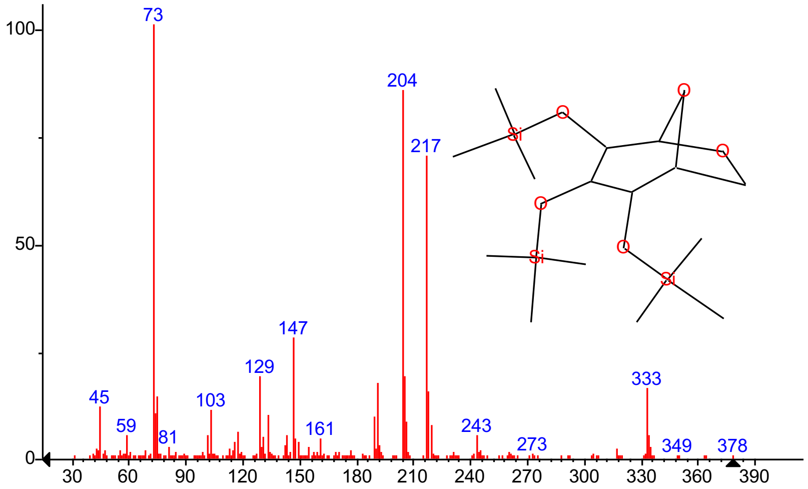

(mainlib) 1,6-Anhydro-ß-d-glucose, tris(trimethylsilyl) ether

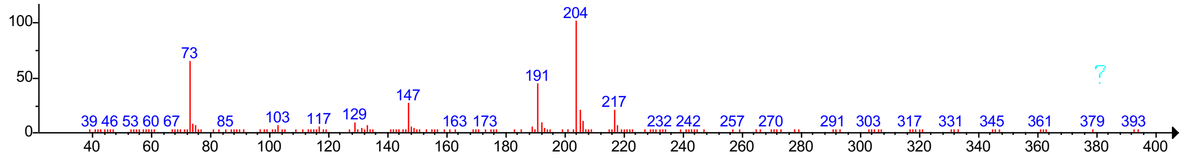

(Text File) Scan 3202 (20.629 min): TAVO-003.D\ data.ms (-3169)

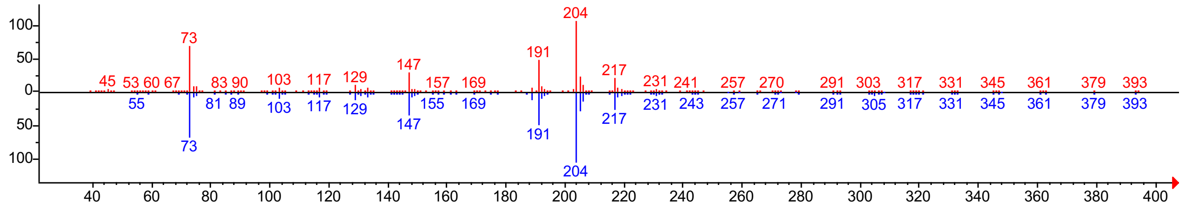

Scan 3202 (20.629 min): TAVO-003.D\ data.ms (-3169) | Head to Tail MF=910 RMF=919 | Glucopyranose, 1,2,3,4,6-pentakis-O-(trimethylsilyl)-, D-

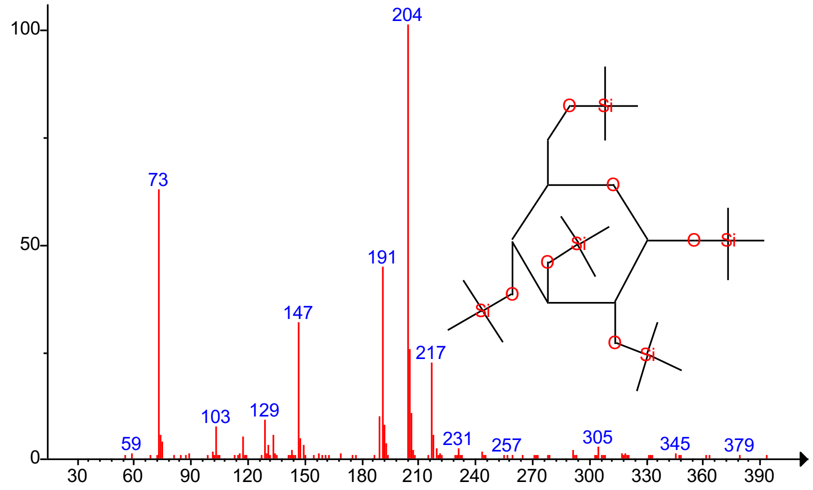

(mainlib) Glucopyranose, 1,2,3,4,6-pentakis-O-(trimethylsilyl)-, D-

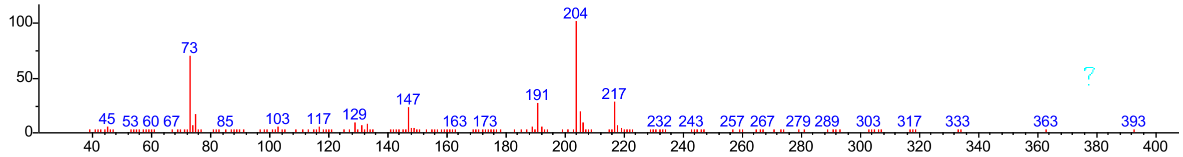

(Text File) Scan 3283 (21.149 min): TAVO-003.D\ data.ms (-3251)

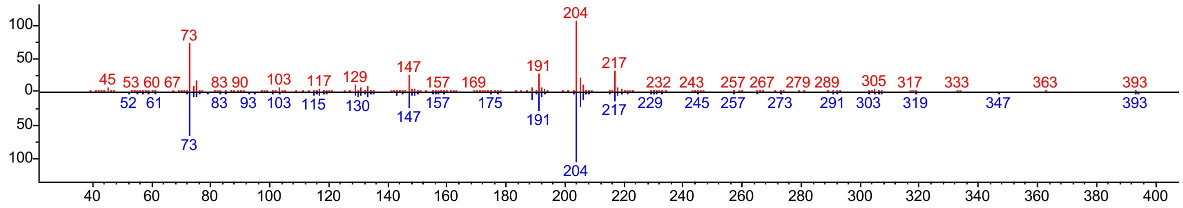

Scan 3283 (21.149 min): TAVO-003.D\ data.ms (-3251) | Head to Tail MF=852 RMF=881 | Mannose, 6-deoxy-2,3,4,5-tetrakis-O-(trimethylsilyl)-, L-

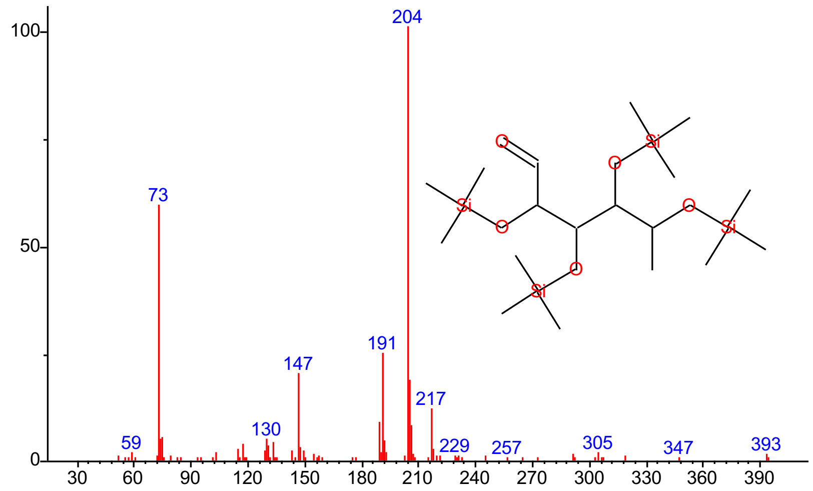

(replib) Mannose, 6-deoxy-2,3,4,5-tetrakis-O-(trimethylsilyl)-, L-

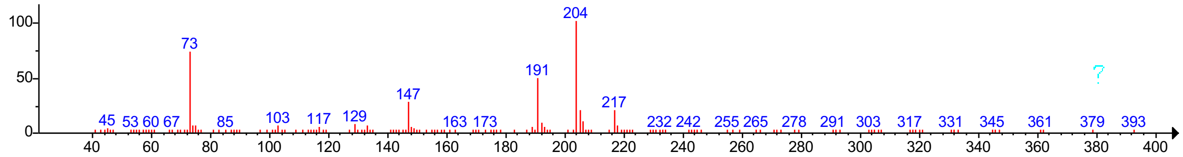

(Text File) Scan 3479 (22.407 min): TAVO-003.D\ data.ms (-3451)

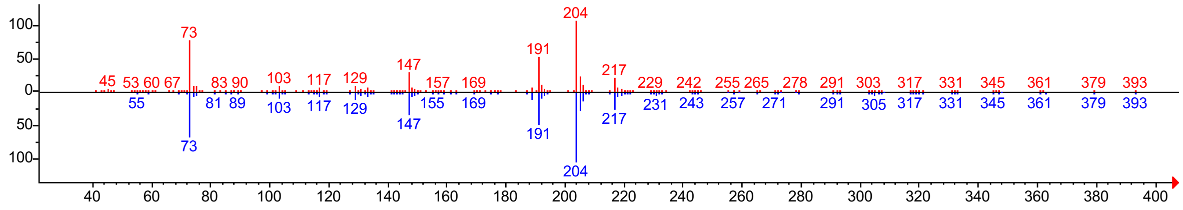

Scan 3479 (22.407 min): TAVO-003.D\ data.ms (-3451) | Head to Tail MF=925 RMF=932 | Glucopyranose, 1,2,3,4,6-pentakis-O-(trimethylsilyl)-, D-

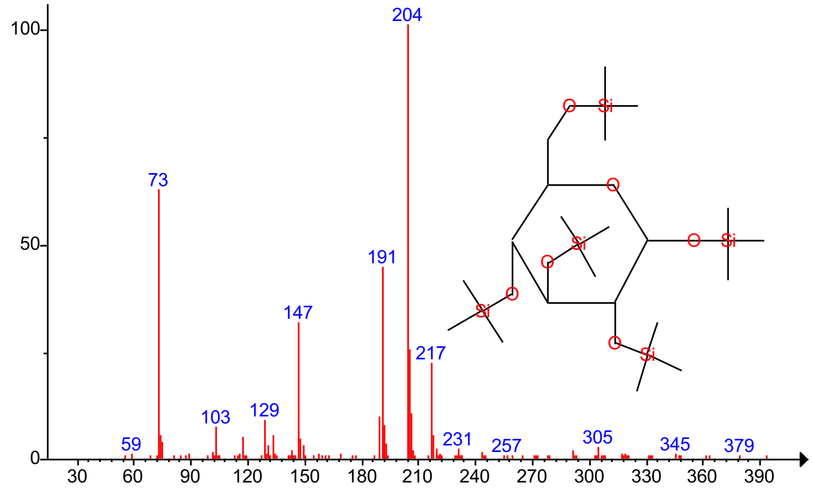

(mainlib) Glucopyranose, 1,2,3,4,6-pentakis-O-(trimethylsilyl)-, D-

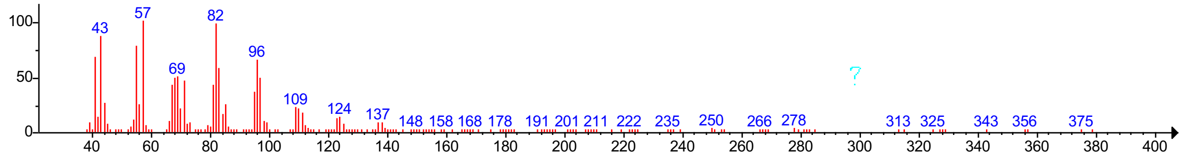

(Text File) Scan 4059 (26.127 min): TAVO-003.D\ data.ms (-4048)

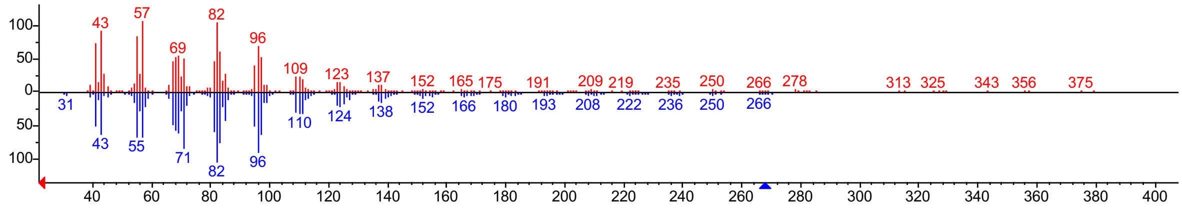

Scan 4059 (26.127 min): TAVO-003.D\ data.ms (-4048) | Head to Tail MF=797 RMF=827 | Oxirane, hexadecyl-

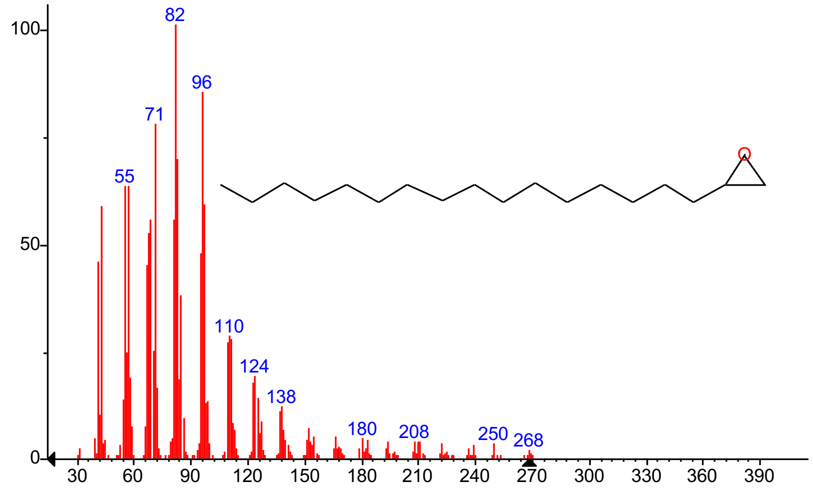

(replib) Oxirane, hexadecyl-

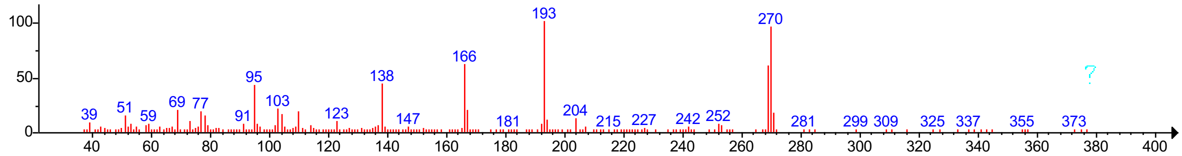

(Text File) Scan 4543 (29.232 min): TAVO-003.D\ data.ms (-4512)

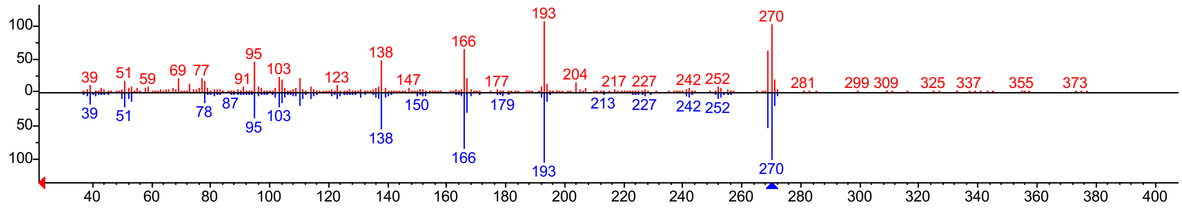

Scan 4543 (29.232 min): TAVO-003.D\ data.ms (-4512) | Head to Tail MF=804 RMF=898 | 2-Propen-1-one, 1-(2,6-dihydroxy-4-methoxyphenyl)-3-phe

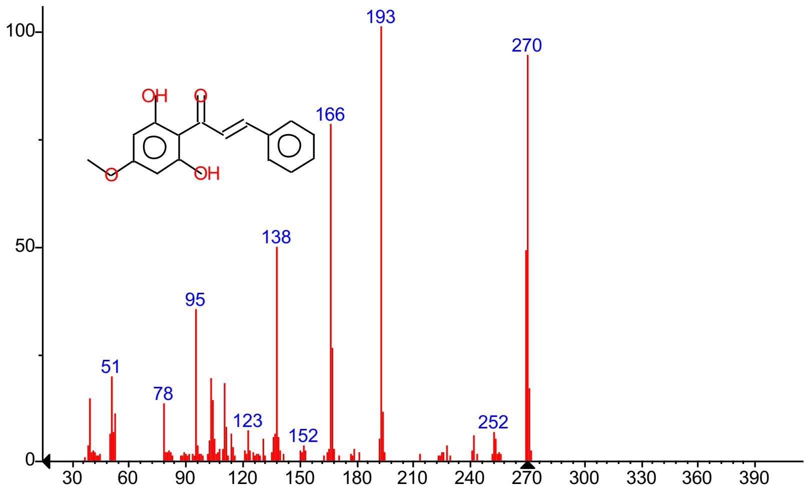

(mainlib) 2-Propen-1-one, 1-(2,6-dihydroxy-4-methoxyphenyl)-3-phenyl-, (E)-

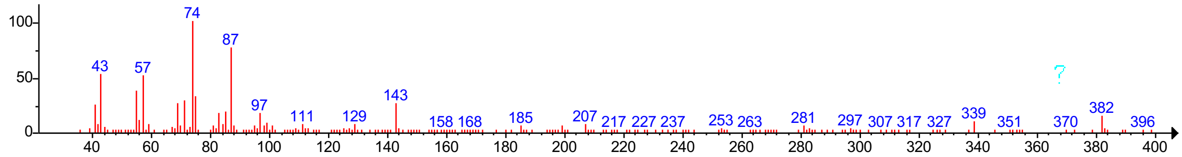

(Text File) Scan 5299 (34.082 min): TAVO-003.D\ data.ms (-5287)

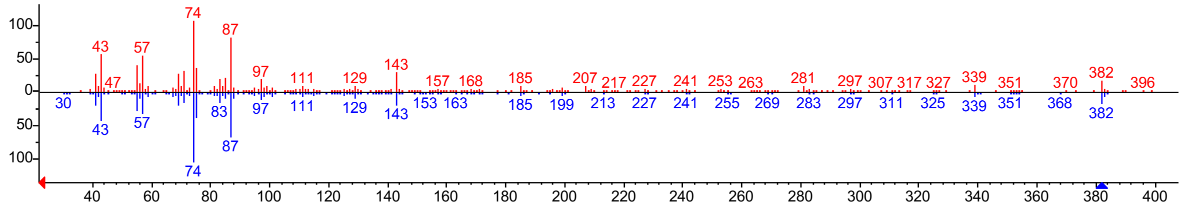

Scan 5299 (34.082 min): TAVO-003.D\ data.ms (-5287) | Head to Tail MF=777 RMF=864 | Tetracosanoic acid, methyl ester

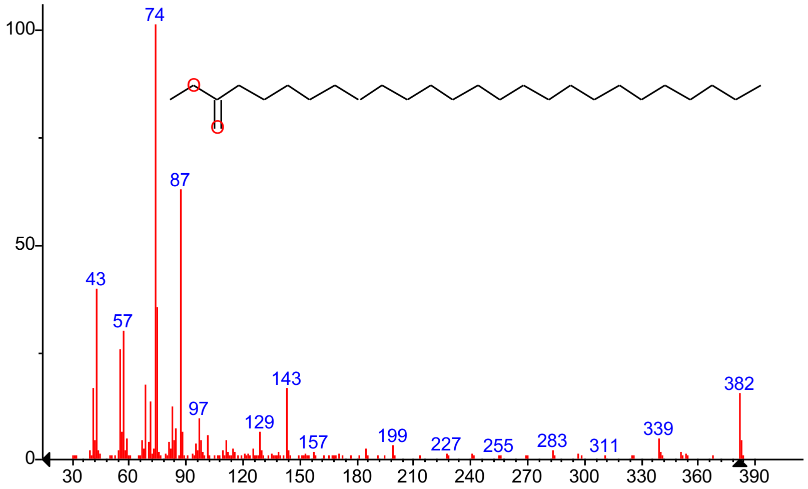

(mainlib) Tetracosanoic acid, methyl ester

Figure S2. HPLC–DAD chromatograms of the fractions obtained from the EA fraction of ChEEP (a-w).

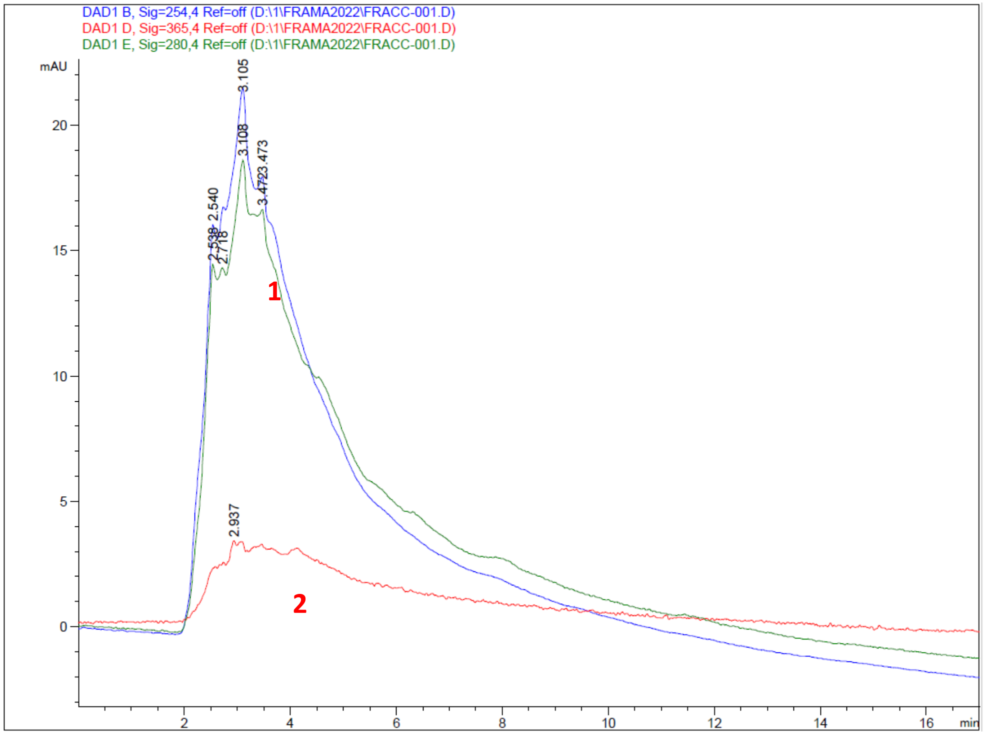

a.- Chromatogram fraction one (1.- Catechol, 2.- Catechine).

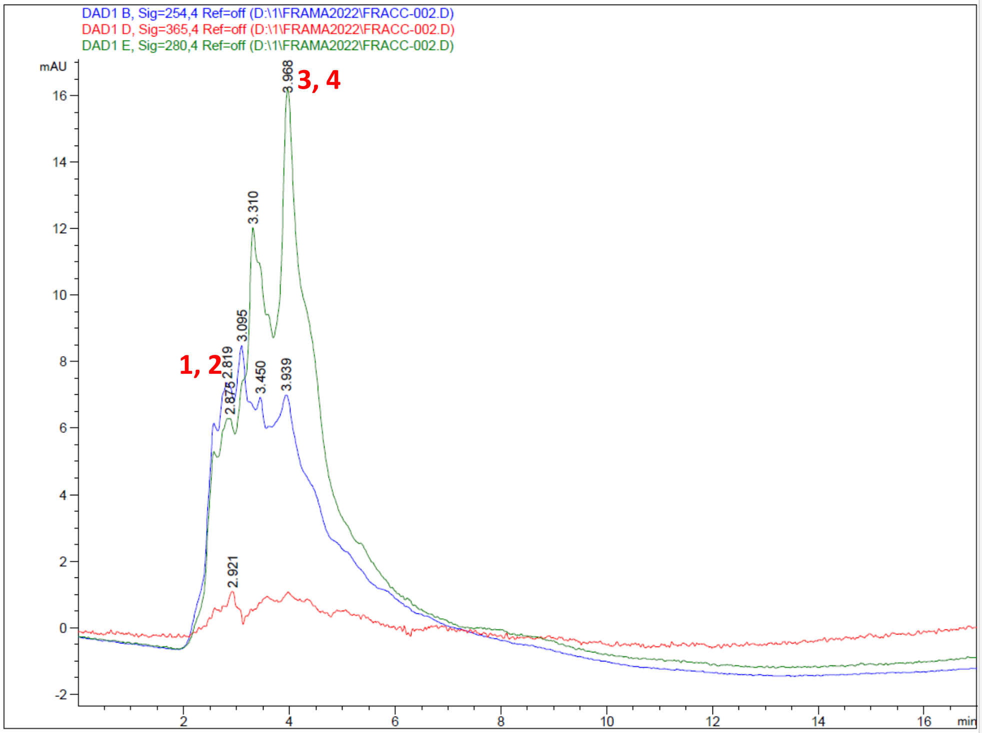

b.- Chromatogram fraction two (1.- Catechin, 2.- Catechol, 3.- Naringenin, 4.- Naringin).

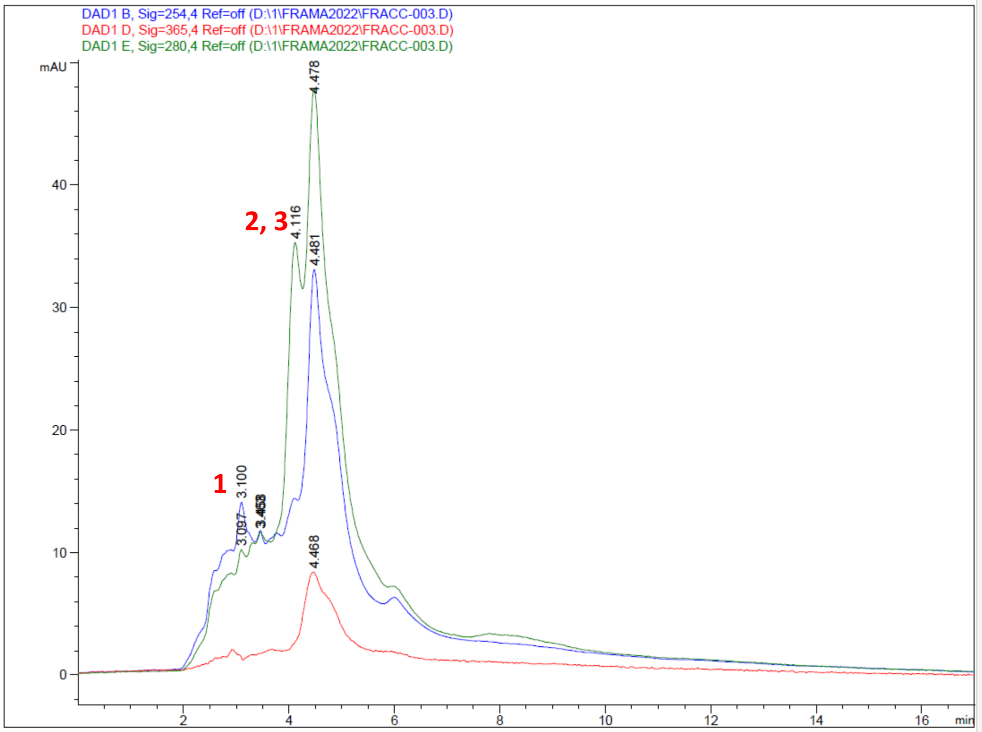

c.- Chromatogram fraction three (1.- Catechin, 2.- Naringin, 3.- Naringenin).

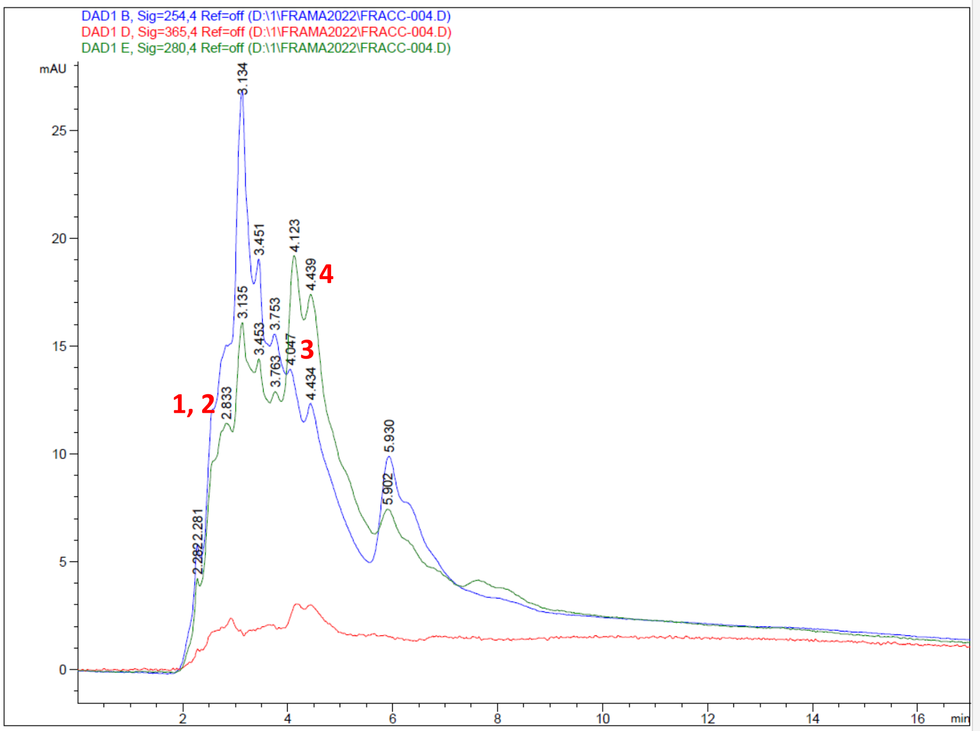

d.- Chromatogram fraction four (1.- Catechin, 2.- Catechol, 3.- Naringin, 4.- Naringenin).

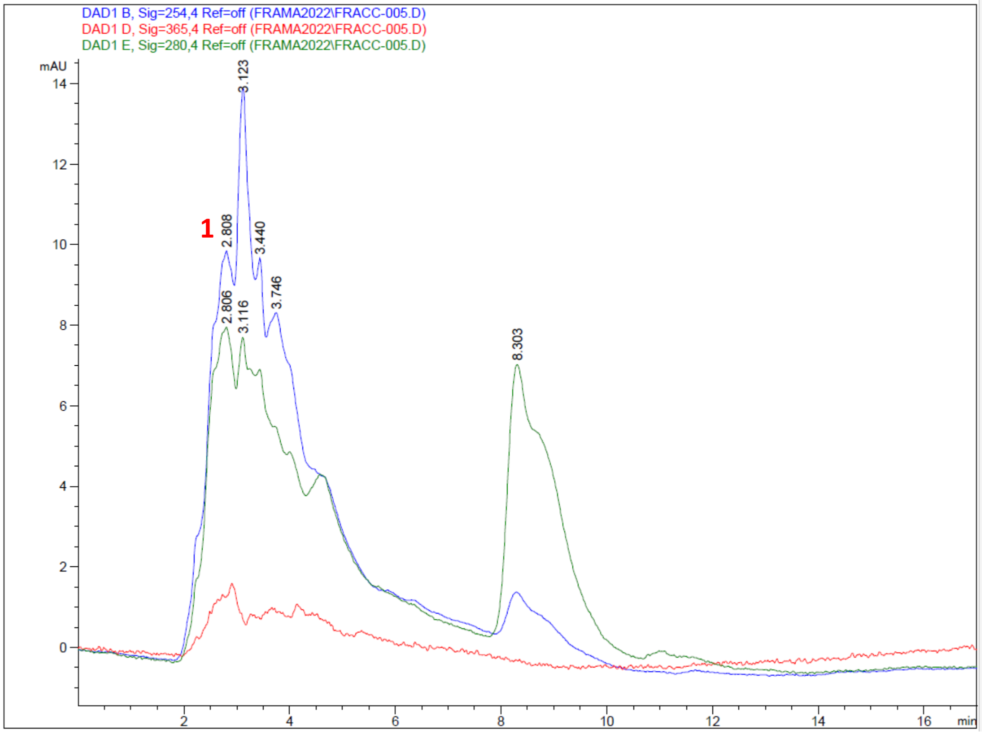

e.- Chromatogram fraction five (1.- Catechin).

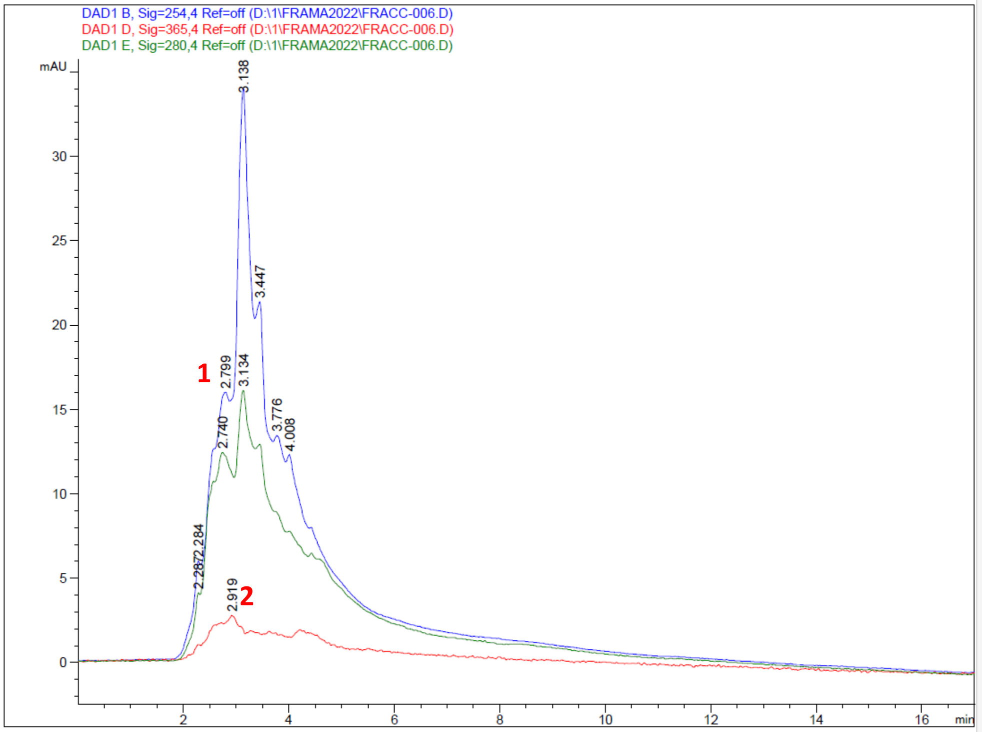

f.- Chromatogram fraction six (1.- Catechin, 2.- Catechol).

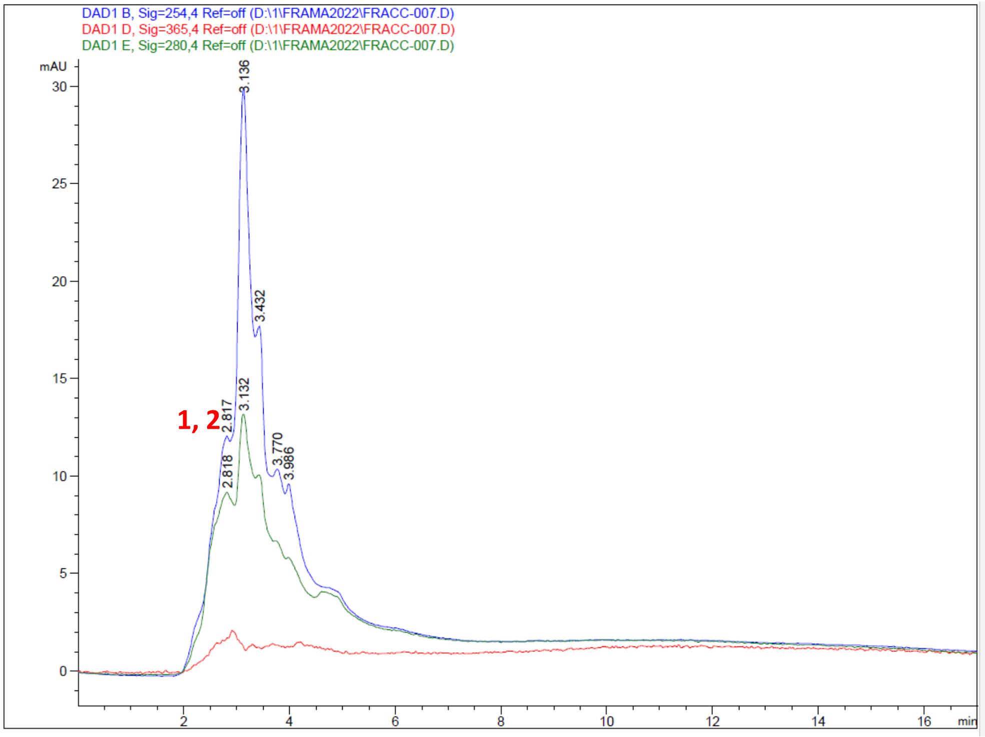

g.- Chromatogram fraction seven (1.- Catechin, 2.- Catechol).

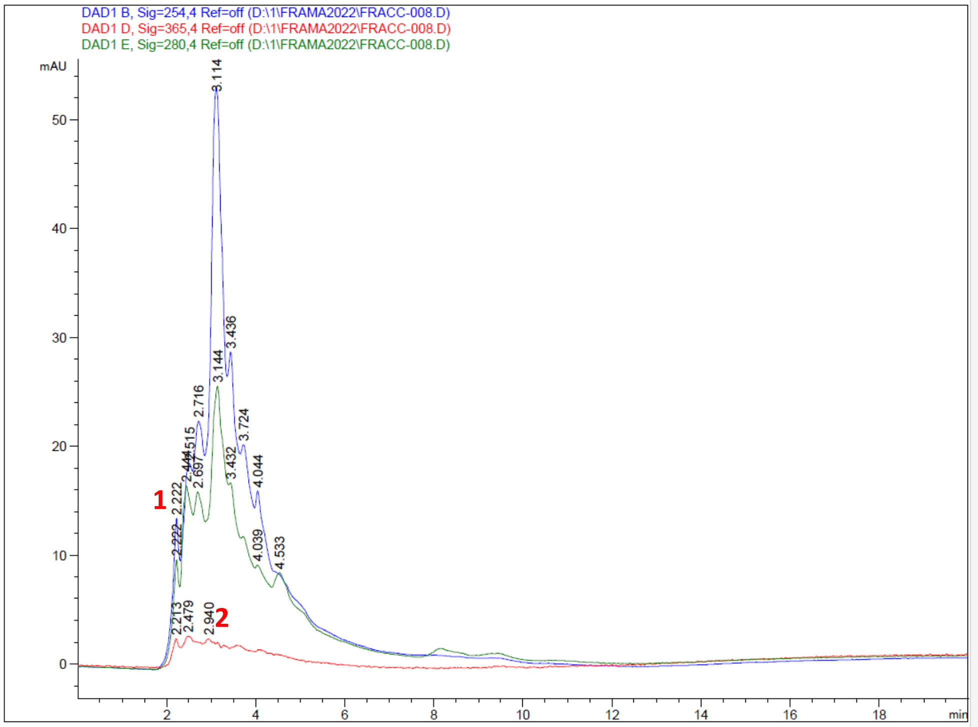

h.- Chromatogram fraction eight (1.- Catechin, 2.- Catechol).

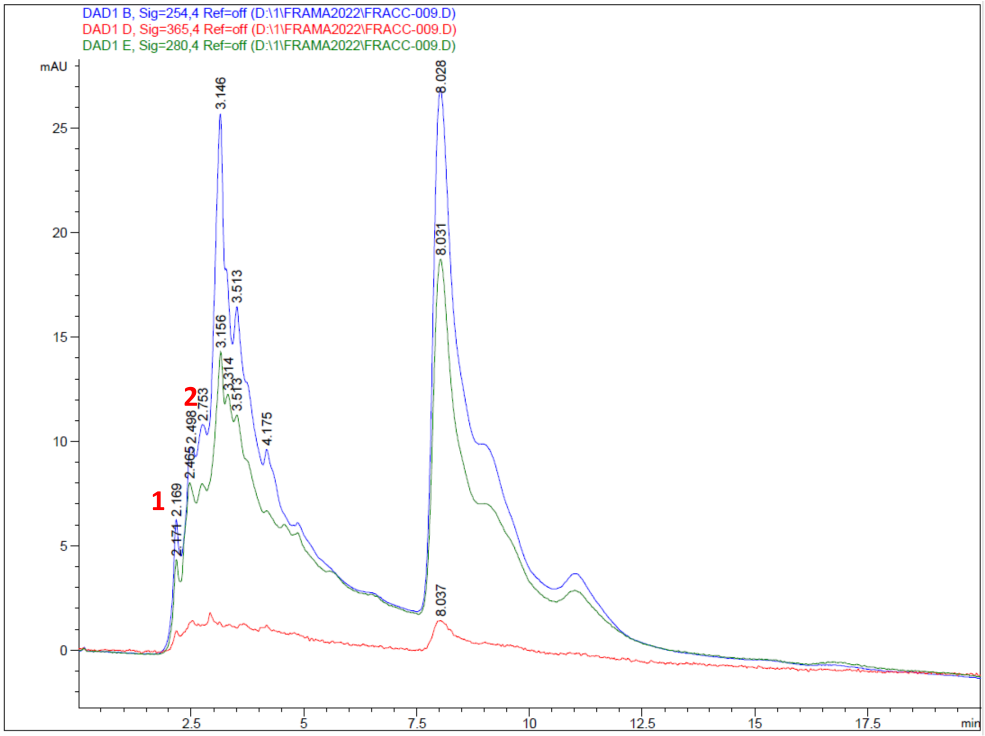

i.- Chromatogram fraction nine (1.- Catechin, 2.- Catechol).

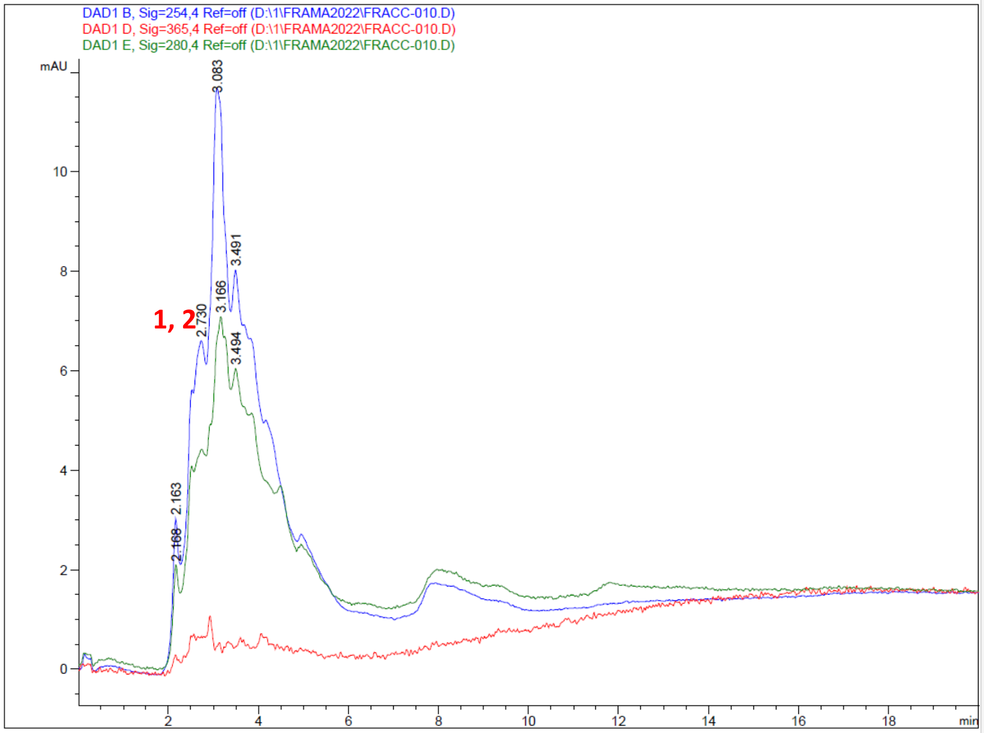

j.- Chromatogram fraction ten (1.- Catechin, 2.- Catechol).

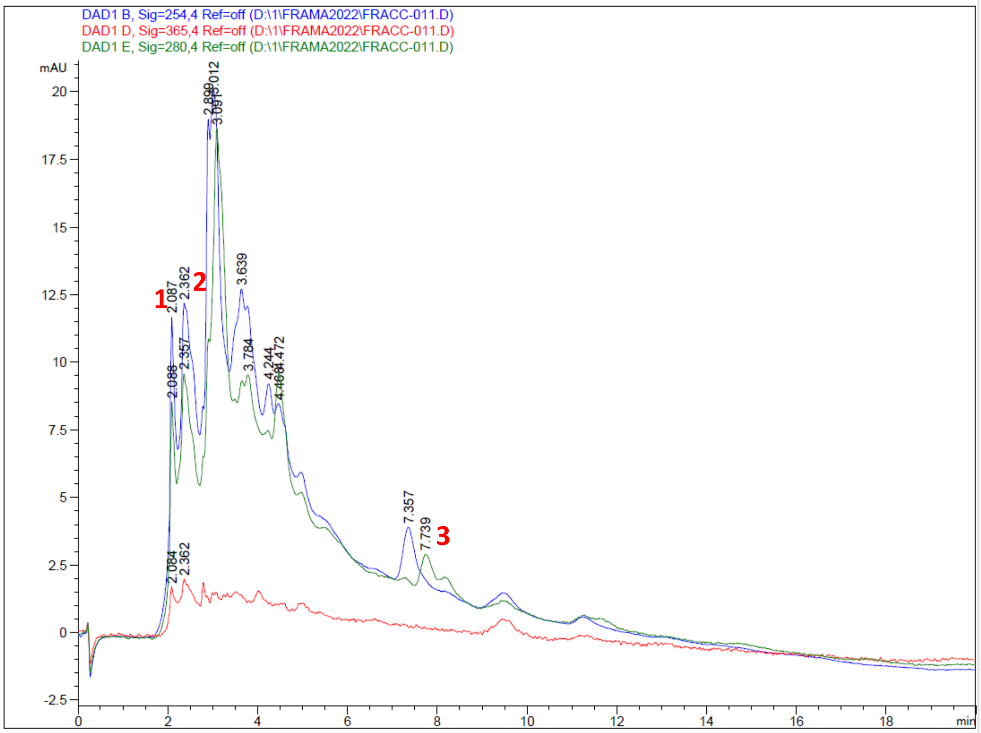

k.- Chromatogram fraction eleven (1.- Catechin, 2.- Catechol, 3.- Naringenin).

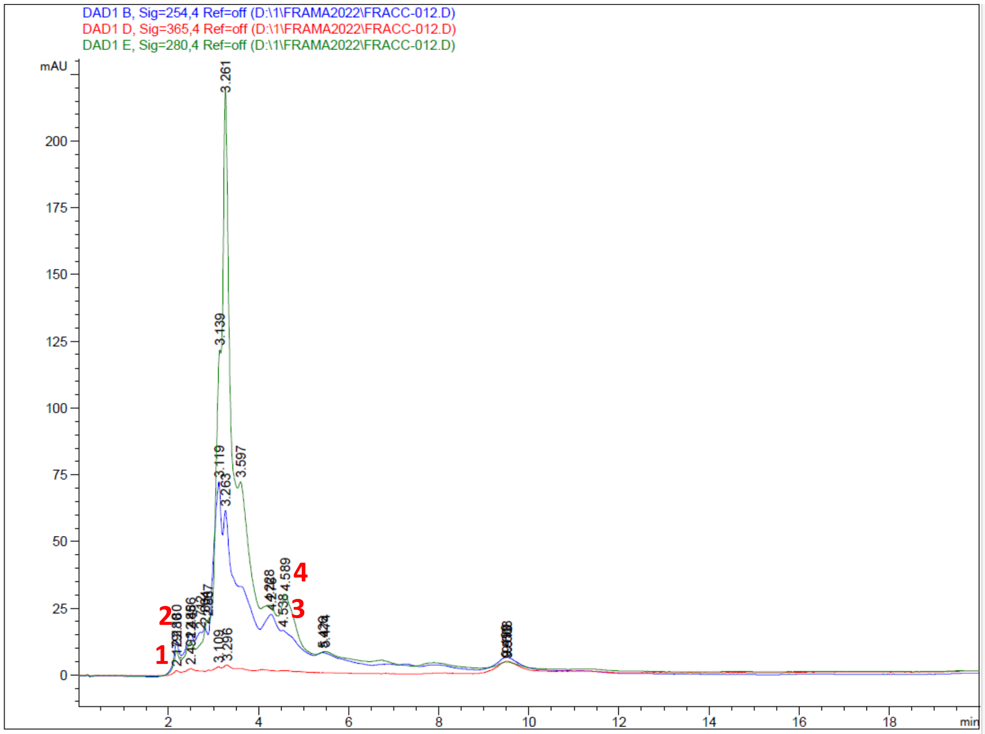

l.- Chromatogram fraction twelve (1.- Catechin, 2.- Catechol, 3.- Naringenin, 4.- Naringin).

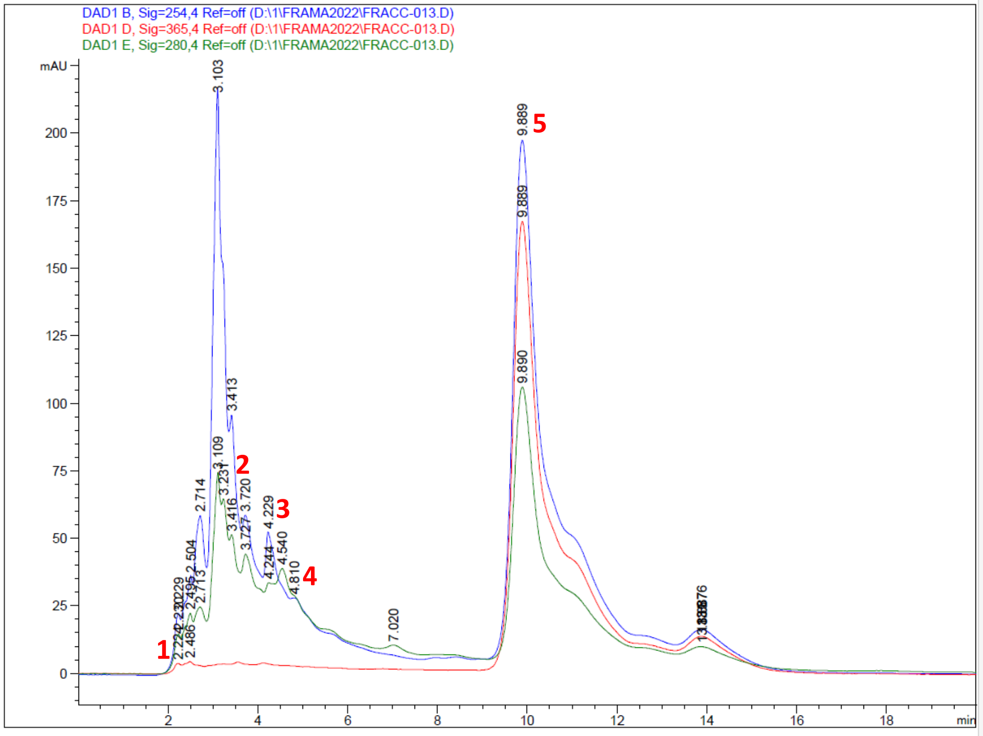

m.- Chromatogram fraction thirteen (1.- Catechin, 2.- Genistein, 3.- Naringin, 4.- Naringenin, 5.- Luteolin).

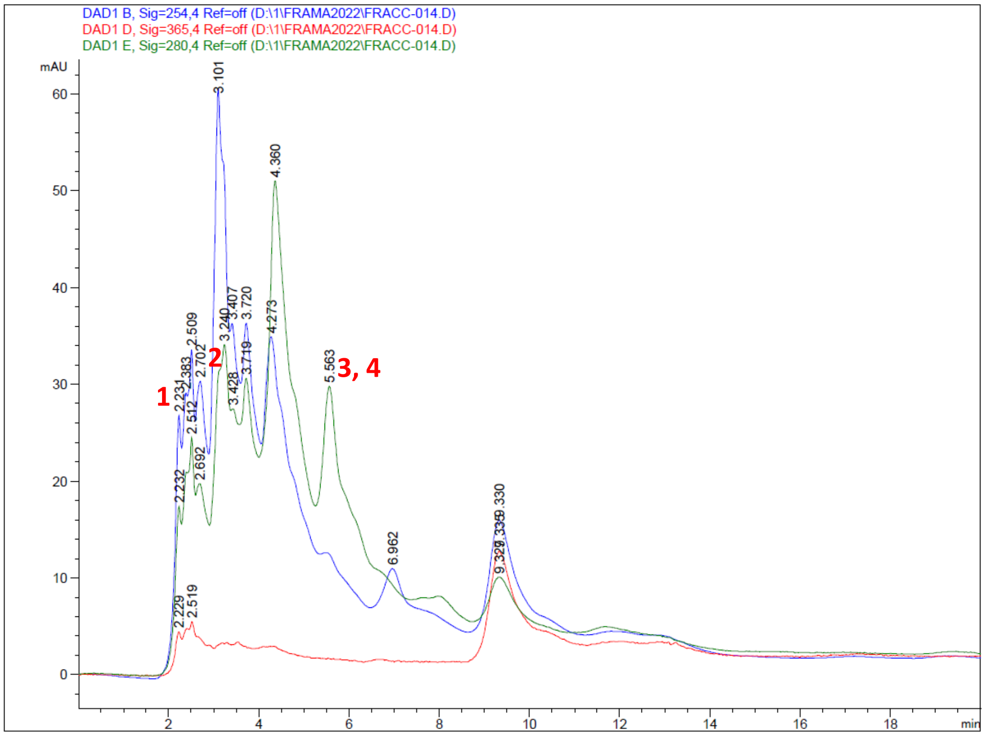

n.- Chromatogram fraction fourteen (1.- Catechin, 2.- Catechol, 3.- Naringin, 4.- Naringenin).

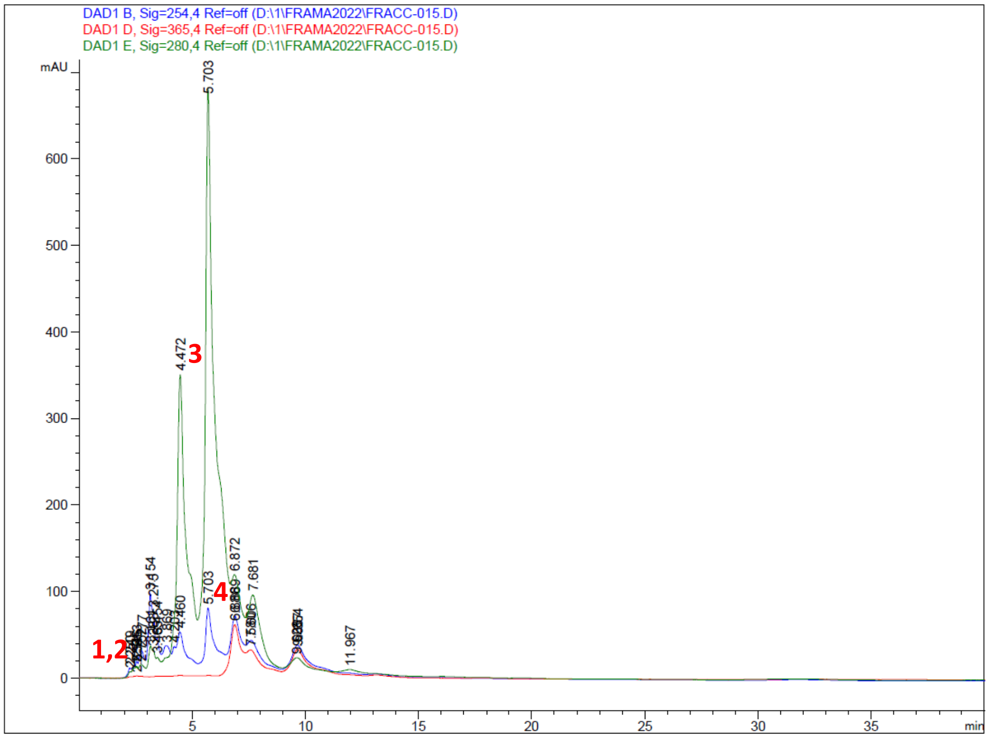

o.- Chromatogram fraction fifteen (1.- Catechin, 2.- Catechol, 3.- Naringin, 4.- Naringenin).

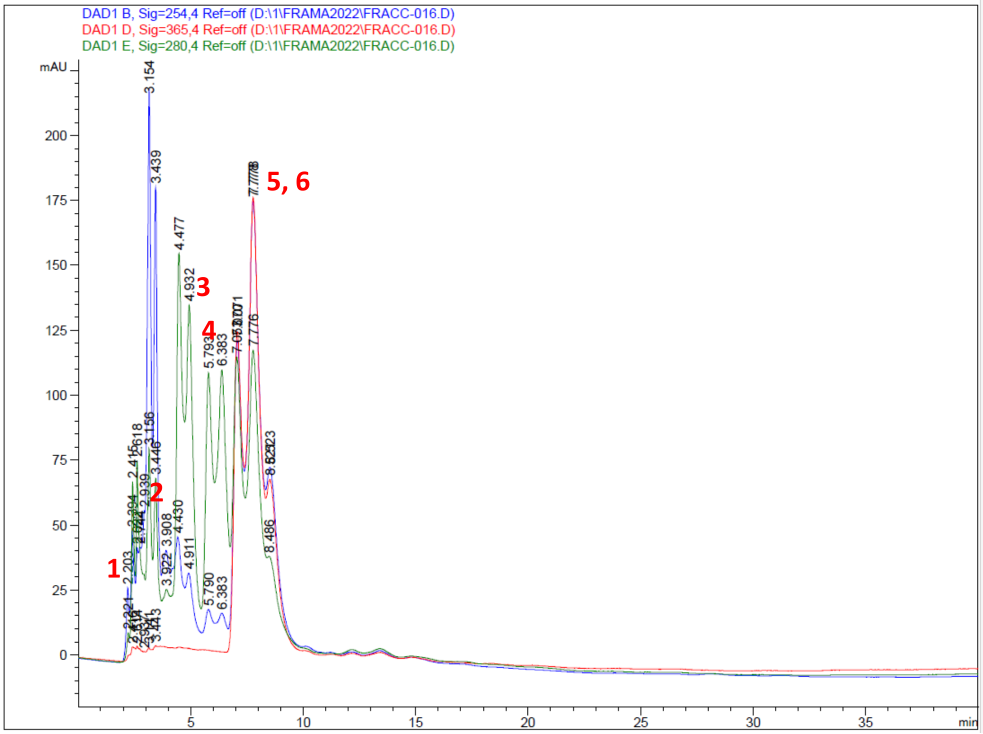

p.- Chromatogram fraction sixteen (1.- Catechin, 2.- Catechol, 3.- Naringin, 4.- Naringenin, 5.- Luteolin, 6.- Apigenin).

.

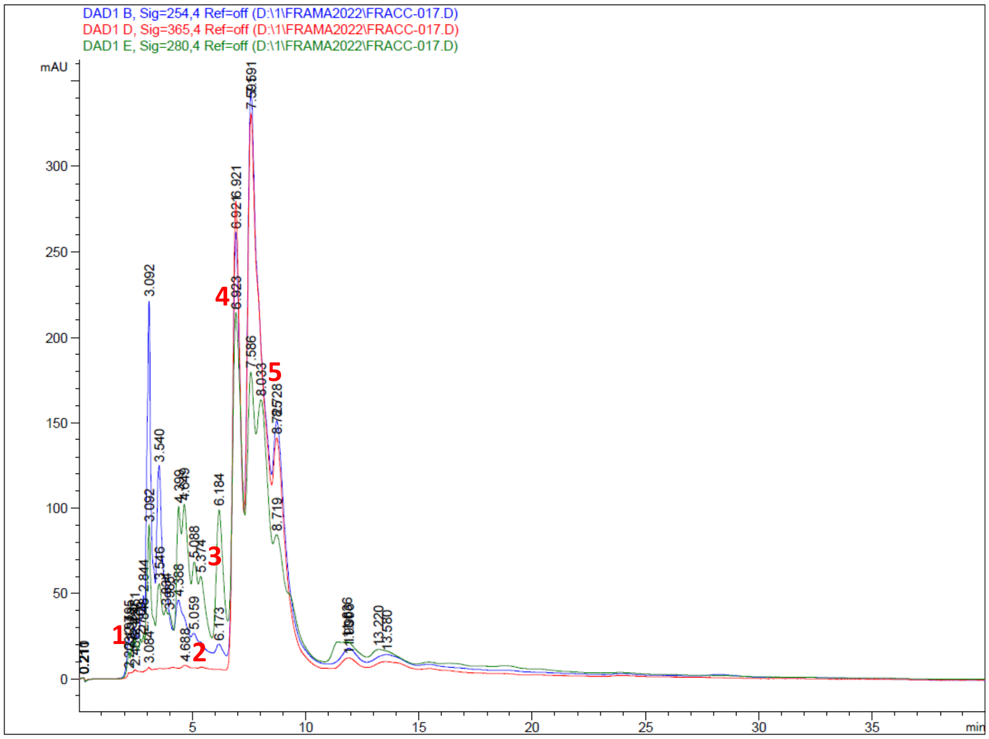

q.- Chromatogram fraction seventeen (1.- Catechin, 2.- Naringin, 3.- Naringenin, 4.- Apigenin, 5.- Luteolin.)

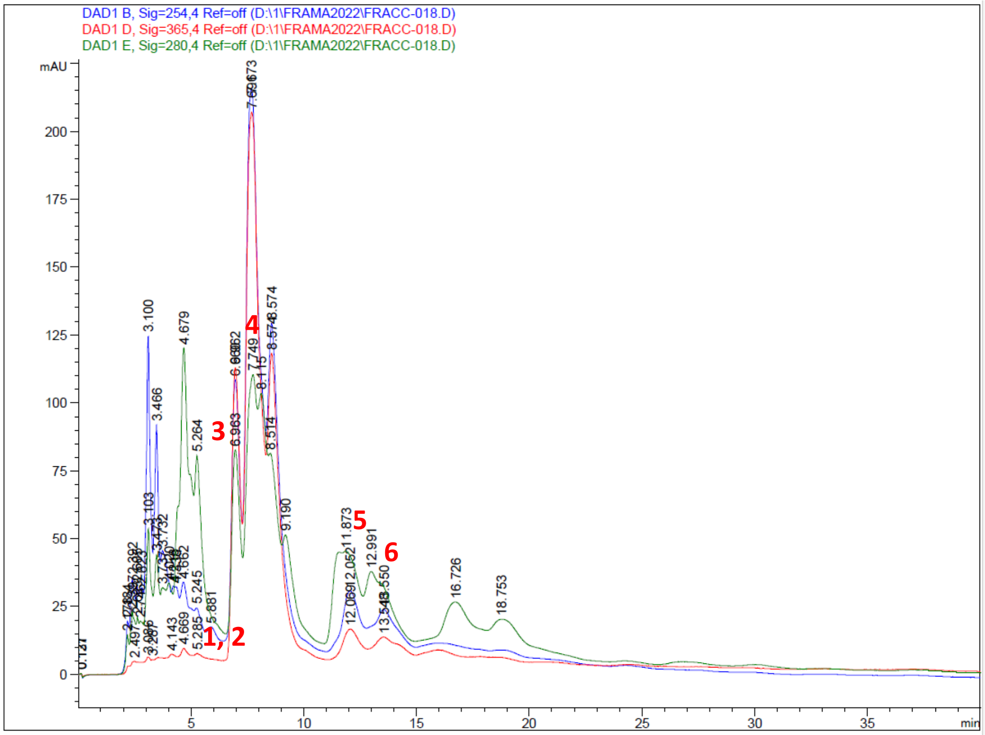

r.- Chromatogram fraction eighteen(1.- Naringin, 2.- Naringenin, 3.- Apigenin, 4.- Luteolin, 5.- Pinocembrin, 6.- Chrysin).

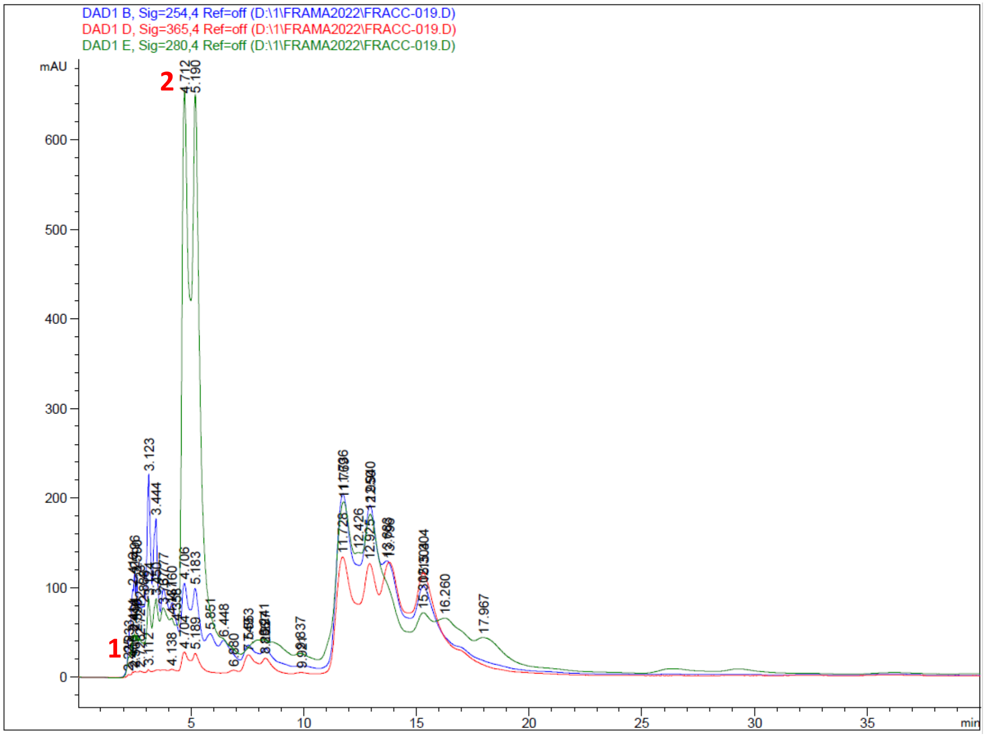

s.- Chromatogram fraction nineteen (1.- Catechin, 2.- Naringenin).

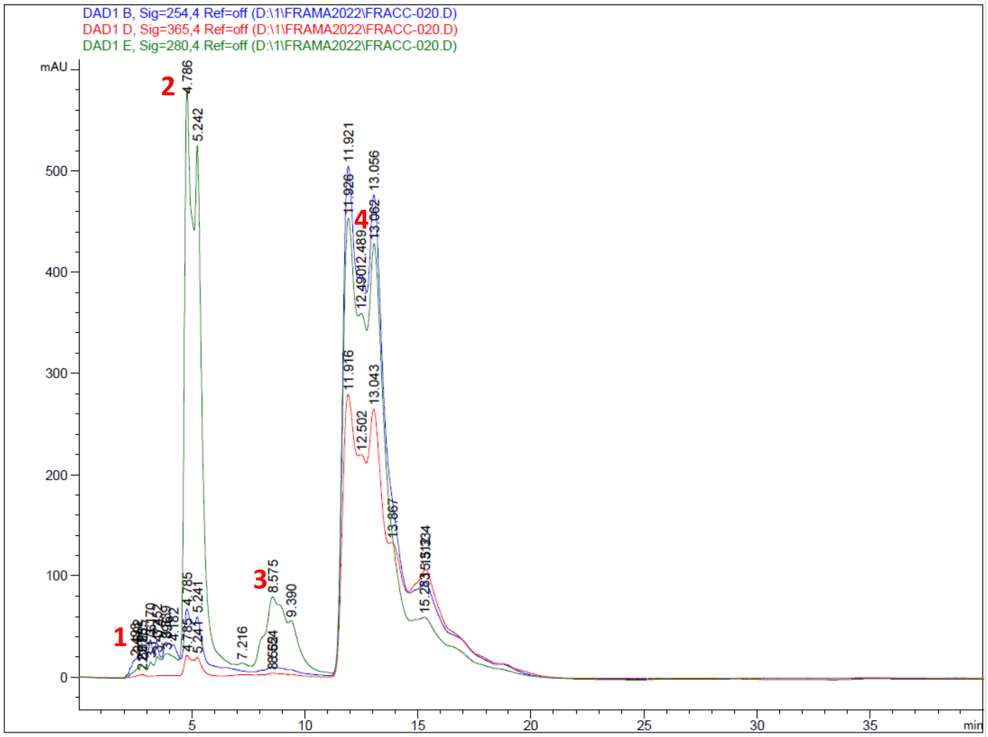

t.- Chromatogram fraction twenty (1.- Catechin, 2.- Naringin, 3.- Naringenin, 4.- Chrysin.).

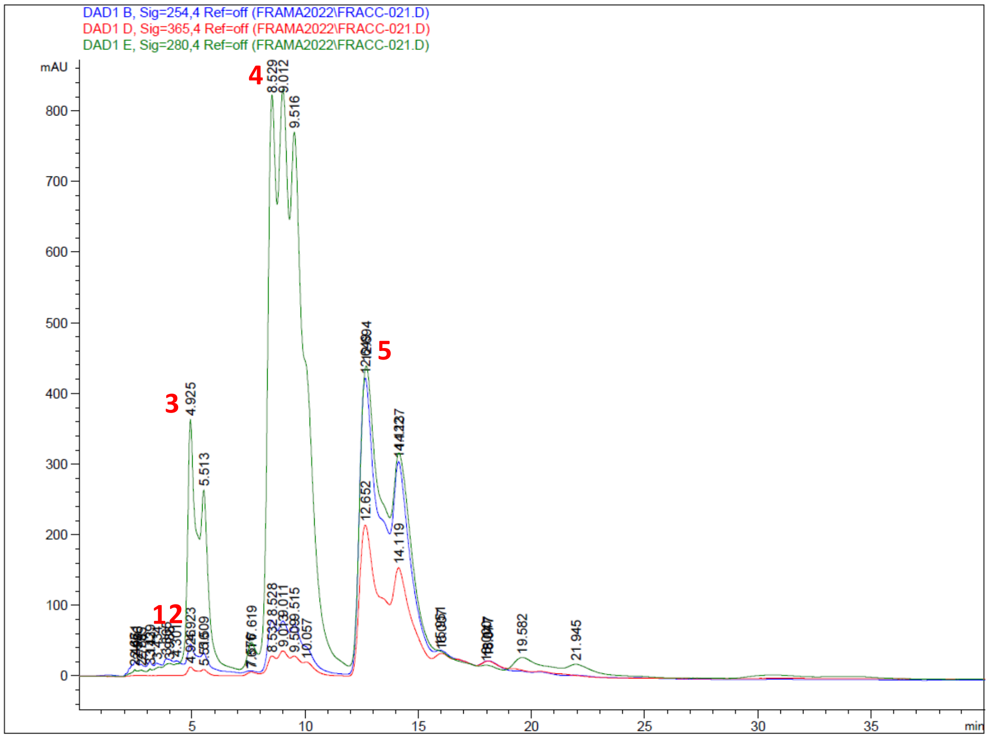

u.- Chromatogram fraction twenty-one (1.- Catechin, 2.- Naringin, 3.- Naringenin, 4.- Pinocembrin, 5.- Chrysin).

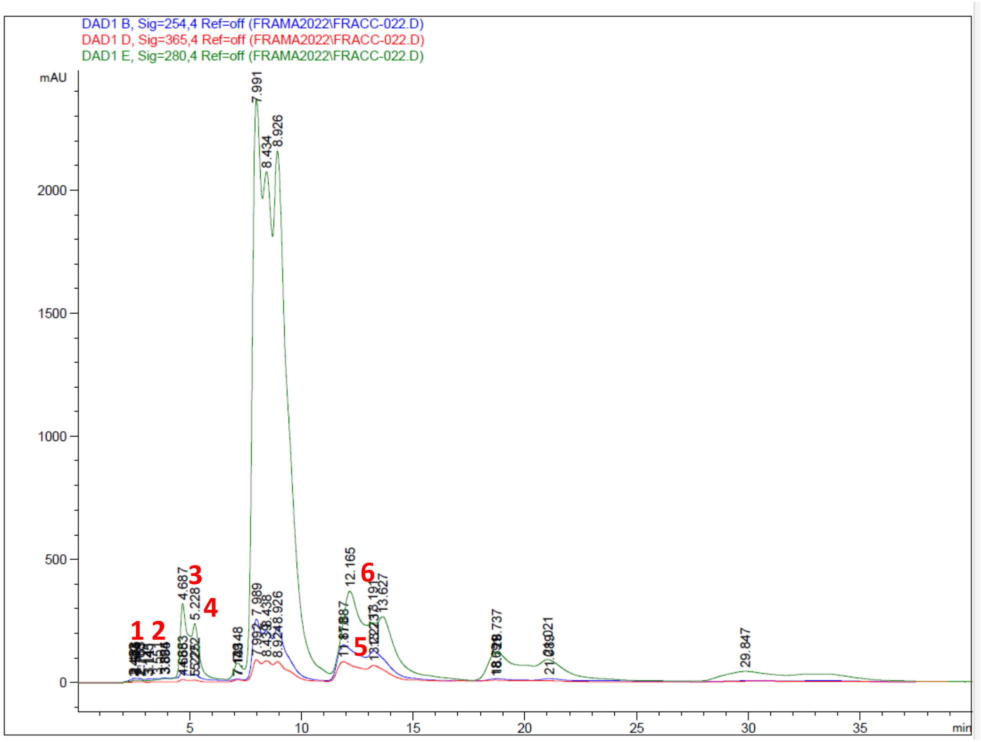

v.- Chromatogram fraction twenty-two (1.- Catechin, 2.- Catechol, 3.-Naringin, 4.- Naringenin, 5.- Baicalein, 6.- Chrysin).

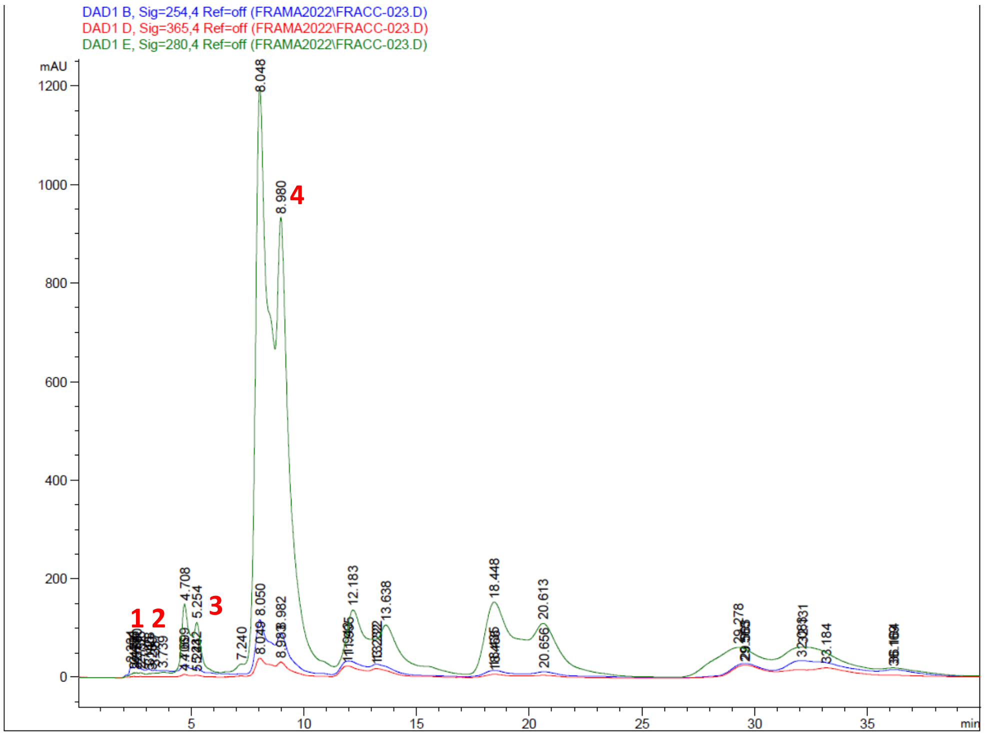

w.- Chromatogram fraction twenty-three (1.- Catechin, 2.- Catechol, 3.- Naringin, 4.- Naringenin).

Figure S3. HPLC–MS chromatograms of the fractions obtained from the EA fraction of ChEEP (a-w).

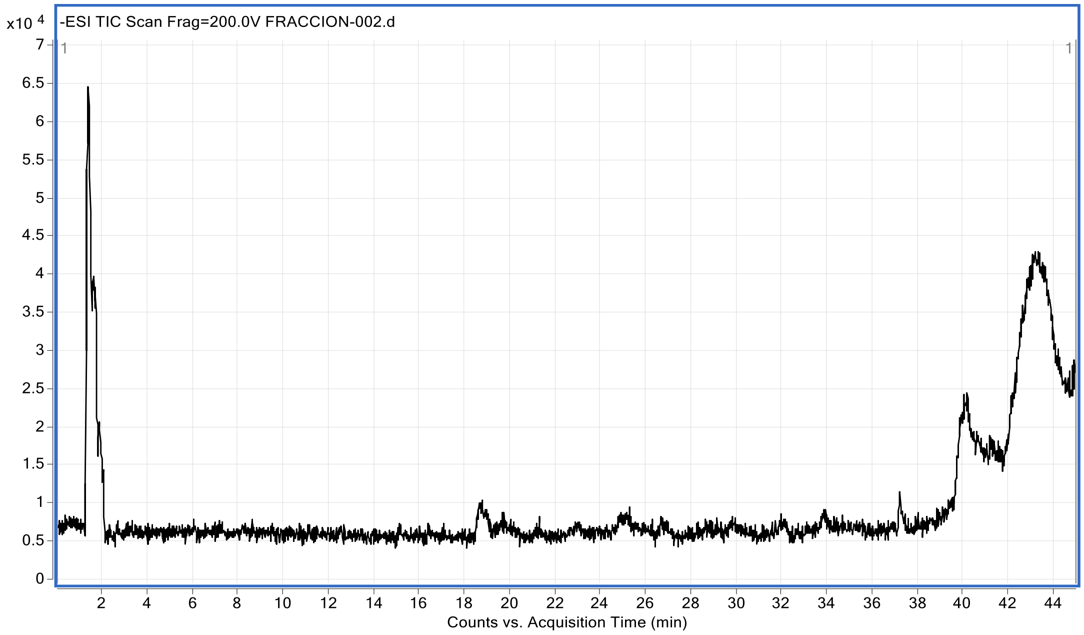

a.- Chromatogram fraction one (Not compounds detected).

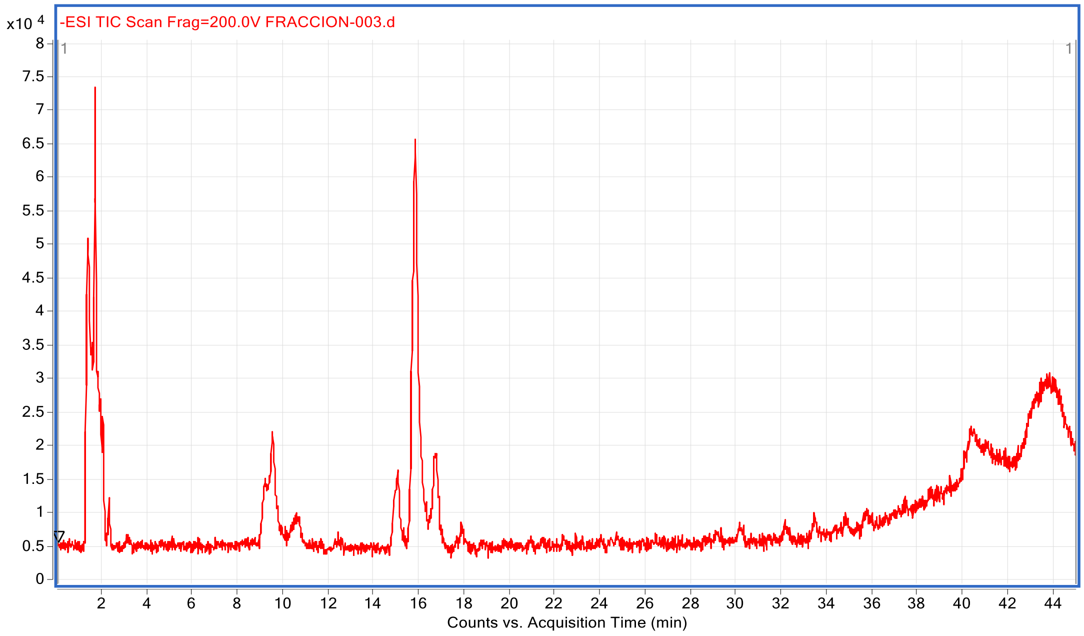

b.- Chromatogram fraction two (Not compounds detected).

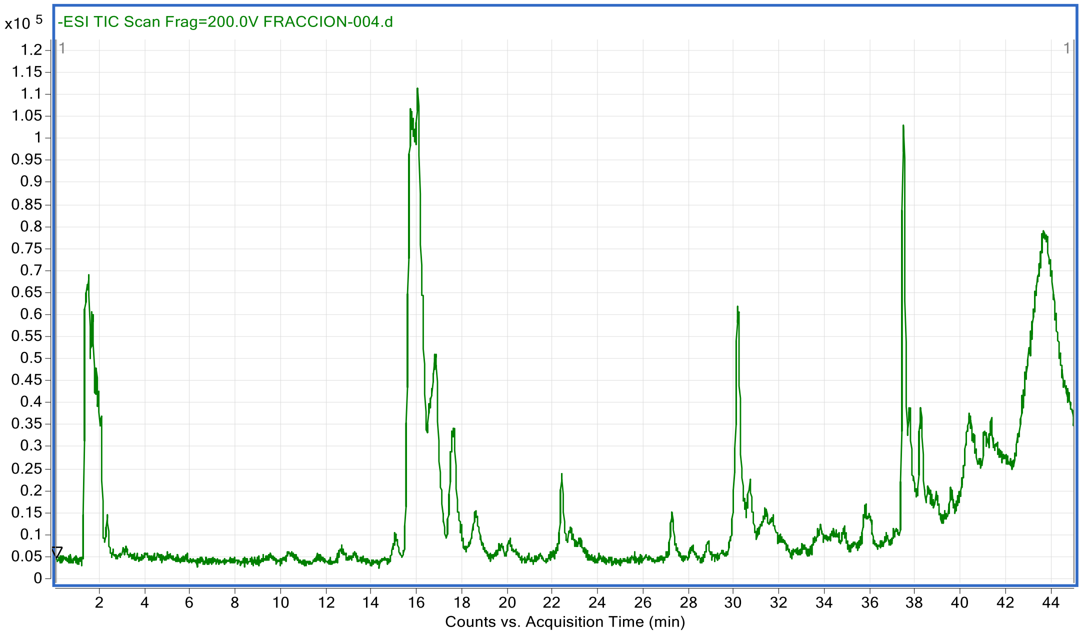

c.- Chromatogram fraction three (Not compounds detected).

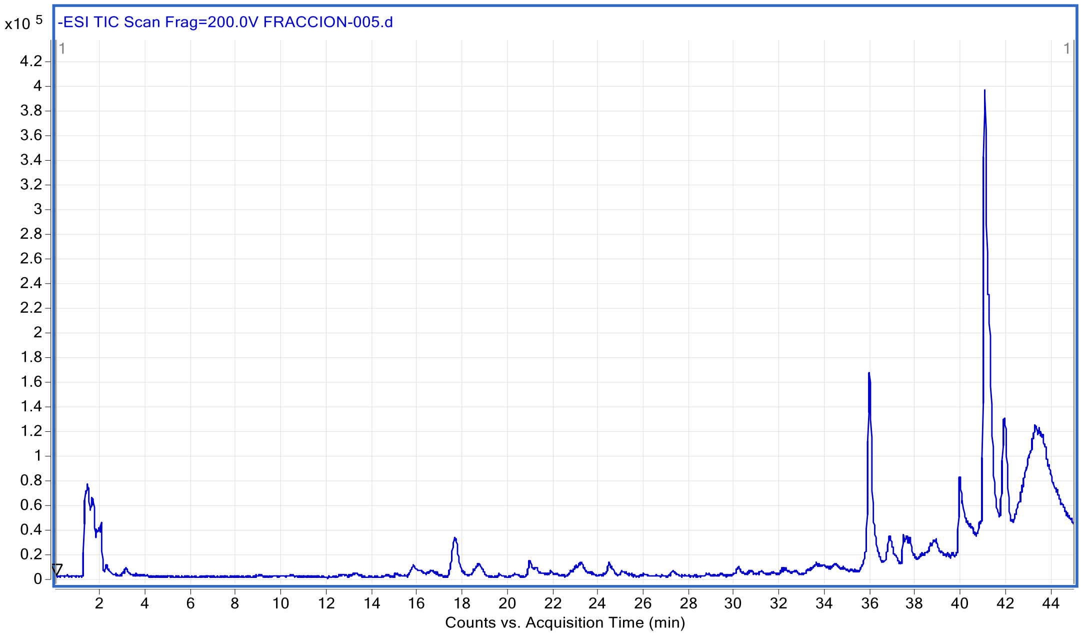

d.- Chromatogram fraction four (Not compounds detected).

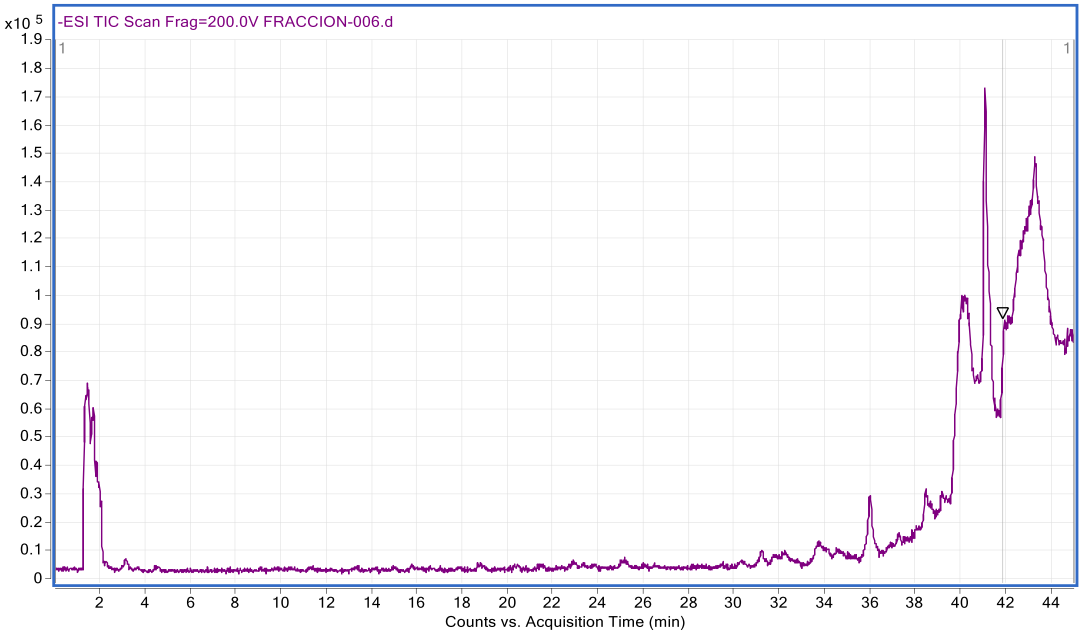

e.- Chromatogram fraction five (Not compounds detected).

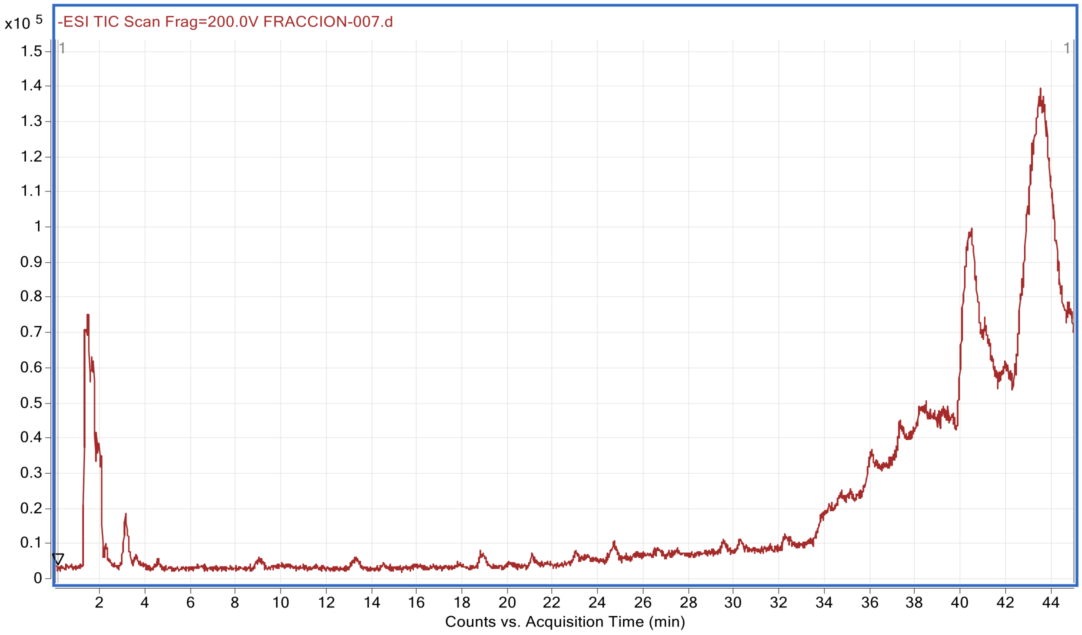

f.- Chromatogram fraction six (Not compounds detected).

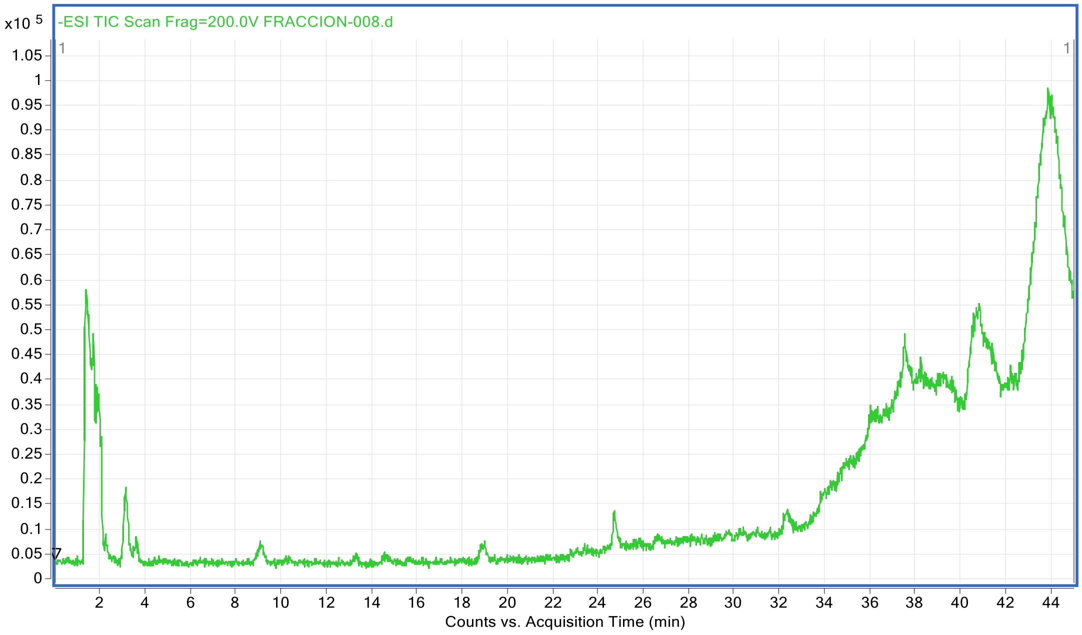

g.- Chromatogram fraction seven (Not compounds detected).

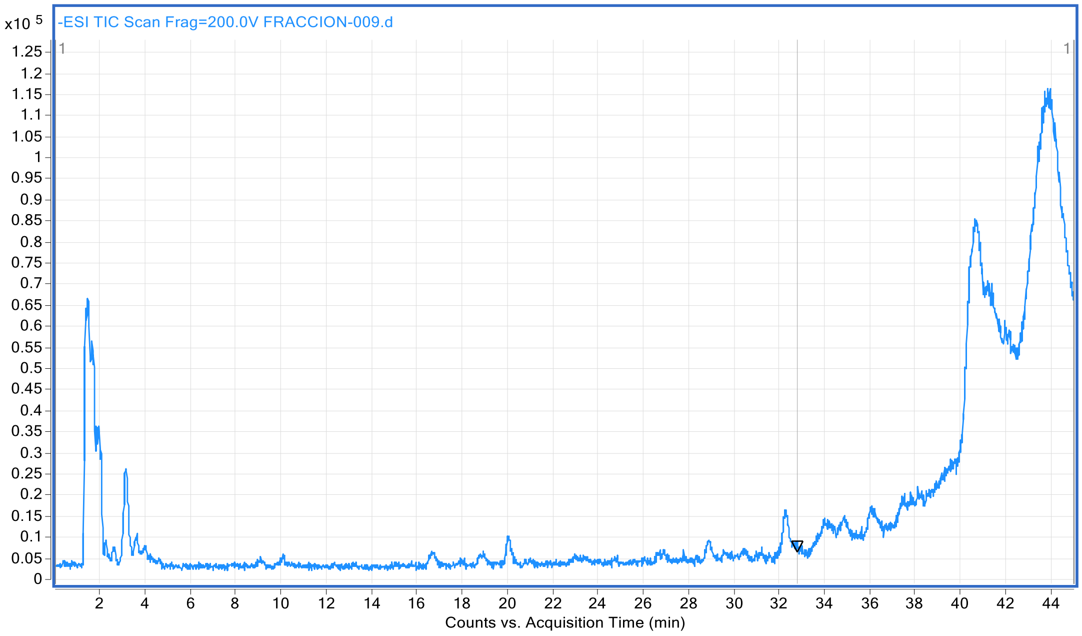

h.- Chromatogram fraction eight (Not compounds detected).

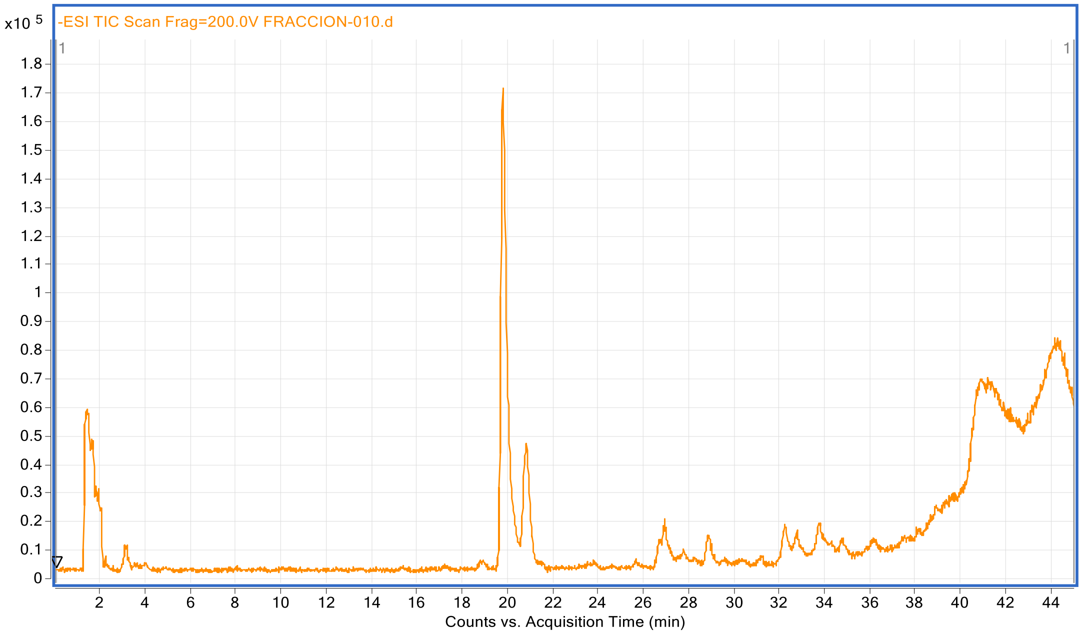

i.- Chromatogram fraction nine (Not compounds detected).

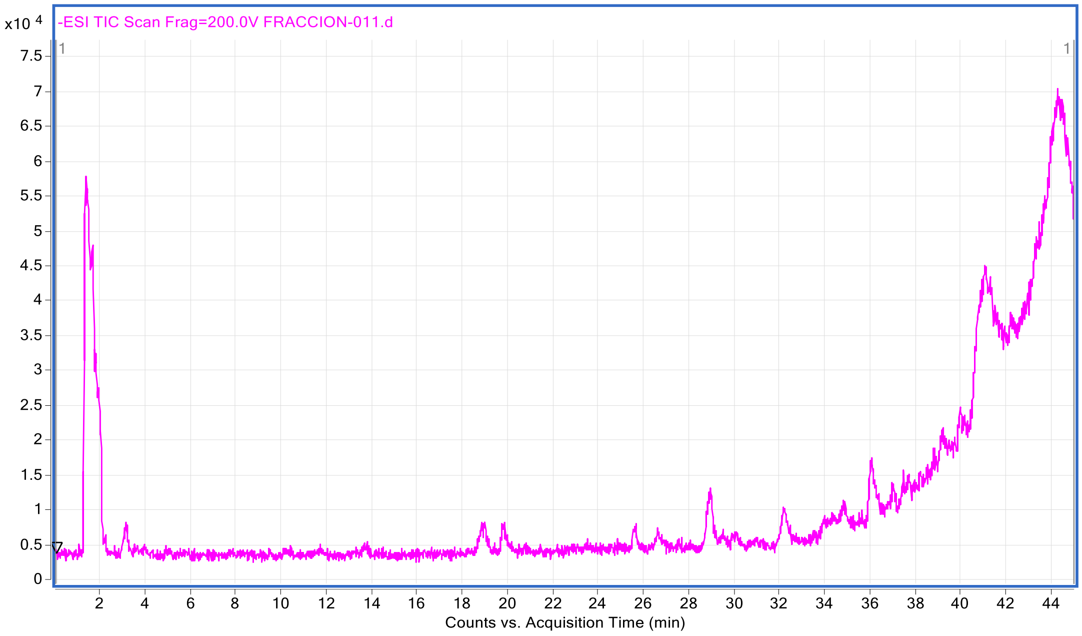

j.- Chromatogram fraction ten (Not compounds detected).

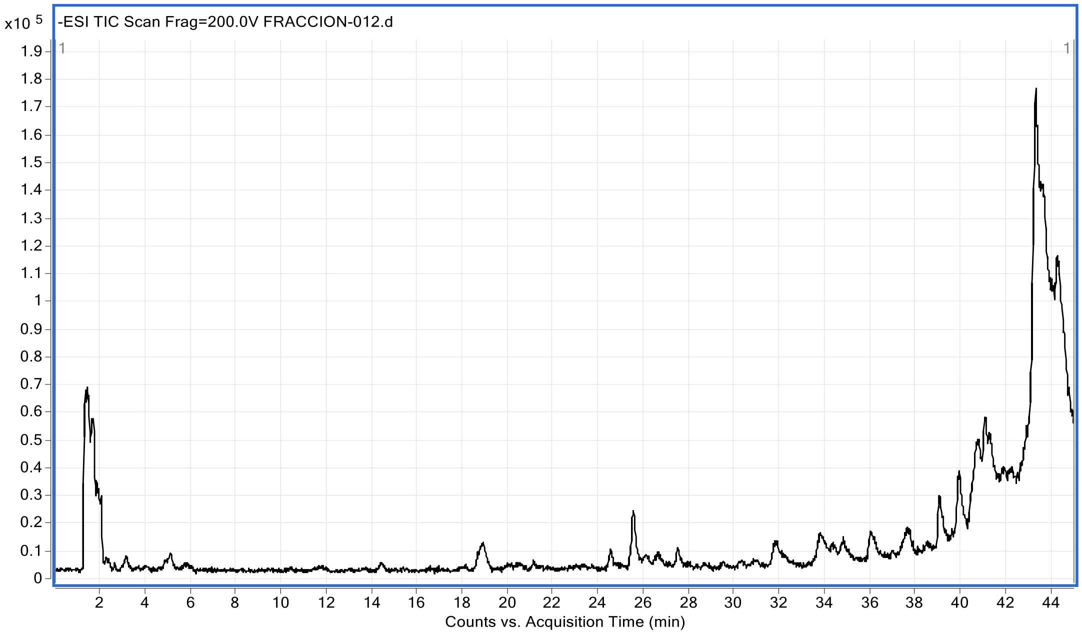

k.- Chromatogram fraction eleven (Not compounds detected).

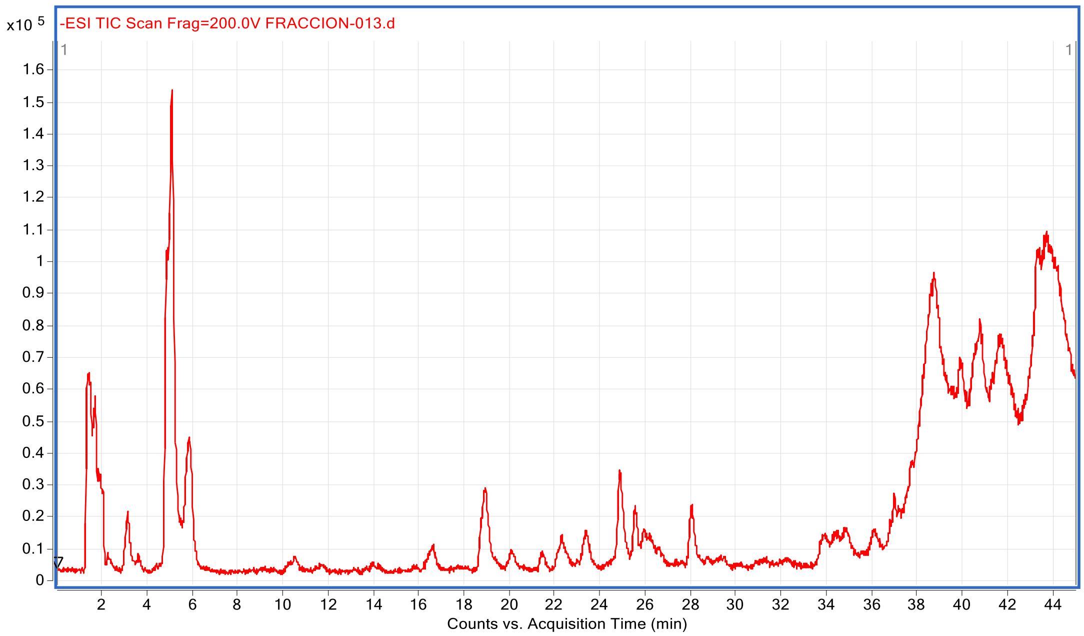

l.- Chromatogram fraction twelve (1. Kaemferol, 2. Pinocembrin, 3. Acacetin).

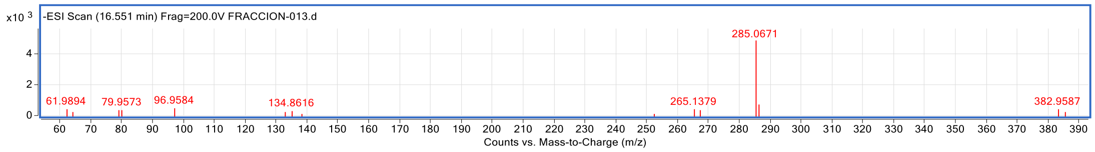

Kaemferol. Error ppm -2.94

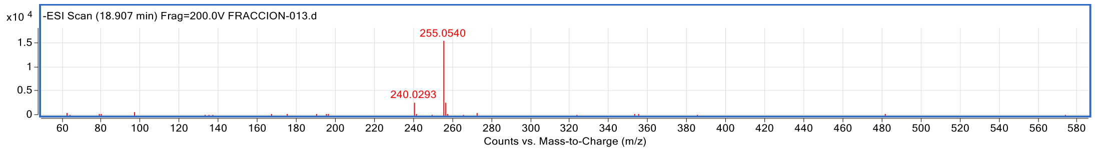

Pinocembrin. Error ppm -11.39

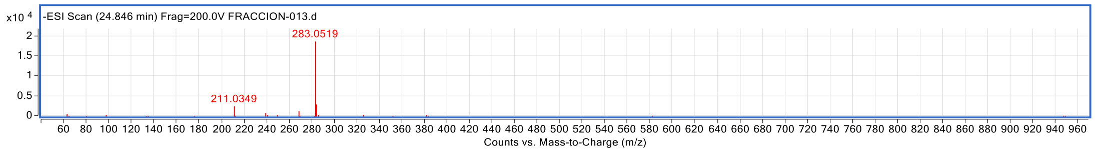

Acacetin. Error ppm -1.79

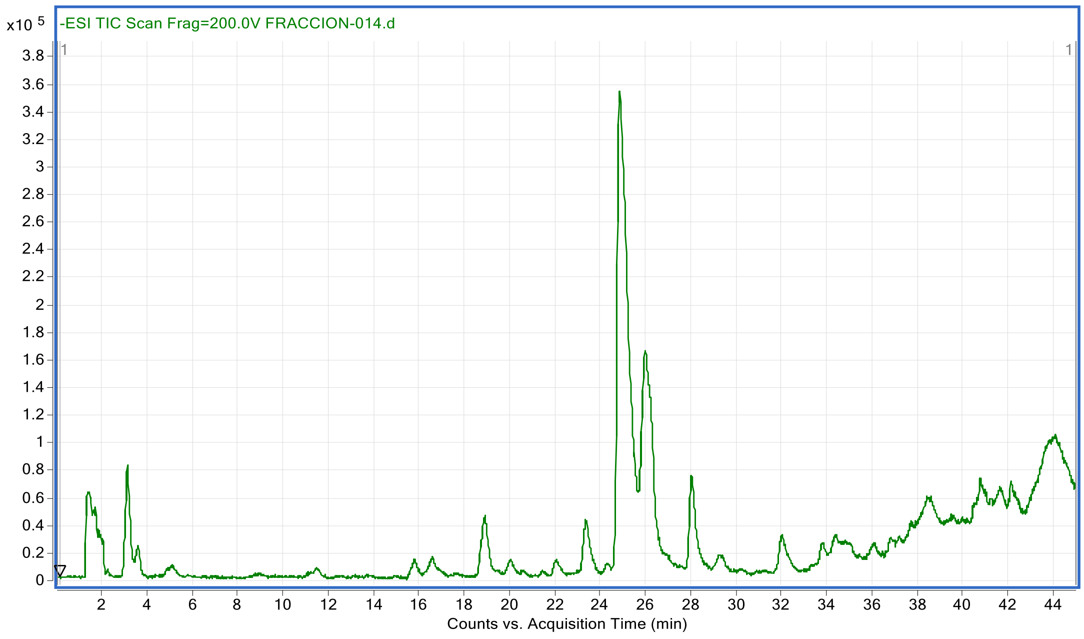

m.- Chromatogram fraction thirtheen (1. Kaemferol, 2. Pinocembrin, 3. Acacetin).

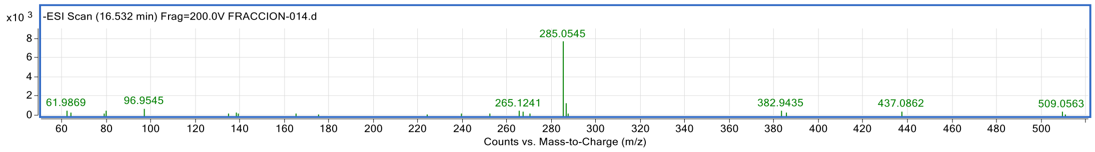

Kaemferol. Error ppm 3.68

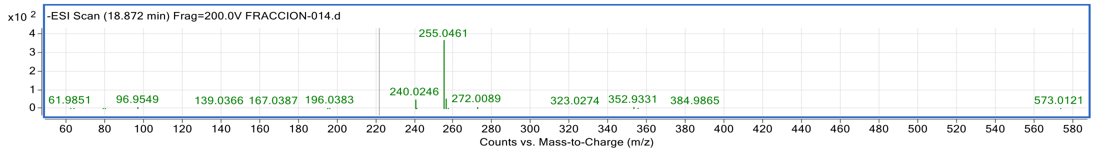

Pinocembrin. Error ppm -2.96

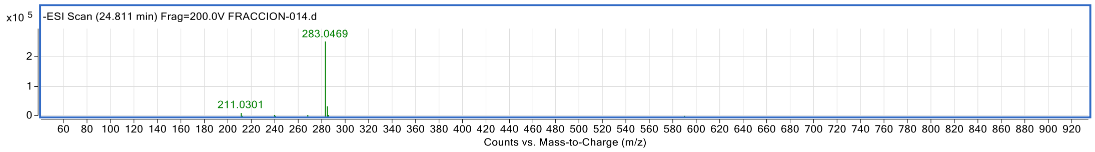

Acacetin. Error ppm -3.26

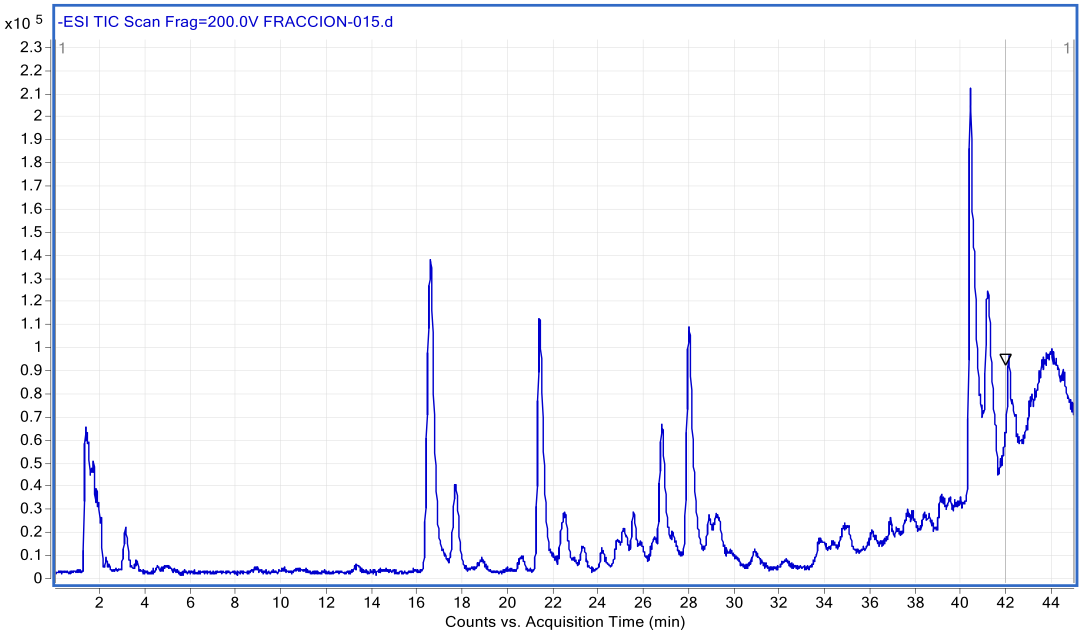

n.- Chromatogram fraction fourtheen (1. Kaemferol, 2. Genistein, 3. Apigenin, 4. Baicalein).

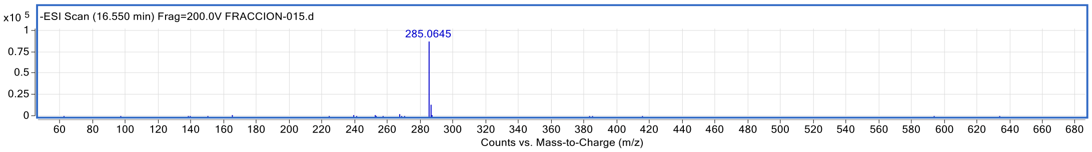

Kaemferol. Error ppm -9.81

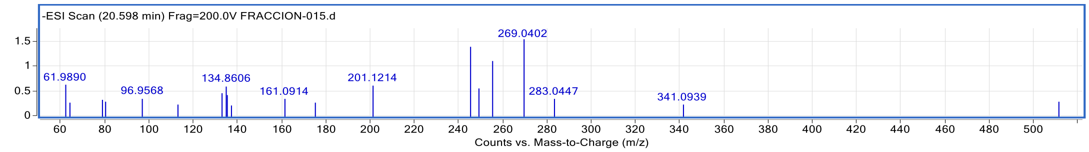

Genistein. Error ppm 4.98

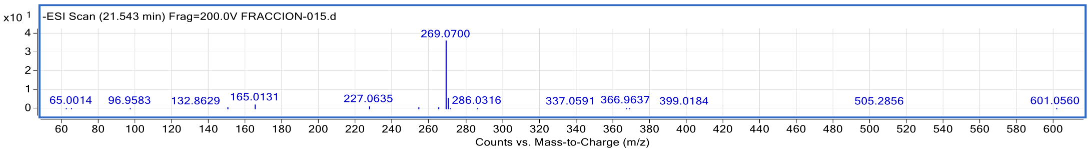

Apigenin. Error ppm -11.81

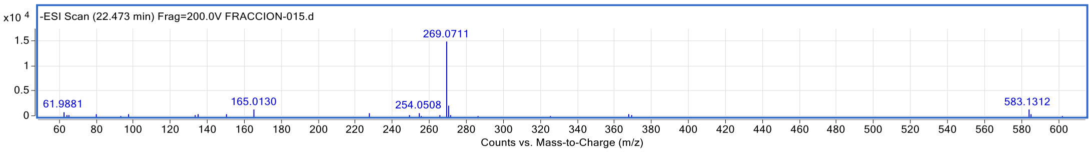

Baicalein. Error ppm 2.94

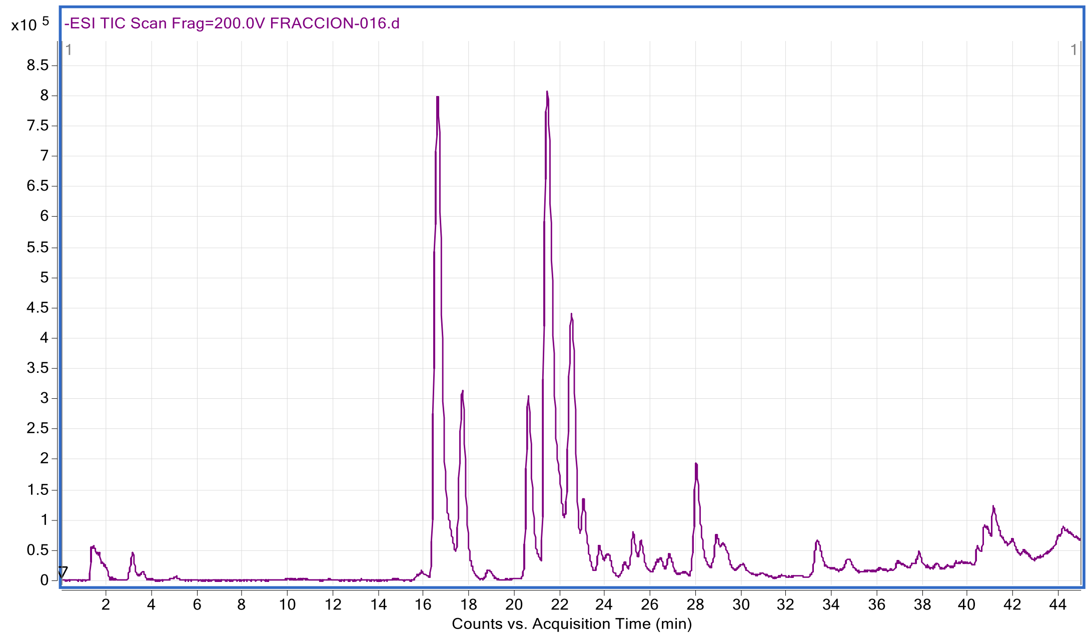

o.- Chromatogram fraction fifteen (1. Kaemferol, 2. Genistein, 3. Apigenin, 4. Baicalein).

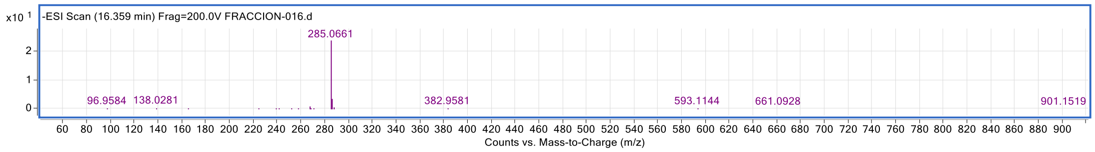

Kaemferol. Error ppm 3.38

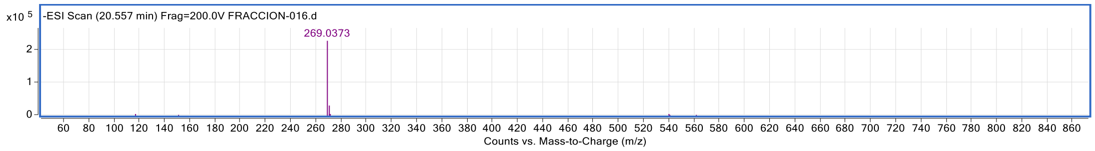

Genistein. Error ppm -6

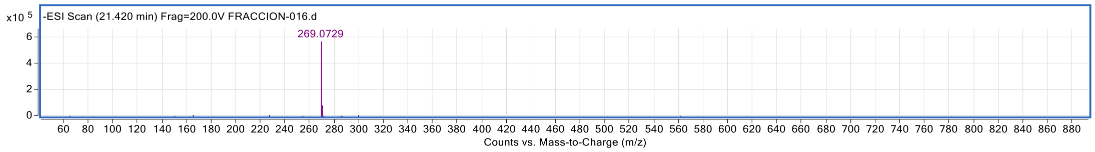

Apigenin. Error ppm -3.05

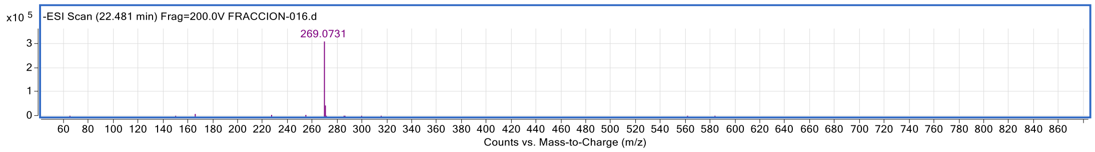

Baicalein. Error ppm -1.57

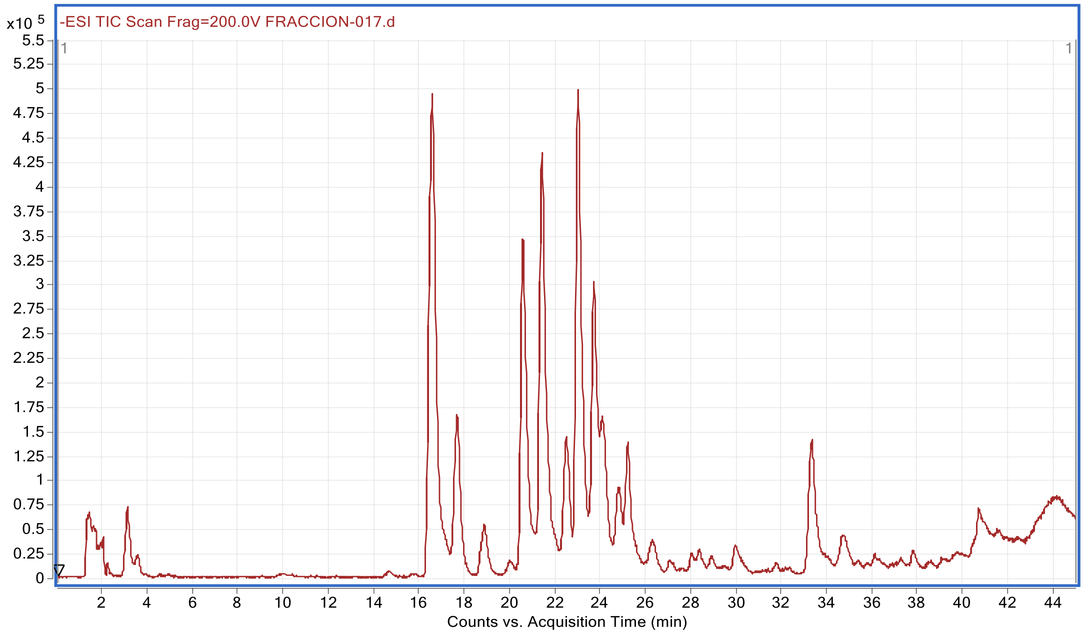

p.- Chromatogram fraction sixteen (1. Kaemferol, 2. Pinocembrin, 3. Genistein, 4. Apigenin, 5. Baicalein).

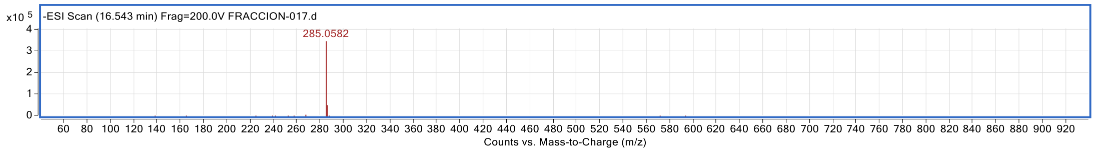

Kaemferol. Error ppm -8.16

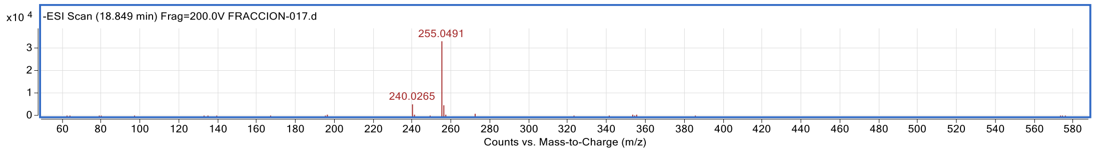

Pinocembrin. Error ppm 7.68

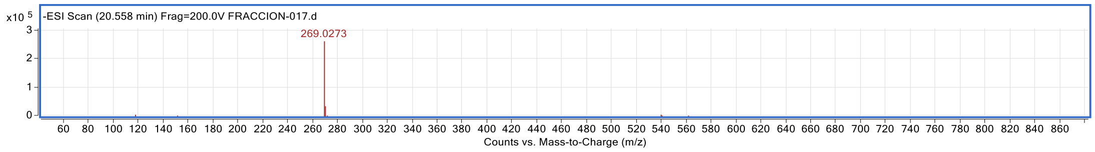

Genistein. Error ppm 11.39

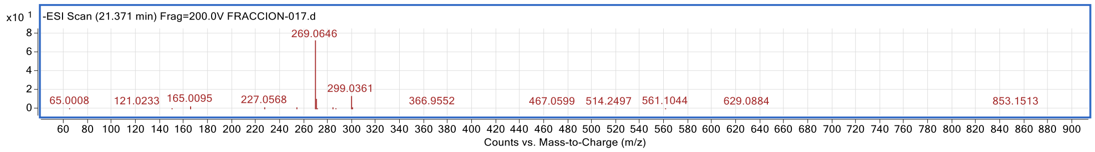

Apigenin Error ppm 12.67

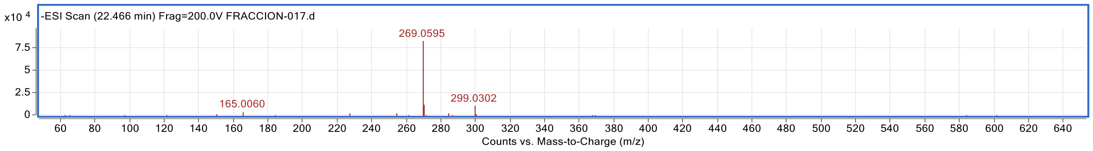

Baicalein. Error ppm -9.98

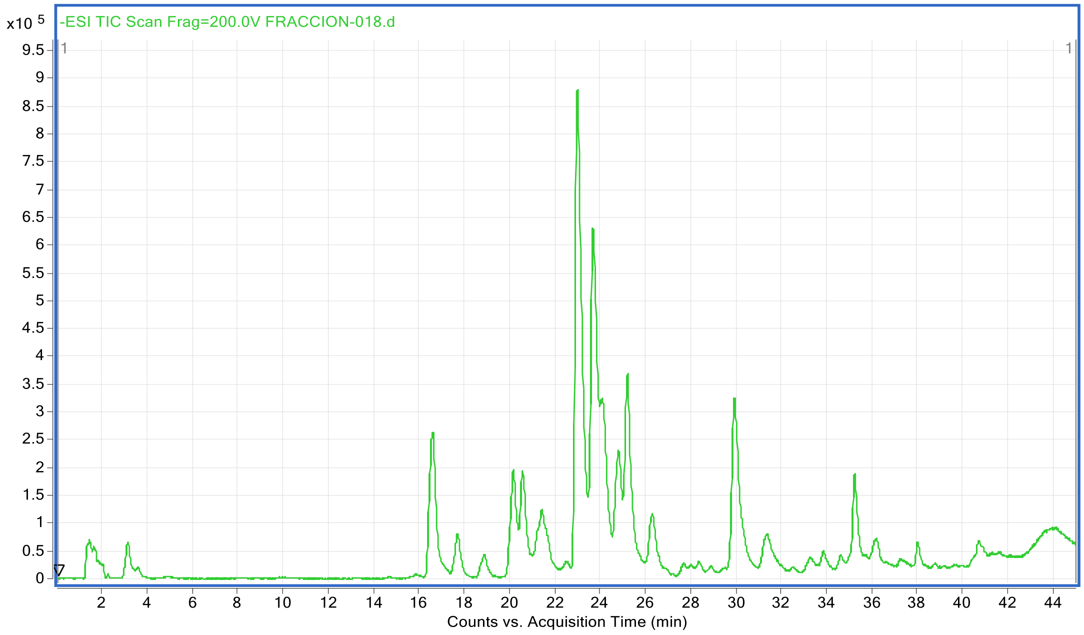

q.- Chromatogram fraction seventeen (1. Kaemferol, 2. Pinocembrin, 3. Naringenin).

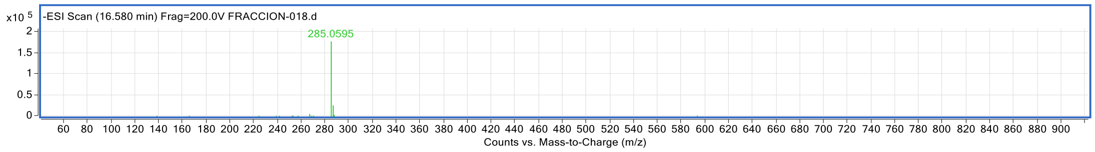

Kaemferol. Error ppm -6.41

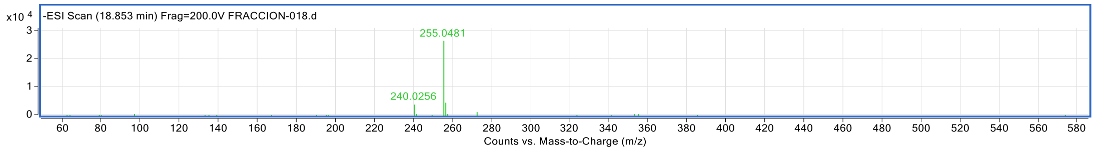

Pinocembrin. Error ppm 9.37

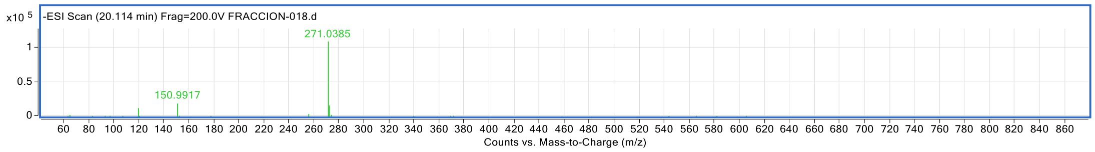

Naringenin. Error ppm 6.34

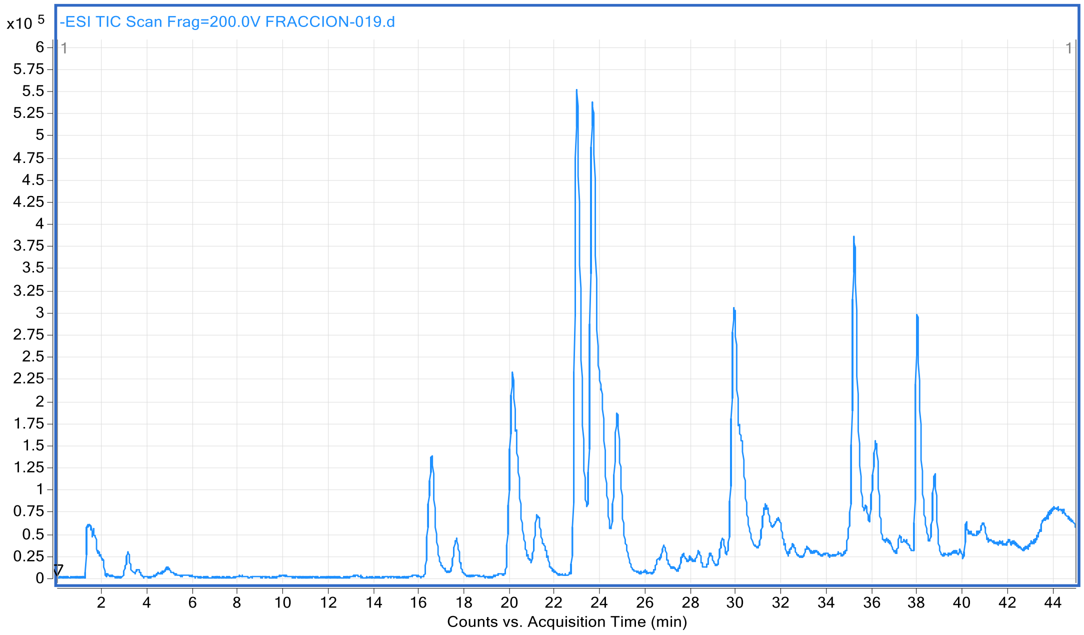

r.- Chromatogram fraction eighteen (1. Kaemferol, 2. Naringenin).

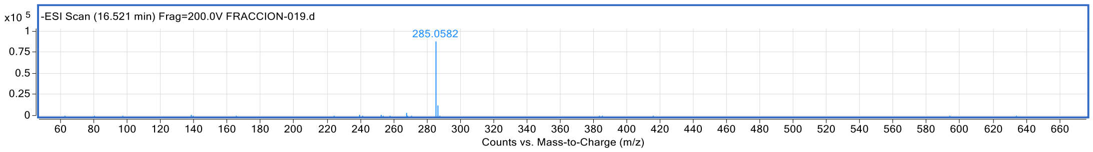

Kaemferol. Error ppm -8.04

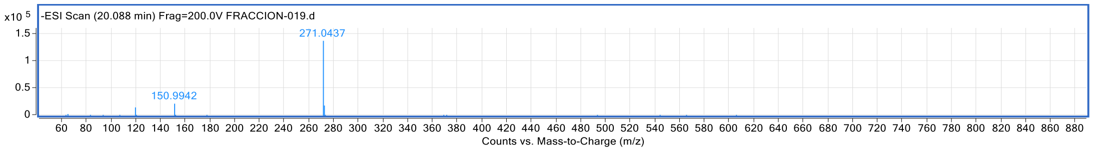

Naringenin. Error ppm 8.55

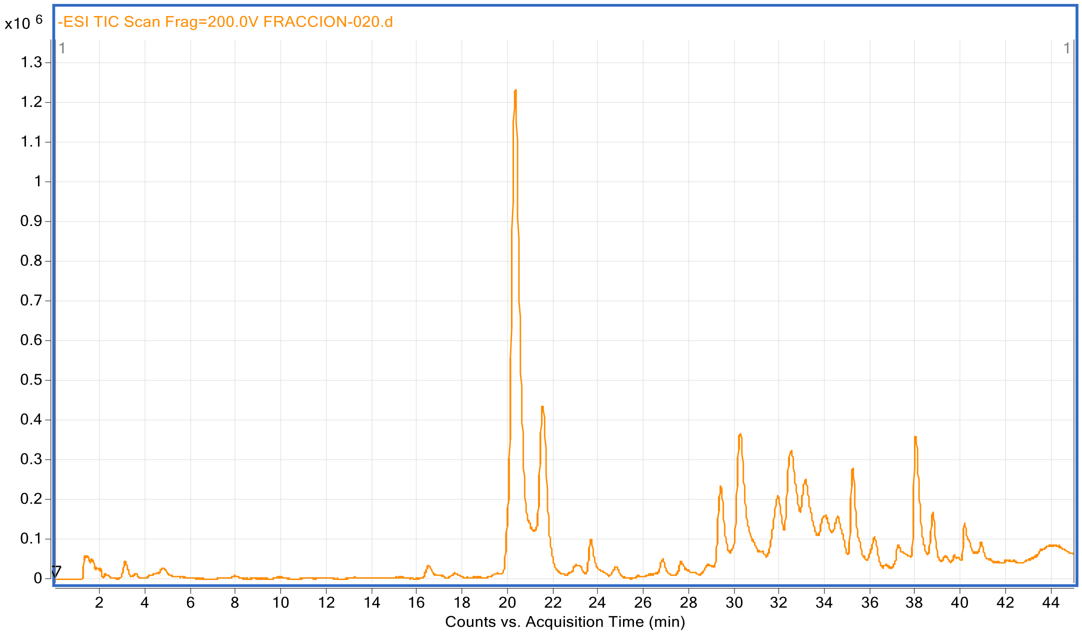

s.- Chromatogram fraction nineteen (1. Naringenin).

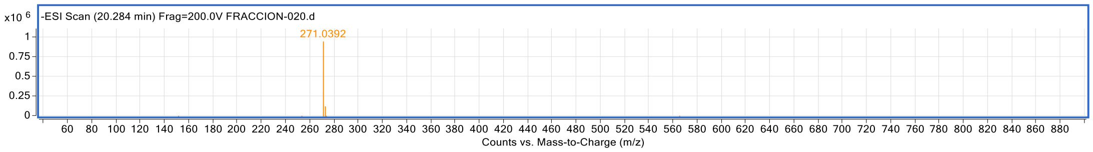

Naringenin. Error ppm 3.38

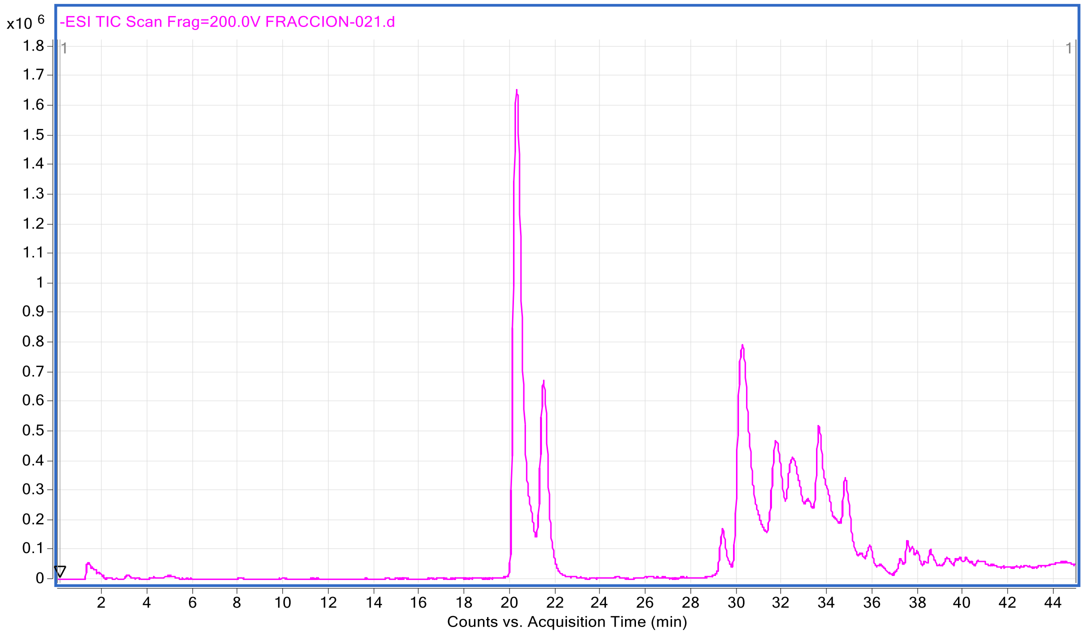

t.- Chromatogram fraction twenty (1. Naringenin).

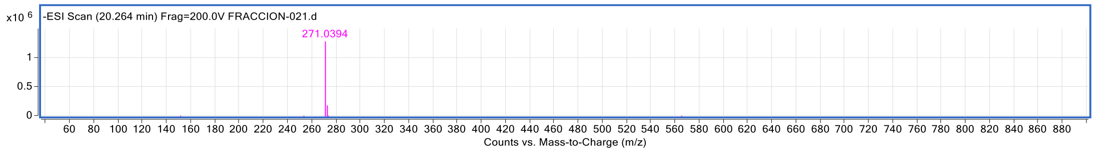

Naringenin. Error ppm 2.59

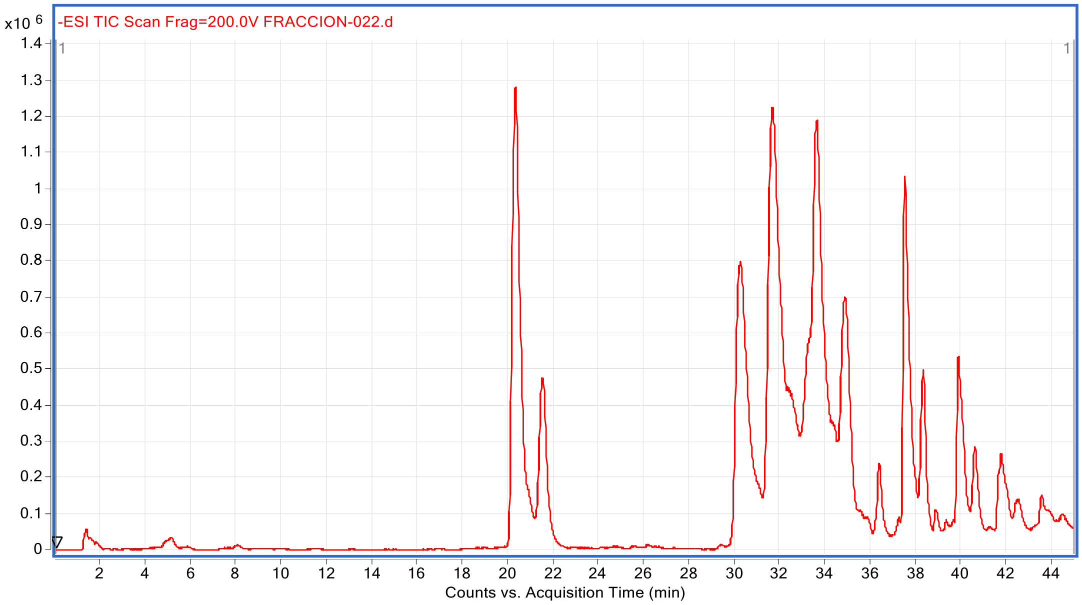

u.- Chromatogram fraction twenty-one (1. Naringenin).

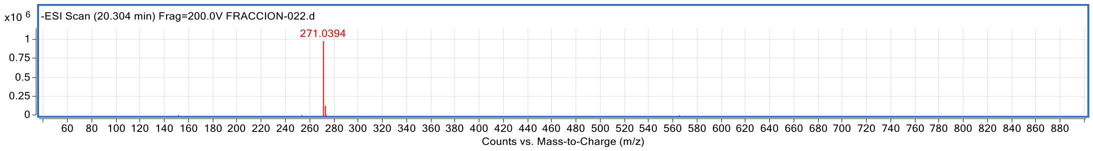

Naringenin. Error ppm 2.92

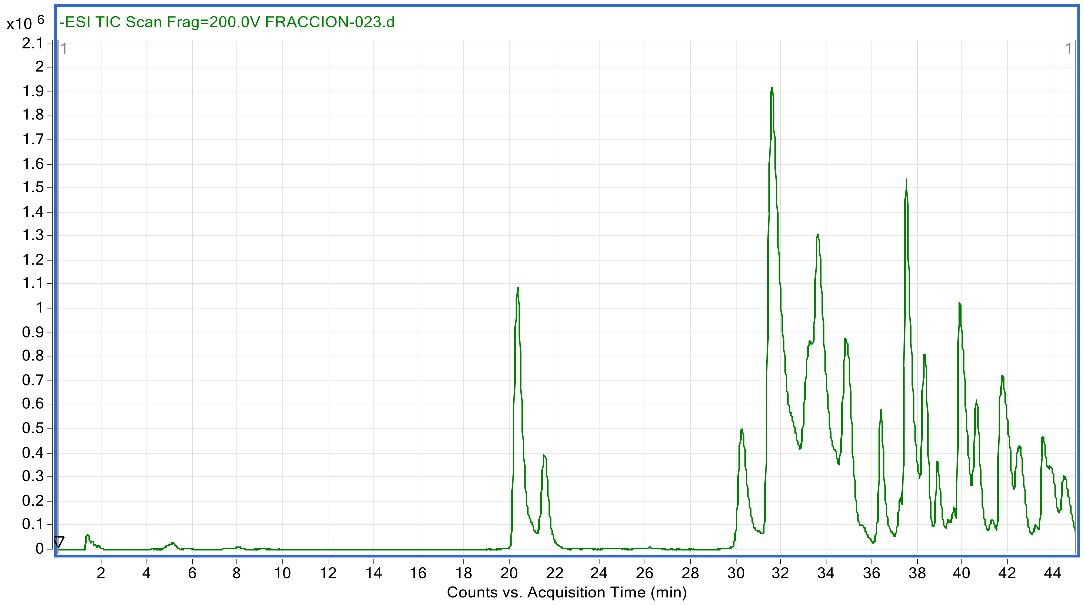

v.- Chromatogram fraction twenty-two (1. Naringenin).

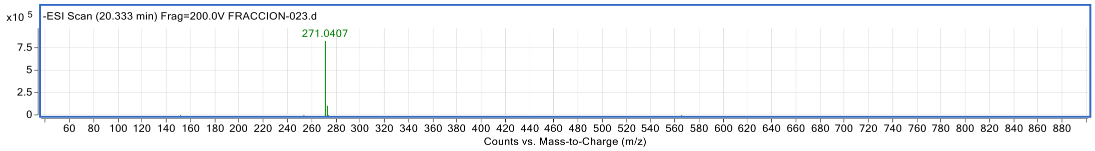

Naringenin. Error ppm -1.81

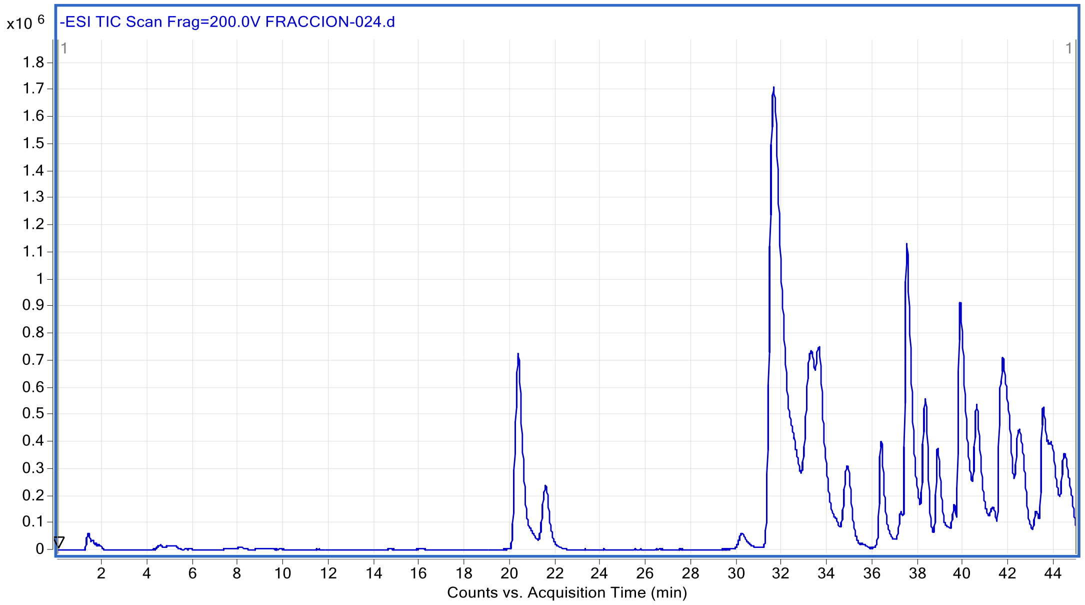

w.- Chromatogram fraction twenty-three (1. Naringenin).

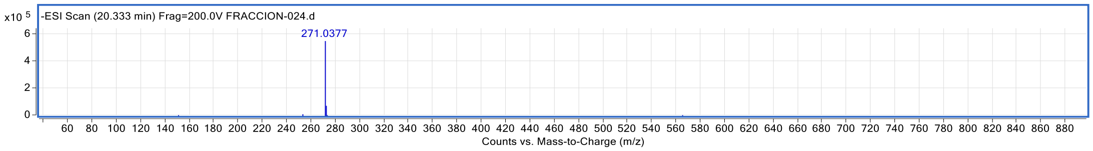

Naringenin. Error ppm 9.06
